# Barriers to cervical cancer and breast cancer screening uptake in low- and middle-income countries: a systematic review

**DOI:** 10.1093/heapol/czac104

**Published:** 2022-12-16

**Authors:** Ananth Srinath, Frits van Merode, Shyam Vasudeva Rao, Milena Pavlova

**Affiliations:** Department of Community Oncology, Sri Shankara National Centre for Cancer Prevention and Research, 1st Cross, Shankara Math Campus, Shankarapuram, Basavanagudi, Bengaluru, Karnataka 560004, India; Department of Health Services Research, Care and Public Health Research Institute, Maastricht University Medical Centre+, Maastricht University, P.O. Box 616, Maastricht, MD 6200, The Netherlands; Department of Health Services Research, Care and Public Health Research Institute, Maastricht University Medical Centre+, Maastricht University, P.O. Box 616, Maastricht, MD 6200, The Netherlands; Faculty of Health, Medicine and Life Sciences, Maastricht University Medical Centre+, Maastricht University, P. Debyelaan 25, Maastricht, HX 6229, The Netherlands; Faculty of Health, Medicine and Life Sciences, Maastricht University Medical Centre+, Maastricht University, P. Debyelaan 25, Maastricht, HX 6229, The Netherlands; Forus Health Private Limited, #2234, 23rd Cross, Banashankari 2nd Stage, Bengaluru, Karnataka 560070, India; Department of Health Services Research, Care and Public Health Research Institute, Maastricht University Medical Centre+, Maastricht University, P.O. Box 616, Maastricht, MD 6200, The Netherlands

**Keywords:** Systematic literature review, barriers to breast and cervical screening, accessibility, low- and middle-income countries

## Abstract

There is an alarmingly high growth in breast and cervical cancers in low- and middle-income countries. Due to late presentation to doctors, there is a lower cure rate. The screening programmes in low- and middle-income countries are not comprehensive. In this paper, we systematically analyse the barriers to screening through an accessibility framework. We performed a systematic literature search in PubMed, Mendeley and Google Scholar to retrieve all English language studies (quantitative, qualitative and mixed-methods) that contained information on breast and cervical cancer screening in low- and middle-income countries. We only considered publications published between 1 January 2016 and 31 May 2021. The review was guided by Preferred Reporting Items for Systematic Reviews and Meta-Analyses literature search extension (PRISMA-S), an extension to the PRISMA Statement for Reporting Literature Searches in Systematic Reviews. The search yielded a total of 67 articles from low- and middle-income countries in this review. We used a framework on accessibility known as the 5A framework, which distinguishes five aspects of access: approachability, acceptability, availability, affordability and appropriateness, to classify the screening barriers. We added two more aspects: awareness and angst, as they could explain other important barriers to screening. They confirmed how the lack of awareness, cost of the screening service and distance to the screening centre act as major impediments to screening. They also revealed how embarrassment and fear of screening and cultural factors such as lack of spousal or family support could be obstacles to screening. We conclude that more needs to be done by policymakers and governments to improve the confidence of the people in the health systems. Women should be made aware of the causes and risk factors of cancer through evidence-based strategies so that there is an increased adherence to screening.

Key messages

- Fear of screening test results, lack of knowledge about the disease and screening, distance to the screening clinic, embarrassment to undergo screening, lack of support or permission from husbands to undergo screening and high cost of screening were the most common barriers. Barriers to treatment and socio-cultural factors have a major influence on screening uptake.

## Introduction

Every year, there are >2.26 million new breast cancer cases and >600 000 new cervical cancer cases worldwide. Over 91% of deaths from cervical cancer and >79% of deaths from breast cancer occur in low- and middle-income countries (Globocan, 2020). The incidence of both cancer types in low- and middle-income countries has tripled in the last decade (University of Washington, 2021). Due to delays in detection, women often present themselves to the clinician at an advanced stage leading to lower cure rates and higher mortality (Bellanger *et al.*, 2018). Early detection and diagnosis through screening increase the chances of treatment and survival (World Bank, 2021). The lack of awareness, lack of health infrastructure or screening facilities, hesitancy to seek health care, poverty and societal apathy severely limit a woman’s choice to seek early intervention (Rivera-Franco and Leon-Rodriguez, 2018). In addition, the incidence of early-onset breast and cervical cancers in low- and middle-income countries is increasing, and evidence shows that early-onset cancer is far more aggressive than the late onset of the disease (Francies *et al.*, 2020).

So far, studies on barriers to breast and cervical cancer screening in low- and middle-income countries suggest that the level of knowledge on these health issues and factors that provide opportunities for knowledge acquisition, such as level of education and employment, determines the screening uptake in women (Islam *et al.*, 2017). Other studies show that intrapersonal and organizational factors are the most important factors that influence women to undergo cervical cancer screening (Faradisa *et al.*, 2020). Some studies have tried to categorize the barriers to breast and cervical cancer screening and have concluded that the lack of knowledge and awareness, psychological barriers such as fear and embarrassment, structural barriers such as the lack of time and socio-cultural and religious barriers such as the lack of family support were the most commonly reported barriers (Devarapalli *et al.*, 2018).

Unlike the high-income countries, the screening programmes for breast and cervical cancers in low- and middle-income countries are unorganized as there are no comprehensive cancer control programmes (Shah *et al.*, 2019). Screening is mostly ad-hoc, and the coverage is not uniform. These countries also lack the resources for preventive screening for early detection as well as resources for adequate treatment, which is linked to a higher mortality rate compared with high-income countries. People sometimes tend to postpone small costs of health-care services even if they are convinced of the benefits (Banerjee and Duflo, 2011). As these costs must be borne by them immediately, while the benefits will accrue over a period, they are inclined to choose temporary gratification over long-term benefits. In order to overcome this problem, the government is expected to design essential health programmes, such as cancer screening programmes, that are free of charge. As mentioned by Thaler and Sunstein (2008), policymakers must design nudges for people to obtain preventive care. Prevention needs to be the natural default option, and incentives should be offered as compensation for prevention actions, in this case, undergoing cancer screening. There is a need to understand not only whether the lack of free cancer screening is a barrier to use but also whether the lack of incentives or nudges is a significant barrier to cancer prevention.

This study analyses the barriers to access to breast and cervical cancer screening in low- and middle-income countries. It systematically reviews the evidence between 1 January 2016 and 31 May 2021 to update and extend the previous review on this topic by Islam *et al.*, (2017). Specifically, we based our review on the 5A framework of Levesque *et al.*, (2013), which distinguishes five aspects of access:

- Approachability, which refers to whether the screening was promoted by the providers and the right information about screening was given to prospective patients, whether the screening was properly organized without waiting times and if the providers enabled transparency and trust.
- Acceptability, which reflects whether the screening was acceptable to the women or whether they had reservations about undergoing screening, the prioritization that they attributed to the screening, their personal beliefs about it and whether they had the autonomy to decide if they wanted the screening and if the partner and relatives supported them.
- Availability and accommodation, which reflects the geographical location, distance, hours of opening and the predictability of transport services to reach the screening centre.
- Affordability, which reflects whether the screening services were affordable and what were the opportunity costs of undergoing screening, such as missing work, cost of transportation to the screening location and costs of additional tests if required.
- Appropriateness, which refers to the interpersonal and technical qualities of the staff and the screening centre, respectively, as well as the adequacy of labour and equipment to conduct screening, the coordination of care and continuity that the screening centre provided following the screening test.

We extended this 5A framework to make it 7A framework by adding two aspects as they provided an understanding of other important barriers to screening, namely:

- Awareness, which reflects the degree of knowledge about the disease and its risk factors, degree of knowledge about screening, types and recommended frequencies, degree of knowledge about where to get the screening done and misconceptions about screening services.
- Angst and fear, which refers to the angst that the patients have about the results of the screening test, fear of pain, fear of gossip and stigma of screening and the fear of the impact that screening may have on family and community relationships.

We systematically reviewed empirical studies on barriers to breast and cervical cancer screening that cover one of the above-mentioned aspects of access in low- and middle-income countries (as per the World Bank classification). We included all types of studies (quantitative, qualitative and mixed-methods). The relevant information from the publications was extracted and classified as themes based on our 7A framework presented earlier.

Although this topic has been addressed in systematic literature reviews (Islam *et al.*, 2017), it was not comprehensively analysed from the perspective of the 7A framework. The inquiry of the barriers to access using the 7A framework helps us to better understand which aspects of access determine the uptake of screening. Our review results can help policymakers to design effective policies for promoting and enabling breast and cervical cancer screening. Moreover, we add to the development of Levesque’s framework, which is recommended by Cu *et al.*, (2021). Specifically, the comprehensiveness of the framework needs to be confirmed in new applications in diverse settings, especially in low- and middle-income countries.

## Materials and methods

We performed a qualitative systematic literature review to understand the evidence related to barriers to breast and cervical cancer screening in low- and middle-income countries. The review was based on PRISMA-S, an extension to the PRISMA Statement for Reporting Literature Searches in Systematic Reviews (Rethlefsen *et al.*, 2021). The available evidence was extracted and analysed through the 7A framework mentioned earlier. The methodology helped us gain in-depth insights into the persisting barriers to breast and cervical cancer screening in low- and middle-income countries through a logical structure and helped us understand the gaps in research. The study was registered on the International Prospective Register of Systematic Reviews, better known as PROSPERO, with registration number: CRD42020197720.

### Data source and search strategy

A systematic approach was followed to select the relevant publications. We performed a systematic literature search using PubMed, Mendeley and Google Scholar in September 2020 to retrieve all English language studies that contained information on breast and cervical cancer screening in low- and middle-income countries. The list of low- and middle-income countries was taken from the listing of low-income economies, lower-middle-income economies and upper-middle-income economies from the World Bank website (World Bank, 2021). The main keywords and their synonyms were used in different sequences and combinations with ‘OR’ and ‘AND’ operators to form various combinations of keyword chains. Finally, the chain with the least number of irrelevant publications was chosen. The final keywords chain used in the systematic literature search in September 2020 in PubMed with titles/abstracts filter is detailed in Supplementary Appendix 1.

### Inclusion criteria

The PICOS method was applied to select the publications: (1) population: women, their husbands, health workers, administrators, policymakers and religious leaders; (2) intervention: breast screening—self-breast examination and mammogram or mammography; cervical screening—Pap smear or Papanicolaou test and visual inspection with acetic acid; surgical procedures to remove cervical precancers such as Loop electrosurgical excision procedure and cryotherapy; (3) comparator: this was not a specific inclusion criterion since studies with and without a comparator group were included; (4) outcomes: barriers to access classified under the 7A framework of approachability, acceptability, availability and accommodation, affordability, appropriateness, awareness, as well as angst and fear; (5) study design: peer-reviewed publications to ensure the quality of research; only publications based on research on low- and middle-income countries; only articles published between January 2016 and May 2021 and only English language publications.

### Exclusion criteria

The same PICOS method was used to exclude the publications with the following characteristics: (1) population: studies on population groups in high-income economies and studies on migrants from low-income and low-and-middle-income economies to high-income economies; (2) intervention: unproven methods of breast and cervical screenings such as thermal screening; (3) comparator: this aspect did not provide exclusion criteria; (4) outcomes: no barriers to access reported; (5) studies: editorials, letters and personal views and publications of languages other than English.

### Selection process

**Figure 1.**
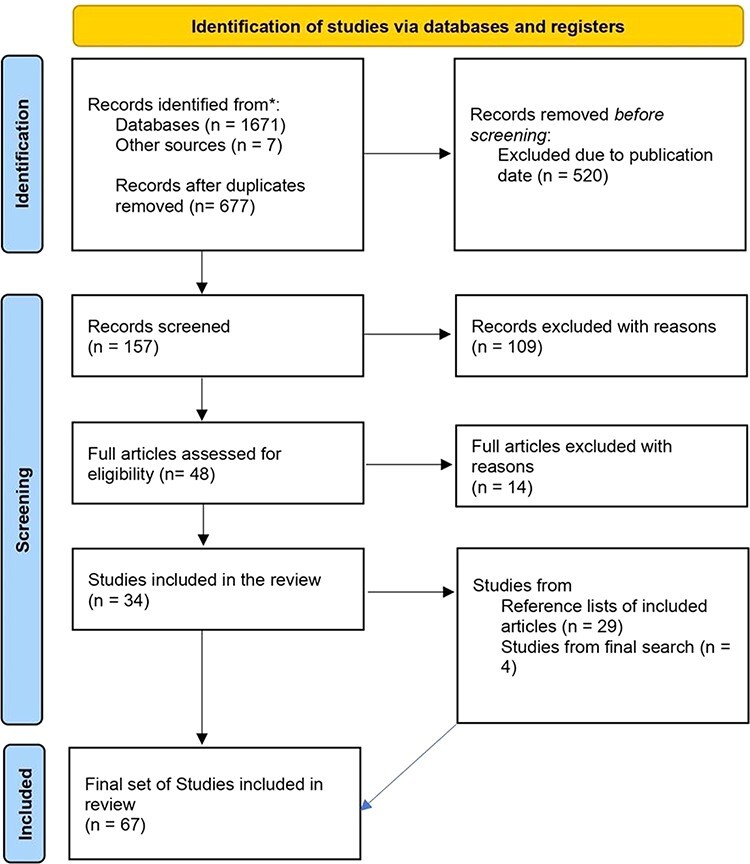
PRISMA 2020 flow diagram of the database search for relevant articles (Page *et al.*, 2021).

Figure 1 shows the flowchart diagram as per PRISMA 2020 of the database search for relevant articles. The selection of the publication followed three steps. First, the titles and abstracts were screened based on the above inclusion and exclusion criteria. This step was checked by F.v.M involved in the review to verify the selection. Second, publications provisionally selected on the first screening step were included in the second screening step based on full text. All authors received the papers excluded based on full text and were asked to comment on the exclusion. Finally, on the third screening step, the references in the selected literature were further checked for additional publications that might have been missed during the database search using the same inclusion criteria. Any points that needed clarity during the selection process were discussed with all researchers involved in the review.

### Data extraction

The analysis applied the method of directed qualitative content analysis. From the selected publications, information related to the barriers to breast and cervical cancer screening was extracted under the different aspects of access in the 7A framework: approachability, acceptability, availability and accommodation, affordability, appropriateness, awareness and angst and fear. Each of the seven aspects had different dimensions, which were numerically coded, and all key findings were summarized in tables and explained narratively.

### Quality assessment

The quality of the publications was assessed using the standard evaluation checklists such as Critical Appraisal Skills Programme (CASP) (for qualitative publications) and Effective Public Health Practice Project (EPHPP) (for quantitative publications). The qualitative study checklist had 10 questions with answer options of ‘Yes’, ‘Can’t tell’ and ‘No’. For each question that confirmed the fulfilment of a given quality criterion, the study got a score of 10 points. If the study was ambiguous regarding that criterion or did not meet that criterion, it received 5 or 0 points for that criterion, respectively. Overall, a study could get a maximum score of 100 points. The quantitative study checklist had eight components. Some components had subcomponents, while others did not. Each component had a grading of strong, moderate and weak. Each publication was graded for the eight components. Again, a study could receive a maximum overall score of 100 points, where each component had a grading of 12.5 points. A publication that received a strong grading scored 12.5 points, a publication that received a moderate grading scored 6.25 points and a publication that received a weak grading scored zero points. For mixed-methods studies, both the CASP and EPHPP checklists were applied, and therefore the maximum score of 200 points was used. For both checklists, there was an emphasis on the integrity of the sources, the validity of data collection and the relationship between the findings and the research question. Other criteria included the aim of the study, the clarity of the research question and whether the study design was suitable to answer the research question. We also checked the quality of our systematic review based on the PRISMA-S checklist (see Supplementary Appendix 2).

## Results

The literature searches on 1 September 2020 yielded 453 articles in PubMed Central, 558 articles in Mendeley and 660 articles in Google Scholar: thus, 1671 articles in total. In addition, eight articles were identified from journals in India. After duplicate articles were removed, 677 articles remained. These were checked for relevance and whether they fit the inclusion criteria. Only 157 articles fit the timeline between 1 January 2016 and 31 August 2020. Given the inclusion criteria, 109 articles were off-topic and were excluded. The second level of screening was conducted, where 48 articles were assessed for eligibility. After scrutiny, 14 articles had to be eliminated because they did not meet the relevancy criteria, and 34 articles met the inclusion criteria and were selected for the literature review. Out of the 34 articles, 17 articles had adopted qualitative analysis, 14 articles had used quantitative analysis and three articles had used mixed-methods analysis. The 34 articles that were selected for the literature review were checked for reference articles, which might not have appeared in the first-level search in the three databases. This exercise yielded 29 articles that were added to the literature review. A second search was conducted on 1 June 2021 using PubMed, Google Scholar and Mendeley to see if any new articles were published after 1 September 2020 that met the inclusion criteria. The second search yielded four additional articles.

A detailed description of the articles can be found in Supplementary Appendix 3. In the next section, the key characteristics of the articles are presented, followed by a narrative description of their findings on barriers to breast and cervical cancer screening in low- and middle-income countries regarding approachability, acceptability, availability and accommodation, affordability, appropriateness, awareness, as well as angst and fear.

### General description of the selected articles and quality assessment

The overall characteristics of the articles included in the review are presented in Table 1. The table shows that the publication of most articles (91%) was evenly distributed across the study period. In total, 69% of the studies originated from African countries, and 25% originated from Asian countries (mainly India and Nepal). Most of the studies took place in health-care organization settings or urban and rural community households. In total, 84% of the articles were solely focused on cervical cancer screening. Only 6% of the articles were solely focused on breast cancer screening. The objective in 53% of the publications focused on barriers to cervical cancer screening and/or treatment. Furthermore, 21% of the publications looked at motivators for cervical cancer screening. Of all studies reviewed, 55% were quantitative and 36% were qualitative studies. A mere 9% of the studies used a mixed-methods approach.

**Table 1.** Overall characteristics of the studies reported in the 67 articles included in the review

Characteristic of the publication	Number of publications (%)	Publication reference number in Appendix 3
Year of publication		
2016	14 (21)	5, 9, 12, 21, 39, 40, 41, 42, 47, 48, 53, 54, 55, 56
2017	14 (21)	3, 14, 18, 23, 24, 34, 35, 38, 43, 45, 57, 58, 59, 67
2018	16 (24)	1, 4, 10, 13, 19, 26, 28, 33, 36, 44, 46, 49, 50, 51, 52, 64
2019	17 (25)	2, 8, 11, 17, 20, 25, 27, 30, 31, 32, 37, 60, 61, 62, 63, 65, 66
2020 and 2021 (till 1 June 2021)	6 (9)	6, 7, 15, 16, 22, 29
Origin of the study		
Africa—Botswana, Burkina Faso, Cameroon, Ethiopia, Ghana, Kenya, Malawi, Nigeria, South Africa, Tanzania, Uganda and Zambia	46 (69)	3, 4, 5, 6, 7, 8, 10, 11, 13, 14, 16, 19, 20, 21, 23, 26, 28, 29, 33, 34, 38, 39, 40, 41, 42, 43, 44, 45, 46, 47, 48, 49, 50, 51, 54, 56, 57, 58, 60, 61, 62, 63, 64, 65, 66, 67
Asia—China, India, Iraq, Jordan, Nepal and Turkey	17 (25)	2, 9, 12, 15, 18, 22, 25, 27, 30, 32, 35, 36, 37, 52, 53, 55, 59
North and South America—Guatemala, Haiti, Honduras and Peru	4 (6)	1, 17, 24, 31
Study setting		
HIV clinic	8 (12)	2, 3, 11, 13, 26, 37, 49, 62
Gynaecological clinics	13 (19.5)	1, 7, 8, 9, 20, 35, 36, 43, 44, 45, 46, 48, 51
Clinics, hospitals, Primary health centres	7 (10.5)	18, 21, 25, 27, 29, 61, 63
Rural community households	17 (25)	5, 6, 10, 12, 17, 23, 24, 30, 40, 50, 52, 55, 56, 57, 58, 59, 64
Urban community households	12 (18)	22, 28, 32, 38, 39, 41, 42, 47, 53, 54, 65, 66
Urban and rural community households	7 (10.5)	4, 14, 16, 19, 33, 34, 60
Schools and churches	2 (3)	31, 67
Non-governmental organisations	1 (1.5)	15
Type of screening studied		
Cervical cancer screening	56 (84)	1-17, 20-21, 23-27, 29, 35-52, 54, 56-67
Breast cancer screening	4 (6)	19, 28, 34, 53
Both breast and cervical cancer screening	7 (10)	18, 22, 30-33, 55
Study objective		
Barriers to cervical cancer screening and treatment	17 (25)	1, 6, 8, 9, 10, 12, 21, 25, 38, 39, 40, 41, 42, 43, 44, 48, 56
Barriers and facilitators (motivators) for cervical cancer screening and treatment	14 (21)	3, 4, 7, 11, 14, 15, 27, 47, 52, 58, 60, 61, 63, 67
Knowledge-attitude-practices, barriers and facilitators for cervical cancer screening among women living with HIV	5 (7)	2, 13, 26, 49, 62
Knowledge-attitude-practices, and barriers towards cervical (cancer) screening	19 (28)	5, 16, 17, 20, 23, 24, 29, 35, 36, 45, 46, 50, 51, 54, 57, 59, 64, 65, 66
Knowledge-attitude-practices, and barriers towards breast (cancer) screening	4 (6)	19, 28, 34, 53
Knowledge-attitude-practices, and barriers to breast and cervical cancer screening	6 (9)	22, 30, 31, 32, 33, 55
Stigma (including HIV stigma) towards cervical or breast cancer or both	2 (3)	18, 37
Study design		
Mixed-methods (facility-based cross-sectional, pre- and post-assessment)	6 (9)	1, 20, 30, 31, 49, 62
Quantitative (cross-sectional, community-based cross-sectional, prospective facility-based, case–control, correlational cross-sectional, pre- and post-assessment, etc.)	38 (57)	3, 4, 6, 7, 8, 9, 15, 16, 17, 19, 24, 33, 34, 35, 36, 38, 39, 40, 42, 43, 44, 45, 46, 47, 48, 50, 51, 52, 54, 55, 56, 57, 58, 59, 64, 65, 66, 67
Qualitative (exploratory, descriptive case study, phenomenological, framework, cluster-randomized trial, etc.)	23 (34)	2, 5, 10, 11, 12, 13, 14, 18, 21, 22, 23, 25, 26, 27, 28, 29, 32, 37, 41, 53, 60, 61, 63

Table 2 provides a summary of the methods of data collection and analyses used in the 67 articles. In total, 43% of the studies focused on women living in either rural or urban communities. Additionally, 21% of the studies related to women visiting gynaecological or antenatal clinics, and 24% of the studies involved women along with their spouses and health workers and key influencers. The sample size of the studies was evenly distributed across the frequencies; in particular, 61% of the studies had between 101 and 1000 respondents. The data for nearly 40% of the articles were collected in the years 2015 and 2016. Table 2 also shows that 46% of the articles used interviewer-administered questionnaires. Overall, 45% of the articles employed a quantitative, descriptive, inferential analysis, while 59% of the articles analysed the data quantitatively and 24% of the articles employed a qualitative, thematic analysis.

**Table 2.** Methods of data collection and analysis used in the 67 articles reviewed

Characteristic of the publication	Number of publications (%)	Publication reference number in Appendix 3
Targeted population		
Women living with HIV	8 (12)	2, 3, 11, 13, 26, 37, 49, 62
Women visiting gynaecological and antenatal clinics	14 (21)	1, 7, 8, 9, 20, 27, 35, 36, 43, 44, 45, 46, 48, 51
Women living in the community (both rural and urban)	29 (43)	4, 5, 6, 12, 15, 16, 17, 19, 21, 24, 33, 34, 38, 39, 40, 41, 42, 47, 50, 52, 54, 55, 56, 57, 58, 59, 64, 66, 67
Other groups: women, husbands, local religious leaders, health workers, administrators, policymakers and volunteers	16 (24)	10, 14, 18, 22, 23, 25, 28, 29, 30, 31, 32, 53, 60, 61, 63, 65
Sample size		
Up to 50 respondents	14 (21)	2, 5, 10, 11, 12, 21, 22, 25, 26, 28, 32, 37, 41, 63
51–100 respondents	5 (7)	8, 14, 27, 29, 40
101–250 respondents	12 (18)	6, 9, 17, 18, 23, 24, 36, 38, 39, 46, 53, 59
251–500 respondents	16 (24)	3, 13, 15, 31, 35, 43, 45, 47, 48, 50, 51, 61, 62, 64, 66, 67
501–1000 respondents	13 (19)	1, 4, 7, 20, 33, 49, 54, 55, 56, 57, 58, 60, 65
≥1001 respondents	7 (10)	16, 19, 30, 34, 42, 44, 52
Year of data collection		
2012	2 (3)	3, 11
2013	2 (3)	5, 15
2014	7 (10)	14, 21, 24, 48, 53, 55, 59
2015	14 (21)	2, 7, 9, 20, 28, 31, 33, 37, 41, 42, 47, 52, 54, 62
2016	12 (18)	10, 13, 19, 26, 27, 34, 43, 46, 49, 61, 64, 65
2017	7 (10)	4, 25, 30, 32, 35, 50, 51
2018	2 (3)	16, 44
2019	2 (3)	6, 22
Multiple years	5 (6)	1, 12, 17, 18, 29
Not mentioned	14 (21)	8, 23, 36, 38, 39, 40, 45, 56, 57, 58, 60, 63, 66, 67
Method of data collection		
Interviewer-administered questionnaire	31 (46)	3, 4, 9, 15, 16, 19, 33, 34, 36, 38, 39, 40, 42, 43, 44, 45, 47, 48, 49, 50, 51, 52, 54, 55, 56, 57, 58, 59, 64, 65, 66
Self-administered questionnaire	6 (9)	6, 7, 8, 35, 46, 67
Focus group discussion	6 (9)	5, 11, 14, 21, 27, 53
In-depth interviews	11 (16)	2, 12, 13, 22, 26, 29, 32, 37, 41, 60, 61
Population-based survey	2 (3)	17, 24
Multiple data collection methods	11 (16)	1, 10, 18, 20, 23, 25, 28, 30, 31, 62, 63
Method of data analysis		
Quantitative—descriptive analysis	5 (7.5)	7, 17, 36, 40, 66
Quantitative—inferential analysis	4 (6)	42, 52, 55, 58
Quantitative—descriptive and inferential analysis	30 (45)	3, 4, 6, 8, 9, 15, 16, 19, 20, 24, 33, 34, 35, 38, 39, 43, 44, 45, 46, 47, 48, 50, 51, 54, 56, 57, 59, 64, 65, 67
Qualitative—content analysis	7 (10)	2, 5, 12, 13, 21, 23, 27
Qualitative—thematic analysis	16 (24)	10, 11, 14, 18, 22, 25, 26, 28, 29, 32, 37, 41, 53, 60, 61, 63
Mixed-methods	5 (7.5)	1, 30, 31, 49, 62

In Supplementary Appendix 4.1, the ranking of the barriers for each study has been mentioned, along with the number of participants who cited them wherever available. In quantitative studies, the data for the number of participants who cited a barrier as critical or important has been gleaned from each study to rank the barriers within each study. In qualitative studies, the barriers were ranked according to the weight given by the authors and the participants. Subsequently, we calculated an indicator of how frequently a barrier was most important when studied (the number of studies in which a barrier got ranked first divided by the number of studies in which the barrier was mentioned). We used that indicator to identify the most important barrier in a given region, setting, focus and stakeholders. The ranking outcomes for both types of studies are listed in Supplementary Appendices 4.2, 4.3, 4.4 and 4.5.

Among the seven barriers across 67 studies, awareness was the most significant barrier overall. However, there were differences in rankings between regions. In the 27 studies focused on East Africa, which were from Ethiopia, Tanzania, Somalia, Malawi and Uganda, Acceptability was frequently ranked as the most significant factor (in 55% of those that mentioned it) followed by awareness (in 40% of the studies that mentioned it). Whereas there were 16 studies from West African countries of Ghana, Nigeria, Cameroon and Burkina Faso, awareness was ranked as the predominant theme in 63% of the papers, followed by approachability (27%—lack of recommendation by a doctor or health practitioner) and acceptability (20%—restricted agency). Both were ranked as the main theme in three studies each. In India, there were 10 studies, where acceptability was ranked as the predominant theme in India (in 67% of the studies mentioning it), followed by awareness which was ranked first in 33% of the studies mentioning it. There were three studies each from the Middle East, Central America and Southern countries of Africa. Awareness was a common factor in all the above three locations. While restricted agency and hesitancy to be screened by male doctors were mentioned as an important factor in African countries of West and East Africa, it was not a factor in Asia or the Indian subcontinent. The barrier that was ranked as the second most important in East Africa was awareness cited in 40% of the studies followed by availability in 13% of the studies that mentioned it. In West Africa, 27% of the studies revealed that approachability was ranked as the second most important barrier, followed by acceptability with 20%. In India, awareness was ranked second, with 33% of the participants considering it to be significant, followed by affordability with 25%.

In 21 studies that were based solely in rural communities, awareness was the most important barrier, followed by acceptability. In the 34 studies which were done in an urban setting, awareness and acceptability were ranked equally as most significant by 42% of the respondents, followed by approachability. In total, 50% of the studies which were based in combined urban and rural settings had affordability ranked as the main barrier, followed by awareness (43%) and acceptability (33%).

There were only four studies that related exclusively to breast cancer, seven studies that dealt with both breast and cervical cancers and 56 studies that were concerned solely with cervical cancer. In the four studies which were entirely related to breast cancer, two studies ranked acceptability as the most important barrier. Approachability and awareness were ranked as the second and third most important barriers, respectively. The participants in the seven studies which dealt with both breast and cervical screening ranked acceptability first with 50% followed by angst (40%) and acceptability (33%). There were 56 studies that were concerned wholly with cervical cancer. In total, 45% of the studies in which awareness was mentioned ranked awareness as the most important barrier, acceptability was the second most important barrier (37%) followed by approachability (22%).

In studies where women were the only participants (51 studies out of 67), awareness was ranked as the most important dimension by 46% of the studies that mentioned it, lack of recommendation was cited as the second most significant by 32% of the respondents and acceptability factors such as restricted agency, embarrassment or the need for a female doctor and arranging childcare were ranked third in 29% of the studies. In studies where other stakeholders such as spouses, health workers and community leaders were also part of the study (16 studies out of 67), acceptability was found to be the most important finding in 69% of the studies, followed by awareness with 57% of the studies that mentioned it ranked it second.

The studies were appraised for internal/external validity and reliability based on the CASP appraisal checklist for qualitative studies and the EPHPP appraisal checklist for quantitative studies. Four qualitative studies were found to be of high quality, while the rest were of a medium level of quality. Two qualitative studies scored poorly on the checklist due to poor recruitment of candidates or insufficient information. All quantitative studies were found to be of a medium level of quality except one. In some of the studies, there was no information about research questions, the analysis process was not discussed and potential biases were not mentioned. Articles reporting on mixed-methods studies were appraised stepwise, and the qualitative and quantitative parts were assessed separately. Overall, validity and research design in these studies were poorly addressed, and thus, all of these studies were found to be of a medium level of quality.

### Approachability as a barrier to breast and cervical cancer screening

Table 3 presents the key findings on approachability to screening. The identification of screening services, how they can be reached and the impact on the lives of the women who seek them were important factors in understanding the barriers to screening. The quantitative studies highlighted that the lack of promotion of the need for cancer screening was the essential barrier to approachability, whereas the qualitative studies mostly enunciated the barriers related to the professional values of the service providers, such as the physicians not arriving at the screening facility on time and mismanagement of the appointment system leading to long queues. The lack of trust in the competence of the service providers also acted as a major negative factor.

**Table 3.** Approachability barriers reported in the 67 articles reviewed

Characteristic of the publication	Number of publications (%)	Publication reference number in Appendix 3
Outreach		
Not promoted/recommended by provider/doctor	12 (18)	3, 4, 7, 12, 15, 20, 34, 36, 40, 46, 48, 49
Only self-sampling available—patients prefer a health-care provider to collect the sample	1 (1)	21
Competing health-care burden—not prioritized by the health-care providers, service not provided	2 (3)	28, 64
Information		
Information not provided on screening	12 (18)	3, 6, 8, 10, 11, 12, 26, 30, 32, 36, 40, 45
Professional values		
Physicians not on time or unavailable or not receptive/inadequate counselling	3 (4)	10, 22, 63
Waiting times—long queues for screening/follow-up	8 (12)	3, 6, 10, 26, 29, 33, 56, 62
Trust and expectations		
Lack of trust in health-care providers’ competence based on previous experiences of relatives/friends or personal experience	10 (15)	1, 2, 12, 23, 27, 28, 40, 48, 49, 62
Distrust in medical workers over lack of confidentiality	3 (4)	18, 21, 37
Distrust in the health system—government hospitals/private hospitals	5 (7)	21, 22, 23, 49, 53
Discrimination/stigma by health workers due to religion or otherwise	3 (4)	21, 31, 37
Transparency/policies		
Patients felt health-care personnel had more power/lack of clear screening programmes	3 (4)	32, 40, 62

About 18% of the quantitative studies reviewed showed that a lack of promotion by the health-care provider was the most important barrier to breast and cervical cancer screening. This could be categorized into two types. First, there was no information provided by the health-care provider (physicians and nurses) about breast and/or cervical cancer. In one study from Tanzania (Koneru *et al.*, 2017), two-thirds of unscreened women reported that no one at the clinic had told them about cervical cancer. In another study, only 12.16% received information about cervical cancer from community health providers and only 6.76% from community health workers (Morris, 2016). Second, the physicians and nurses did not actively recommend screening. In a Nigerian study (Okunowo and Smith-Okonu, 2020), 42% of the women said that they would have undergone screening if it were recommended by a physician or a nurse. Only 37.7% of the women had been recommended breast screening by health practitioners (Olasehinde *et al.*, 2017). In a study from northern India, none of the women got information from an accredited social health activist worker, dai (traditional birth attendants who carry out deliveries of babies in the homes of villagers in northern India) or any other paramedical worker in their area. Overall, 37% of the women believed that if the Pap test was required, then their physician would prescribe it to them (Prateek *et al.*, 2018). Women who were encouraged to have screening by a health-care worker for medical reasons (advice from health-care professionals or symptoms) were twice more likely to get screening than those encouraged by relatives or friends’ advice (Compaore *et al.*, 2016).

The participants of the qualitative studies also said that the health workers did not explain why screening was needed, what benefits it had and when it had to be performed (Rasul *et al.*, 2016). Health-care workers might instruct women to have a cervical smear without explaining why this was necessary (Matenge and Mash, 2018). No sustained effort was made to create awareness about the clinic of the non-communicable disease (Mahajan *et al.*, 2019).

In total, 12% of the studies reviewed revealed that long waiting times in the screening centre put them off from seeking screening services. Physicians were not on time and extended hospital waiting time resulted in time loss for the market and business (Onyenwenyi and Mchunu, 2018). The lack of transparency in the appointment system, such as long queues and waiting times, inconsistencies in-clinic procedures and some procedures requiring appointments and others not requiring them (Matenge *et al.*, 2018), made the patients feel that the system was unfair. One quantitative Nigerian study also revealed that although the screening facilities were available at a comfortable distance, people were put off by the challenges of obtaining a screening appointment (Olasehinde *et al.*, 2019).

The qualitative studies revealed how discrimination by health-care providers led to distrust in the health system. Discrimination by health-care workers in health-care settings due to their health status or their religion was an uncommon but essential barrier. Discrimination against patients living with human immunodeficiency virus (HIV) hampered their access to cervical cancer screening (Gordon *et al.*, 2019). This extended to religion, where Muslim women felt discriminated against at health-care facilities because their mode of dressing readily identified their faith (Modibbo *et al.*, 2016). In addition, involuntary disclosure of HIV status by the health-care team discouraged women living with HIV from seeking cervical cancer screening services (Gordon *et al.*, 2019). This lack of trust in medical workers over their abuse of confidentiality (Nyblade *et al.*, 2017) also extended to the distrust over the genuineness of the service providers, with patients feeling that the screening services was one other way for hospitals to ‘snag money out of patients’ (Dey *et al.*, 2016).

### Acceptability as a barrier to breast and cervical cancer screening

Table 4 presents the key findings on acceptability to screening. Both the quantitative and qualitative studies revealed that women preferred to be examined by a female physician and women did not feel that they needed screening. In addition, the qualitative studies also divulged that the women needed the permission of their spouses or elders to undergo screening.

**Table 4.** Acceptability barriers reported in the 67 articles reviewed

Characteristic of the publication	Number of publications (%)	Publication reference number in Appendix 3
Personal factors		
Feel that screening is embarrassing even in front of female health workers	24 (36)	3, 4, 8, 12, 14, 15, 19, 21, 22, 23, 25, 26, 27, 29, 32, 33, 38, 41, 46, 49, 50, 53, 59, 62
Embarrassment—discomfort undressing in front of male physicians/nurses or discussing health issues with them	17 (25)	3, 5, 6, 8, 14, 15, 21, 22, 25, 27, 30, 33, 38, 46, 50, 53, 59
Prioritization/beliefs/procrastination		
Priorities—importance they give to cervical screening vs. other health issues or screenings like HIV	3 (4)	11, 26, 50
Will undergo screening only if symptoms appear/exist	12 (18)	11, 12, 16, 20, 22, 27, 28, 41, 49, 56, 58, 67
Feel that they are not susceptible (not at risk) to cervical cancer	17 (25)	6, 7, 9, 13, 21, 33, 36, 38, 41, 42, 44, 46, 47, 49, 56, 66, 67
There is no need to screen as it is not beneficial	12 (18)	9, 14, 16, 24, 28, 34, 36, 41, 44, 58, 64, 67
Lack of faith in modern medicine/belief in traditional medicine	3 (4)	10, 30, 63
Laziness/apathy/negligence delay	3 (4)	28, 53, 64
Autonomy		
Approval/moral support of husband needed for screening	22 (33)	1, 2, 4, 8, 10, 14, 15, 21, 22, 25, 27, 29, 30, 43, 46, 47, 49, 53, 60, 61, 63, 65
Lack of support (discouraged) or participation from relatives and friends (females)	8 (12)	1, 2, 7, 22, 25, 41, 53, 65
Lack of support (abstinence/condoms) from husband during recovery after screening/treatment	2 (3)	5, 61
Lack of financial support from husband for screening	3 (4)	40, 61, 63

From the quantitative papers analysed, participants from a study in Tanzania (Koneru *et al.*, 2017) said that they would prefer to be examined by a female physician and that they would feel more comfortable with a female nurse in the room than if they were being examined by a male physician, while another study from Ghana (Ampofo *et al.*, 2020) reported they did not like male health personnel offering screening services at all. Reasons for refusing to see a male physician were embarrassment, shame, over-exposing body parts, religious beliefs, husband’s disapproval and the inability to share feelings (Al-Amro *et al.*, 2020). In rare cases, women did not undergo screening because they were uncertain on whether their religion allows or does not allow cervical cancer services (Morris, 2016), and they were embarrassed to ask. The second most important reason for the lack of participation in screening by women with relation to acceptance was perceived non-necessity. This manifested in two ways. Women felt that they were not susceptible to cancer (Chaka *et al.*, 2018). This low-risk perception was the most common reason for not participating in screening activities among respondents who had never been screened before (Idowu *et al.*, 2016). Another common reason that women gave was that screening is not beneficial (Pryor *et al.*, 2017). Also, 18% of the respondents in a Malawian study were too lazy to go for screening (Maree and Kampinda-Banda, 2020).

The qualitative studies showed that the cultural norms of modesty were reported in almost all studies. Muslim communities within these countries were more emphatic about this barrier (Modibbo *et al.*, 2016). However, this was not unique just to Muslim societies. A Chinese study (Yang *et al.*, 2019) also discussed cultural barriers, including reticence for intimate examinations and reluctance to remove clothing or allow genital examinations, especially being exposed in front of non-family members. Women felt that screening has no value to them (Ilaboya *et al.*, 2018). There was a lack of understanding of the need to attend the screening (Gebru *et al.*, 2016). There was a strong influence of close contacts (family and friends) on screening decisions in 12% of the studies. If close contacts were not willing to participate, this reduced women’s acceptance of screening (Yang *et al.*, 2019). In addition, women’s perceived approval by their partners, family and friends influenced their screening practices. If female relatives and friends discouraged them from participating, it acted as a strong barrier. Family support, such as looking after the kids while the mother undergoes screening, acted as an enabler. In total, 33% of the studies showed that women needed spousal permission to undergo screening. Women needed their husband’s approval for finances because, most of the time, husbands gave money (Manga *et al.*, 2019). In some cases, their partners did not take actions supportive of their attempts to seek treatment, such as encouragement, childcare or transport money. The spouses were sometimes unwilling to provide funds for transportation, or women felt unable to disclose their need for transportation money due to distrust (Adewumi *et al.*, 2019). An Indian study showed that women needed permission from their husbands or fathers-in-law to visit the physician. They had to be accompanied by husbands or other males (Mahajan *et al.*, 2019). Another study from Nigeria revealed that women could not undergo screening as there was no consent from husbands, as they did not want their wives to be seen by other males (Onyenwenyi and Mchunu, 2018). Participants from two human papilloma virus (HPV)-based cervical screening programmes in Kenya were worried that partners would not believe the instructions from health professionals, such as abstinence or wearing condoms during sex (Adewumi *et al.*, 2019). This was partly due to cultural beliefs and a lack of understanding from their spouses. Women who were considered lost to follow-up tended to cite their partners as barriers. A small percentage of studies showed that women did not have faith in modern medicine, and they preferred to consult the village faith healer rather than seek help from their husbands or other male family members (Mahajan *et al.*, 2019). They are told by the faith healers that they have been healed from precancerous lesions or that the prayers will protect them from getting any form of cancer (Manga *et al.*, 2019). Procrastination, negligence and apathy were important barriers in a few studies. Women would wait and watch for an automatic cure and tended to approach the health system only in critical health situations (Dey *et al.*, 2016). The mixed-methods studies showed that the women felt that they should undergo screening only if symptoms appeared or existed (Getachew *et al.*, 2019).

### Availability and accommodation as a barrier to breast and cervical cancer screening

Table 5 presents the key findings on availability to screening. On the availability of screening services for breast and cervical cancer, the articles reviewed suggest that most countries included in the review do not have screening facilities in rural areas, and travelling long distances or time taken to reach the screening centre is one of the most significant barriers.

**Table 5.** Availability and accommodation barriers reported in the 67 articles reviewed

Characteristic of the publication	Number of publications (%)	Publication reference number in Appendix 3
Geographic location		
Rural environment	9 (13)	10, 20, 22, 23, 27, 30, 48, 61, 63
Lack of screening clinics or mobile screening units	17 (25)	4, 5, 8, 12, 13, 14, 17, 19, 20, 23, 28, 33, 40, 49, 50, 61, 62
Hours of opening		
Inconvenience of clinic time/limited opening times	3 (4)	1, 8, 15
Appointment mechanisms		
Lack of clarity or inconsistency regarding screening appointments	2 (3)	19, 26
Transport		
Distance/travel to reach Screening	18 (27)	1, 3, 5, 14, 24, 26, 29, 30, 32, 40, 41, 44, 48, 56, 58, 61, 63, 67
Mobility		
Unpredictability of public transport or lack of it	7 (10)	14, 15, 26, 32, 41, 44, 63
Absence of road/path for vehicle	3 (4)	15, 32, 44
Accommodation		
Screening hospital/screening room not wheelchair friendly	1 (1)	32

The quantitative studies showed for example that, in Nigeria, even though the screening was available on most days, the timings were not suitable (Amos and Awolude, 2019) as the women could not leave their work and attend the screening. In most countries included in the review, the proportion of unscreened people increased proportionately with the increase in the time taken to reach the screening clinic (Koneru *et al.*, 2017). Women living in urban areas had higher odds of screening compared with those living in semi-urban areas, and women in semi-urban areas had greater odds of being screened compared with rural women (Compaore *et al.*, 2016).

The qualitative studies revealed the lack of screening facilities. For example, in Nigeria, screening facilities were not at all available in rural areas (Onyenwenyi and Mchunu, 2018). The autonomous region of Kurdistan bordering Iraq, Iran, Turkey and Syria has four provinces with only one cancer screening centre in each province (Rasul *et al.*, 2016). While, in some cases, there were no basic health facilities for long distances, countries like Ghana (Ebu, 2018), the state of Tamil Nadu in India (Mahalakshmi and Suresh, 2020) and a few places in Uganda (Ilaboya *et al.*, 2018) had good primary health-care centres promoted by the state, but since cancer screening is part of tertiary health care, the breast and cervical cancer screening tests were unavailable at these primary health-care centres. The unpredictability of public transport was a barrier in accessing breast and cervical cancer screening. If the distance to the screening centre was also very far, like in Botswana (Matenge *et al.*, 2018), it discouraged women from undergoing screening.

Some of the mountainous regions, deserts and dense forests are so remote that there is an absence of a path or road for the vehicle to traverse and transport people. This was highlighted by both qualitative and quantitative studies. Women from the villages located in the Amazon forests of Peru (Collins *et al.*, 2019) and the deserts in Ethiopia (Teame *et al.*, 2019) and villages located in the mountains of Honduras (Pryor *et al.*, 2017) and Nepal (Darj *et al.*, 2019) may have to travel up to 5 days to access primary health facilities or village health posts which do not have cancer screening facilities. Their only means of transport to the cancer screening facility was by walk. This was not, however, a problem in Guatemala. Even though free public screening clinics were available in rural areas (Austad *et al.*, 2018), they offered cancer screening intermittently and attending specific days was difficult.

### Affordability as a barrier to breast and cervical cancer screening

Table 6 presents the key findings on affordability to screening. The study participants argued that breast and/or cervical cancer screening involves high out-of-pocket expenditure and that the participants would be willing to undergo the screening if the screening was free of charge or the fees charged were lower. A mixed-methods study in Guatemala (Austad *et al.*, 2018) showed that women did not undergo follow-up screening tests with the recommended frequency because of the costs involved.

**Table 6.** Affordability barriers reported in the 67 articles reviewed

Characteristic of the publication	Number of publications (%)	Publication reference number in Appendix 3
Direct and indirect costs		
Lack of free-of-charge screening	10 (15)	1, 4, 13, 14, 15, 16, 21, 33, 49, 50
High out-of-pocket expenditure	22 (33)	6, 7, 8, 9, 10, 13, 14, 19, 20, 22, 23, 35, 39, 40, 43, 46, 47, 49, 56, 58, 59, 67
Cost of additional tests if found suspicious	4 (6)	23, 25, 27, 53
Cost of treatment if found cancerous	7 (10)	17, 23, 25, 27, 41, 53, 63
Cost of transport to screening location	6 (9)	5, 14, 23, 29, 60, 61
Lack of incentives for screening	2 (3)	14, 61
Opportunity costs		
Work commitments/missing work (deduction from pay)	8 (12)	1, 2, 10, 22, 25, 26, 40, 53
Arranging childcare and other family commitments	3 (4)	1, 2, 59
Lack of time or screening takes too much time	10 (15)	14, 17, 25, 27, 40, 46, 50, 56, 59, 64

The quantitative studies also showed that especially when they required money from their husbands to undergo screening, it was a non-starter (Morris, 2016). Willingness to undergo screening was largely influenced by family income. In certain cases, women could afford clinical breast examinations by a physician, but only one-fifth of those surveyed could afford mammography (Olasehinde *et al.*, 2019). Women from a study in Jordan (Al-Amro *et al.*, 2020) were not willing to pay for the screening at all, even when it was affordable. This was also said in a study from Tanzania (Weng *et al.*, 2020) and Kenya (Gatumo *et al.*, 2018). It is remarkable that women who had obtained tertiary education were less willing to pay for screening. In one study, women were not willing to undergo cervical screening simply because it took too long (Pandey and Karmacharya, 2017). Women interviewed in a study in Uganda (Ndejjo *et al.*, 2017) were hesitant to undergo screening because they were worried about the cost of additional tests if found to be suspicious.

Most qualitative studies from Africa revealed that since the test was not free of charge, even if the test was available, women could not afford it (Ebu, 2018) and did not undergo screening. The opportunity costs of undergoing screening, such as foregoing a day’s wages or the cost of arranging childcare or the cost of paying transportation to reach the screening clinic, were the second most cited barriers.

Women interviewed in a study in India (Dey *et al.*, 2016) said that going to the hospital resulted in absenteeism from work and, consequently, no payment. They were worried that the kids would have to sleep hungry. A study in Uganda (Hasahya *et al.*, 2016) mentioned that the cost of transportation was much more than the cost of screening. Another associated factor was the time involved in travelling long distances to reach the camp. Both the cost of transportation and the time involved, as shown in a study in Tanzania (Bateman *et al.*, 2019), discouraged women from undergoing screening even though the screening was free of charge. A study in Kenya (Lunsford *et al.*, 2017) showed that women were willing to undergo the screening if they were given incentives or if they were reimbursed by the state for transportation (Huchko *et al.*, 2019). Women who were employed complained that it was difficult to take time off work, as they would have deductions from their pay if they were absent to attend the clinic (Matenge *et al.*, 2018). The same sentiment was echoed in a study in China (Yang *et al.*, 2019), where women with fewer economic resources reported avoiding screening because they were worried they would not have the money for treatment if they were diagnosed with cancer. They noted that while screening is free of charge, treatment is not. The same study also discussed women not being able to allocate time for screening. The feeling that the screening was just a preliminary step and did not give the final answer brought concerns about incurring unexpected costs if additional investigations were necessary, and they discussed having to face impossibly high charges if surgery was needed (Darj *et al.*, 2019). Where the women were housewives, they had to depend on their male partners to pay for the screening, and the cost became prohibitive (Lunsford *et al.*, 2017).

### Appropriateness as a barrier to breast and cervical cancer screening

Table 7 presents the key findings on appropriateness to screening. Whereas qualitative studies showed that hostile attitudes or ill treatment by physicians and nurses made women not to seek screening at all, health workers not sharing the screening results were noted as a barrier in both qualitative and quantitative studies.

**Table 7.** Appropriateness barriers reported in the 67 articles reviewed

Characteristic of the publication	Number of publications (%)	Publication reference number in Appendix 3
Technical quality		
Not having all types of equipment/latest equipment needed for screening	4 (6)	26, 28, 29, 30
Dirty and unsterile facilities	2 (3)	12, 53
Interpersonal quality		
Hostile attitude/ill treatment by physicians or nurses	7 (10)	2, 8, 23, 29, 33, 49, 50
Health workers not explaining (significance) test results or screening or need for repetition	3 (4)	12, 26, 63
Health workers did not know how to assist a disabled person	1 (1)	32
Adequacy		
Shortage of manpower	6 (9)	5, 20, 23, 26, 30, 62
Equipment/screening material shortage (quantity shortage)	4 (6)	5, 23, 26, 40
Coordination and continuity		
Never received test results	4 (6)	1, 5, 24, 26
Received test results late	2 (3)	17, 26
Not received explanation/follow-up for abnormal findings	2 (3)	1, 63

In the quantitative studies, lack of empathy and patience in explaining the screening process (Chaka *et al.*, 2018) and lack of ability to understand the emotions of patients were mentioned as bad experiences along with the lack of continuity which included receiving the test results late and patients not being informed of any abnormal findings. For example, in Honduras (Pryor *et al.*, 2017), where more than one-third of the women who had undergone cervical cancer screening in the survey population did not know the results of their Pap smears. They perceived this to be one of the issues but not as much as the uncertain adequacy of manpower or equipment to perform screening.

The findings from the mixed-methods studies showed that no appropriate care was taken at the screening facility and there was an inadequacy of the proper knowledge, which was a major concern shown by a study from Ethiopia (Shiferaw *et al.*, 2018). The second biggest barrier related to the appropriateness of services from the mixed-methods studies reviewed was the shortage of manpower, as discussed in another study from Ethiopia (Getachew *et al.*, 2019).

In the findings from a quantitative study from Uganda (Ndejjo *et al.*, 2017) and a qualitative study from the subcontinent of India (Mahajan *et al.*, 2019), a common theme exists, which is a deficiency in the number and skills of the staff, lack of proper training to carry out screening and lack of screening infrastructure such as screening materials and the lack of equipment.

### Awareness as a barrier to breast and cervical cancer screening

Table 8 presents the key findings on awareness to screening. Awareness was found to be the most common barrier to breast and cervical cancer screening so much so that some quantitative studies have been completely dedicated to the topic of awareness, such as Mabelele *et al.*, (2018), Mitiku and Tefera (2016) and Bora *et al.*, (2016).

**Table 8.** Awareness barriers reported in the 67 articles reviewed

**Characteristic of the publication**	**Number of publications (%)**	**Publication reference number in Appendix 3**
Lack of knowledge about screening and disease		
Lack of knowledge about the disease and its risk factors	39 (58)	2, 3, 5, 6, 13, 14, 16, 17, 21, 23, 25, 26, 27, 28, 31, 33, 34, 35, 36, 39, 41, 42, 43, 44, 45, 46, 47, 48, 49, 50, 51, 52, 53, 54, 55, 57, 64, 65, 66
Lack of knowledge about the screening (its recommended frequency, urgency types, etc.)	41 (61)	2, 3, 7, 9, 10, 11, 12, 13, 14, 19, 20, 23, 24, 25, 26, 27, 31, 32, 33, 35, 36, 38, 39, 40, 41, 42, 43, 44, 45, 46, 48, 49, 52, 53, 54, 57, 60, 64, 65, 66, 67
Lack of knowledge about the accessibility		
Lack of knowledge on where to get the screening done or whether it is free	19 (28)	3, 4, 6, 15, 19, 21, 23, 32, 35, 40, 46, 47, 48, 49, 52, 56, 59, 66, 67
Misconceptions		
Incorrect understanding about screening and disease	20 (30)	1, 5, 6, 8, 10, 11, 15, 16, 21, 22, 23, 25, 26, 27, 30, 36, 39, 53, 57, 61
Superstitions/religious beliefs	11 (16)	12, 14, 15, 18, 21, 23, 29, 30, 38, 40, 66

In the quantitative studies, women’s knowledge of cervical cancer was generally inadequate and was persistently associated with education, family income and family cancer history (Weng *et al.*, 2020). In one study, only 21.4% knew that HPV is a risk factor for cervical cancer (Chaka *et al.*, 2018), and in other studies, 52.1% of the participants did not know that cervical screening is free, and 57.3% did not know that cervical cancer treatment is free (Koneru *et al.*, 2017). In another study (Ampofo *et al.*, 2020), 110 participants (55%) did not know about any health facility offering cervical cancer screening. In another study from Jordan, 34% of those surveyed did not know if screening services were available in their locality (Al-Amro *et al.*, 2020). In a study from Ife Central province in Nigeria, despite mammography service being available, 88.2% of the respondents were unaware of its existence (Olasehinde *et al.*, 2019). In another study (Koneru *et al.*, 2017), approximately half of the women did not know that HIV infection increased the risk of cervical cancer, and one-fifth of women did not know that a woman could have cervical cancer and not know it. In the same study, 90% knew that early detection and treatment of cervical lesions could prevent the development of cervical cancer, but only 9% had been screened—indicating a lack of connection between this general knowledge of cervical cancer and the need or urgency to be screened. Participants in a study from the Middle East responded that cervical cancer only happens to women who are above the age of 50 years (Al-Amro *et al.*, 2020).

When we looked at qualitative studies, none of the Muslim participants in a focus group discussion conducted in Southwestern Nigeria had ever heard about cervical cancer. Most did not understand the part of the body affected by it or where the cervix is located (Modibbo *et al.*, 2016). In one of the studies, women were not aware that cervical cancer was preventable, and they were also not aware that there is a treatment for cervical cancer precursor lesions (Matenge *et al.*, 2018). Overall, qualitative studies showed that the lack of knowledge about the screening (its recommended frequency, urgency types, etc.) was a barrier. In one of the studies, women believed that a Pap smear was only needed when women were old, married or symptomatic (Rasul *et al.*, 2016). A study (Lunsford *et al.*, 2017) reported that women lacked information about (1) cancer and cervical cancer awareness, (2) who gets cancer, (3) signs and symptoms of cancer and (4) the benefits of screening and what occurs during different screening procedures. The majority of them have not heard about any screening method (Gebru *et al.*, 2016). In another study, women had erroneous beliefs that cervical cancer is a disease that attacks the womb either through blood or sexual transmission or through genes (Bateman *et al.*, 2019). Women in one study said that only women who have had babies need to do cervical cancer screening. Furthermore, studies also showed that superstitions or religious beliefs acted as barriers to screening. In a study from Uganda (Hasahya *et al.*, 2016), women had a belief that HPV vaccinations were to prevent their daughters from having more than two children in the future. In a study in India, women felt that cancer is a punishment for misdeeds either in their ‘current or past’ life (Nyblade *et al.*, 2017). In another study in Nigeria, women who believed in wizardry among Christians mentioned that cervical cancer might be caused by charms deployed by men unhappy with their female sexual partners (Modibbo *et al.*, 2016). The same study showed that Muslim participants believed that their religion did not allow them to undergo screening.

Across the countries included in the review, there was a lack of awareness about the disease, screening, its availability and the correct understanding, which hindered the use of screening services.

A mixed-methods study from Haiti (Tillyard *et al.*, 2019) reported that only 12% of the participants knew what a test for cervical cancer would be. When asked about cervical cancer screening, women often had no concept of what the screening was for, what happened to a woman while she was being screened or why it was important to be screened. In total, 28% of the studies revealed that the patients did not have knowledge about where to get the screening done or whether it is free. In addition, 30% of the studies showed that the patients had misconceptions or incorrect understanding about the screening and disease. Participants from another mixed-methods study (Austad *et al.*, 2018) linked cervical cancer to the use of family planning methods or to have many kids.

### Angst and fear as a barrier to breast and cervical screening

Table 9 presents the key findings on angst to screening. Angst was a remarkable finding, and it was possible to distinguish two dimensions. The first dimension was acceptability, as it was a personal feeling. At the same time, the cause of that fear was mainly a lack of awareness.

**Table 9.** Angst and fear barriers reported in the 67 articles reviewed

Characteristic of the publication	Number of publications (%)	Publication reference number in Appendix 3
Fear of pain	20 (30)	3, 5, 8, 12, 16, 22, 26, 29, 33, 36, 39, 41, 42, 43, 46, 49, 50, 58, 59, 67
Fear of screening test results (cancer diagnosis)	27 (40)	2, 8, 10, 12, 14, 16, 18, 19, 20, 21, 22, 23, 25, 26, 27, 32, 33, 38, 39, 40, 46, 47, 48, 49, 56, 59, 67
Fear of gossip/stigma/shame—isolation from family/society—blamed for promiscuity, etc.	10 (15)	1, 11, 14, 22, 27, 36, 41, 47, 53, 60
Fear of disability and death (fatalistic belief)	6 (9)	11, 12, 28, 33, 41, 53
Fear of nosocomial infections	2 (3)	5, 21
Fear of screening/vaccination	12 (18)	4, 6, 11, 16, 17, 22, 24, 33, 41, 58, 63, 64
Fear of discovery that they have contracted cervical cancer from husbands during cervical cancer screening	1 (1)	5
Fear of being diagnosed with HIV (or other health issues) during cervical cancer screening	2 (3)	5, 53
Fear of speculum (belief that it causes infertility)	4 (6)	12, 14, 41, 63
Fear of transmission of cancer during screening	1 (1)	18
Fear that cancer screening/diagnosis will threaten the relationship with spouse/partner—lead husband to stray and violence	9 (13)	5, 11, 18, 31, 33, 50, 53, 59, 60
Fear of surgery and its consequences—loss of feminity and removal of breast	3 (4)	28, 40, 53
Fear of hospitals	1 (1)	28

Several quantitative studies revealed the different aspects of fear which acted as a barrier for women seeking breast and cervical cancer screening. Fear of the test itself was cited as an obstacle to some women, even if they appreciated the need for screening, as recorded in a study from Ghana (Ampofo *et al.*, 2020). In another study from Ethiopia, 74.9% responded that they did not take the screening services because of fear of painful test procedures (Bayu *et al.*, 2016) and being diagnosed with cancer meant dying shortly thereafter (Chaka *et al.*, 2018). Fifty-nine participants (56.7%) in another study indicated that they could not undergo the test due to fear of the results (Abdikarim *et al.*, 2017). Not only did women fear the screening due to a cancer diagnosis, but they were also afraid of the various societal ramifications and the way society treated women who underwent screening. Women did not want to undergo screening as they felt a sense of shame in explaining the test to their family members (Prateek *et al.*, 2018). Similarly, Chaka *et al.*, (2018) recorded that over a third (37.8%) responded that breast cancer would threaten a relationship with her husband, boyfriend or partner. A cervical cancer study in Kenya (Gatumo *et al.*, 2018) recorded 57% of the respondents saying that a cervical cancer diagnosis would threaten a relationship with a boyfriend, husband or partner. Women feared husbands tagging them as promiscuous (Idowu *et al.*, 2016).

Qualitative studies showed that a large proportion of both unscreened and screened women thought that the cervical examination was painful (Hasahya *et al.*, 2016), while several articles suggested that the fear of screening results was a barrier to undergoing screening. In one of the studies from India, more than 75% of the women who were interviewed feared the Pap smear and the subsequent results (Kung *et al.*, 2019). Fear of certain death from a cancer diagnosis led women to avoid the service (Gebru *et al.*, 2016). Women feared that the results might reveal a life-threatening or incurable disease which might lead to suffering and disability (Matenge *et al.*, 2018). In another study, women feared big hospitals, and they had the fear of the unknown in case of a diagnosis of breast cancer during breast screening (Ilaboya *et al.*, 2018) and the stigma and isolation from society (Bateman *et al.*, 2019). Fear of societal gossip was a major barrier, in two studies from Nepal (Darj *et al.*, 2019) and India (Mahalakshmi and Suresh, 2020). There were stigmatizing attitudes towards HPV, including an association with promiscuity, infidelity and HIV (Adewumi *et al.*, 2019). Fear of negative reactions from husbands was also seen. Women feared the reaction of their husbands if diagnosed with HIV during cervical cancer screening (Hasahya *et al.*, 2016). Few of the women remarked that cancer evoked secrecy; in some cases, it was associated with other stigmatized illnesses, such as a concern that a diagnosis would lead a husband to stray (Nyblade *et al.*, 2017) and blame for any cancer diagnosis (Dey *et al.*, 2016). Hesitation to inform male members present in the family was also found to be one of the reasons to avoid approaching physicians. Four studies revealed that the fear of speculum was a barrier to screening. It was perceived as a painful instrument (Gebru *et al.*, 2016). Women expressed discomfort with the speculum, and there existed a strong belief that it caused infertility (Rasul *et al.*, 2016). Women expressed that they would not be able to bear all the side effects of a cervical screening examination like the vagina discharge (Manga *et al.*, 2019). Two studies revealed that women had a fear of nosocomial infections. Women feared contracting other illnesses in the health service settings. They felt that the screening was done using unsterilized equipment (Modibbo *et al.*, 2016). One study revealed that women feared cancer transmission during screening (Nyblade *et al.*, 2017).

Two mixed-methods studies showed that women feared community-level gossip about the diagnosis and the ill effects of screening (Austad *et al.*, 2018). Women also feared violence from their husbands (Tillyard *et al.*, 2019).

## Discussion

This review found four key barriers to breast and cervical cancer screening in low- and middle-income countries: lack of knowledge about the disease and screening, embarrassment to undergo screening, lack of support or permission from husbands to undergo screening and lack of recommendation by the doctor. There were other barriers that were frequently pointed to in the studies: fear of screening test results, distance to the screening clinic and high cost of screening. Levesque’s framework of accessibility of health services was modified to overcome the challenge of categorizing certain study-specific parameters into the predefined dimensions of the model, and this contributed to the development of the model. There were variations in the rankings of the barriers according to the region, setting (rural or urban in nature), focus (breast screening or cervical screening) and stakeholders (only women participants or also other stakeholders). Acceptability factors were found to be the most important in countries of East Africa and India, whereas awareness was ranked as the most significant factor in West Africa. In countries of Asia, Southern Africa and the Middle East, approachability was the most important dimension. Availability was one of the important factors in East Africa but was not found to be important in the rest of the regions. Affordability was important in East Africa, India and Central America. East Africa and India were found to have similar rankings of barriers. While Islam *et al.*, (2017) found that lack of awareness along with lack of education and employment inhibits women from undergoing screening, our study found angst and fear of screening and screening test results and cultural issues such as lack of permission and support from husbands and embarrassment to be more prominent.

Overall across all regions and when classified according to study setting or the focus of the study (whether cervical or breast or both), awareness was ranked as the most important barrier. Women had heard of cervical cancer but were not aware of the causes, risk factors and symptoms of cancer, and there was a reluctance to accept that one is susceptible to cervical cancer (Hasahya *et al.*, 2016). Current evidence suggests that when women are aware of the causes and risk factors of cervical cancer and perceive themselves to be at risk, they are more likely to take up screening and measures to prevent the disease (Lyimo and Beran, 2012; Jia *et al.*, 2013; Morema *et al.*, 2014; Mukama *et al.*, 2017). Health systems in low-and-middle-income-countries need to focus on implementing evidence-based education strategies and community education programmes to increase the knowledge about breast and cervical cancer in society and improve adherence to screening. A systematic review, which analysed various educational interventions to improve the cervical screening behaviour of women, found that educational interventions based on health behaviour change theories, especially the health belief model, were the most effective interventions (Naz *et al.*, 2018). The current literature also finds that radio broadcast increases the knowledge related to the disease and screening tests among older and under-screened women (Perkins *et al.*, 2007). Policymakers could measure cancer literacy in a given population using evidence-based tests such as Assessment of Health Literacy in Cancer Screening (Han *et al.*, 2014), which are specific to cancer. This could help to gauge the efficacy of awareness campaigns and improve their effectiveness.

Nevertheless, it needs to be considered that socio-cultural factors could prevent women from accessing screening services even if there is awareness and screening is closely available. In this regard, another finding from our review was that the lack of spousal support was a major barrier to the uptake of breast and cervical cancer screening. Ghanaian men in a study (Williams and Amoateng, 2012) expressed willingness to support their spouses to undergo cervical screening if they had more information about cervical cancer and cervical cancer screening. Similarly, another study (Adegboyega *et al.*, 2019) done in the USA showed that sub-Saharan African immigrant men expressed willingness to support their partners for cervical cancer screening if they were given more awareness about it. Socio-cultural understanding of the region and location and involving the spouses by educating them might see an improvement in women undergoing screening with the support of their spouses. Also, we found that the barriers to the uptake of breast and cervical cancer screening in low- and middle-income countries include the embarrassment of undergoing screening from a male doctor or health worker. This problem is observed in high-income countries as well. A study of indigenous Maori women in New Zealand reported that 75% of women who did not attend cervical screening due to shyness or embarrassment were willing to do an HPV self-test and attend follow-up if they received a positive HPV test result (Adcock *et al.*, 2019). Socio-cultural factors specific to the context need to be considered for an effective screening intervention.

We found in the review that women hesitated to undergo screening unless it was recommended by a physician. It is known that a recommendation by a doctor or a general practitioner can act as a motivator to undergo screening (O’Connor *et al.*, 2014). To increase the ease of recommendation by the physician for cancer screening, the screening guidelines need to be simplified and universal and reduce inconsistencies between recommendations of different organizations, which are also supported by valid evidence and meet accepted norms (Curry *et al.*, 2003) so that doctors refer every patient who deserves it rather than only refer the low-hanging fruits which are easier and straightforward. The same book also goes on to add that increasing the likeness of the patient following physician recommendation is dependent on creating an accurate perception of the magnitude of risk from cancer.

Another important finding from the studies was that women did not attend screening because they feared screening test results. Due to the non-availability of suitable treatment in government hospitals or the inability to afford treatment in private hospitals (Onyenwenyi and Mchunu, 2018; Amos and Awolude, 2019), women believed that they would not be cured even if cancer is diagnosed through screening at an early stage (Olasehinde *et al.*, 2017; Chaka *et al.*, 2018). The barrier to treatment, in this case, became a barrier to screening also. Women could feel that rather than be found to have cancer during screening and not being able to afford subsequent treatment, it would be rational not to attend the screening at all. There was an underlying distrust in the health system regarding both government and private hospitals, which was reflected in the non-adherence to screening (Modibbo *et al.*, 2016; Mahalakshmi and Suresh, 2020). In low- and middle-income countries, the burden of health-care costs falls primarily on patients and there is little or no support from the state (Kankeu *et al.*, 2013). More commitments to resources towards cancer control and treatment are required so that countries can provide adequate access to cancer treatment. For example, the Government of India, through innovative financial models, has been able to fund part of the patients’ cancer treatment through the Ayushman Bharat Pradhan Mantri Jan Arogya Yojana scheme. Within 10 months of its inception, nearly a quarter-million patients had received (Kaur *et al.*, 2020) cancer treatment through this scheme. By linking value chains of cancer technologies between high-income countries to itself, Rwanda became the first low-income country in the world to roll out HPV vaccination to its preadolescent girl population in 2011. More than 93% of adolescent girls in the country were vaccinated through a very effective nationwide educational campaign and the use of cold-chain technologies (Li and Sullivan, 2018).

The availability of screening clinics and the distance to reach the screening clinic were recognized as major impediments to breast and screening in the studies reviewed. To overcome these barriers, various strategies could be employed. For example, the health department of the Philippines has used vans or buses as mobile cancer detection clinics where screen-and-treatment methods could be performed in remote areas as part of its breast cancer control programme to supplement the gaps in the country’s breast cancer screening endeavours (Ting *et al.*, 2020). In Peru, where accessibility to screening clinics is a problem (Collins *et al.*, 2019), they developed a model where they trained promoters for community outreach, professional midwives in clinical breast examination, doctors to perform fine-needle aspiration biopsy sampling with ultrasound to triage and patient navigators to ensure patients follow through with treatment (Bain *et al.*, 2018). The model was found to be effective and beneficial to the population of Peru, and the Peruvian health information system now has specific breast cancer detection categories.

The affordability of screening was established as an important inhibitor for cancer screening in our review study. As Banerjee and Duflo (2011) argue, poor people are often able to attract resources from their (family) network if the disease is fatal. However, measures need to be taken by policymakers to avoid the affordability problem. For example, a study in Sweden found that self-sampling for HPV testing increased participation and detection of cervical cancer at a lower cost than midwife-collected Pap smears in primary cervical screening (Aarnio *et al.*, 2020). Offering women a home-based self-sampling was therefore a more cost-effective alternative than clinic-based screening. A study from China informed that HPV self-sampling was not only beneficial to enhance women’s health awareness but also to promote the cervical cancer screening uptake rate. Women who were under-screened, including those who had never been screened, were more likely to prefer HPV self-sampling than those who had regular screening (Wong *et al.*, 2020). Women from an economically disadvantaged population who did not have jobs and had to depend on their husbands for the cost of screening and women who did menial jobs and could not afford to leave their work and attend screening due to loss of daily wages were most likely to be benefitted from a self-test that could be done at home (Basu *et al.*, 2006). The same study found benefits from inviting husbands and elderly members of the house to group counselling meetings on breast and cervical screening. A 20-year prospective cluster-randomized trial conducted in Mumbai indicates that clinical breast examination conducted every 2 years by primary health workers brought down the staging of breast cancer at diagnosis, a significant reduction in mortality in women aged ≥50 years and a non-significant reduction of 15% in breast cancer mortality overall (Mittra *et al*., 2021). The health workers who screened women during this trial clinical breast examination had passed 10th-grade education and could be trained to perform clinical breast examination within a minimal training period.

There is a correlation between educational attainment and knowledge of risk factors not only between low- and middle-income countries and high-income countries but also between the different ethnic, racial and immigrant groups within high-income countries (Schoueri-Mychasiw and McDonald, 2013; Akinlotan *et al.*, 2017). In high-income countries, like Australia and the USA, there is a lack of knowledge about the importance of screening (Azar *et al.*, 2022), but in low- and middle-income countries, there is a lack of knowledge about the disease itself. While awareness, availability and affordability were the most significant factors in low- and middle-income countries, acceptability factors such as prioritization (lack of time) and angst (fear of pain and discomfort) were prominent in high-income countries (Wilding *et al.*, 2020) while also being common with low- and middle-income countries.

### Study limitations

This study has some limitations that need to be acknowledged. Our review only included studies reported in English language publications. This indicates possible publication bias since relevant studies might have been reported in another language and/or not yet published. The extraction of data from the articles was done by one author only, which may have led to selection bias. The selection bias was, however, partly diminished by having another researcher screening the papers excluded based on title and abstract. In addition, the quality check of the studies was done by one author only, which may have resulted in evaluation bias. Moreover, quality checklists have been used to score quality and compare the studies, but we might have missed study-specific quality details. This bias was also partly reduced by discussing the unclear papers with all co-authors. Most of the studies originated from India and North, Central and East Africa. Only a few studies originated from West Asia, South-East Asia, Central and South America. In view of this, our findings cannot be extrapolated to all low- and middle-income countries. Whereas we have used the number of respondents indicating particular barriers to rank the significance of these barriers in each study, we also recognize that there needs to be a standardized methodology to rank the significance of variables in each study. The interpretation of the review results could only be context specific. Nevertheless, the review still indicates a set of potential barriers that could be considered when discussing breast and cervical cancer screening in any low- and-middle-income country.

### Future research

Further research is needed to assess women’s preferences for cancer prevention screening programmes and their value to the public. Also, more needs to be understood on how husbands and elderly family members can be influenced to support their women to undergo screening. As shown in our review, the opportunity cost of screening is as much a barrier as the cost of screening itself. New studies are needed to provide evidence on how the government should provide incentives to women to undergo screening and what kind of incentives should be provided. Should the screening be free? Should the incentives cover the cost of transportation to the screening centre? These investigations could provide insight into the women’s ability and willingness to pay for screening. To achieve the international goals of women’s cancer prevention, especially breast and cervical cancer prevention, more exploration is needed on whether governments and health systems should adopt population-based screening or targeted screening.

## Conclusion

Our systematic review found that while a lack of awareness, embarrassment, lack of family support and cost of screening were important barriers, the fear of diagnosis of cancer due to lack of finances for subsequent tests and treatment was the most significant barrier and had an overarching influence on the other barriers. Free cancer screening and nudges or incentives for cancer screening may increase uptake. However, much more needs to be done by governments in the area of financing cancer treatment, either fully or partially. Self-testing for cervical screening could be a panacea for several key barriers, such as embarrassment or shyness to undergo screening, affordability and lack of spousal support. Clinical breast examinations could be considered for population screening of breast cancer in low- and middle-income countries.

## Supplementary Material

czac104_Supp

## Data Availability

All relevant data are in the paper and its supporting information files.

## Reflexivity statement

The authors include one female and three males who specialize in diverse subjects within health care. F.v.M. and M.P. are Professors of Health Economics and Healthcare Operations respectively. Both have done extensive research in public health in Europe, Asia and Africa. S.V.R. is a medical engineering scientist who is also an entrepreneur and runs two companies in the areas of renal and ophthalmic diseases. A.S. is a health-care administrator with a background in health policy.
