# Supplementary material for: Barriers to cervical cancer and breast cancer screening uptake in low- and middle-income countries: a systematic review
Source: Health Policy Plan. 2022 Dec 16;38(4):509–27. doi: 10.1093/heapol/czac104 (PMC10089064; doi:10.1093/heapol/czac104)
Supplement: czac104_Supp [file czac104_supp.zip › Appendix_1_-_Search_Strategy.docx]

***Appendix 1 - Search Strategy***

The final keywords chain used in the systematic literature search in PubMed were as follows:

**#1** *MESH DESCRIPTOR Barriers to breast and cervical cancer screening*

**#2** *((Barriers or challenges))*

**#3** *#1 OR #2*

**#4** *MESH DESCRIPTOR Breast*

**#5** *MESH DESCRIPTOR Cervical*

**#6** *MESH DESCRIPTOR Cancer Screening*

**#7** *#4 AND #5 AND #6*

**#8** *((Breast OR Breast Cancer OR Breast Neoplasms))*

**#9** *((Cervix OR Cervical OR Cervical Neoplasms))*

**#10** *((Screening OR Population Screening OR Mass Screening))*

**#11** *((Early detection of Breast Cancer OR Breast self-examination OR Mammogram OR Mammography))*

**#12** *((Early detection of Cervical Cancer OR Pap Smear OR Papanicolaou test OR VIA OR Visual inspection with acetic acid))*

**#13** *((HPV OR Human Papillomavirus OR HPV Vaccination))*

**#14** *((Female Cancers))*

**#15** *#8 OR #9 OR #10 OR #11 OR #12 OR #13 OR #14*

**#16** *MESH DESCRIPTOR Low and Middle-Income countries*

**#17** *((Low-income economies OR Lower-middle income economies OR Upper-middle income economies OR low-resource OR limited-resource OR poor-resource OR scarce-resource OR resource-constraint OR under-developed OR less-developed OR least-developed OR developing countries OR third-world countries))*

**#18** *((Afghanistan OR Burkina Faso OR Burundi OR Central African Republic OR Chad OR Congo Dem. Rep OR Eritrea OR Ethiopia OR Gambia* (Bank, 2020) *OR Guinea OR Guinea-Bissau OR Haiti OR Korea Dem. People's Rep. OR Liberia OR Madagascar OR Malawi OR Mali OR Mozambique OR Niger OR Rwanda OR Sierra Leone OR Somalia OR South Sudan OR Sudan OR Syrian Arab Republic OR Togo OR Uganda OR Yemen, Rep. OR Angola OR Algeria OR Bangladesh OR Belize OR Benin OR Bhutan OR Bolivia OR Cabo Verde OR Cambodia OR Cameroon OR Comoros OR Congo, Rep. OR Côte d'Ivoire OR Djibouti OR Egypt Arab Rep. OR El Salvador OR Eswatini OR Ghana OR Haiti OR Honduras OR India OR Indonesia OR Iran, Islamic Rep OR Kenya OR Kiribati OR Kyrgyz Republic OR Lao PDR OR Lesotho OR Mauritania OR Micronesia, Fed. Sts. OR Mongolia OR Morocco OR Myanmar OR Nepal OR Nicaragua OR Nigeria OR Pakistan OR Papua New Guinea OR Philippines OR Samoa OR São Tomé and Principe OR Senegal OR Solomon Islands OR Sri Lanka OR Tanzania OR Tajikistan OR Timor-Leste OR Tunisia OR Ukraine OR Uzbekistan OR Vanuatu OR Vietnam OR West Bank and Gaza OR Zambia OR Zimbabwe OR Albania OR American Samoa OR Argentina OR Armenia OR Azerbaijan OR Belarus OR Bosnia and Herzegovina OR Botswana OR Brazil OR Bulgaria OR China OR Colombia OR Costa Rica OR Cuba OR Dominica OR Dominican Republic OR Equatorial Guinea OR Ecuador OR Fiji OR Gabon OR Georgia OR Grenada OR Guatemala OR Guyana OR Iraq OR Jamaica OR Jordan OR Kazakhstan OR Kosovo OR Lebanon OR Libya OR Malaysia OR Maldives OR Marshall Islands OR Mauritius OR Mexico OR Moldova OR Montenegro OR Namibia OR North Macedonia OR Panama OR Paraguay OR Peru OR Romania OR Russian Federation OR Serbia OR South Africa OR St. Lucia OR St. Vincent and the Grenadines OR Suriname OR Thailand OR Tonga OR Turkey OR Turkmenistan OR Tuvalu))*

**#19** *#16 OR #17#18*

**#20** *((approachability* OR acceptability* OR availability* OR affordability* OR appropriateness* OR awareness* OR fear* OR out-of-pocket OR autonomy OR utilization))*

**#21** *#3 AND #7 AND #15 AND #19 AND #20*

**#22** *#21 AND English [Language] AND (“2016” [Date - Publication]: “2020” [Date - Publication]).*

The chain of keywords used in the other two searches (Mendeley and Google Scholar) was similar, depending on the technical limitations in the search in those databases.
