# Supplementary material for: Barriers to cervical cancer and breast cancer screening uptake in low- and middle-income countries: a systematic review
Source: Health Policy Plan. 2022 Dec 16;38(4):509–27. doi: 10.1093/heapol/czac104 (PMC10089064; doi:10.1093/heapol/czac104)
Supplement: czac104_Supp [file czac104_supp.zip › Appendix_3_-_Extraction_Matrix.docx]

| **Nr** | **Reference; year of publication; title; type of publication and country** | **Objective of study; Study setting; Study Design; Study Population** | | **Evaluation of study; Type of analysis; Validity and Reliability** | **Main findings: access** | | | | | | | | **Conclusion and policy recommendations** |
| --- | --- | --- | --- | --- | --- | --- | --- | --- | --- | --- | --- | --- | --- |
|  |  |  |  |  | Approachability | Acceptability | Availability | Affordability | Appropriateness | Awareness | Angst | |  |
| 1 | Austad et al, 2018, Barriers to Cervical Cancer Screening and the Cervical Cancer Care Continuum in Rural Guatemala: A Mixed-Method Analysis, Journal Article, Guatemala | **Objective**: To understand factors that affect use of screening services and loss to follow-up along the care continuum  **Study Setting**: PHCs named Maya Health Alliance in 14 villages in Central Guatemalan provinces of Chimaltenango and Suchitepéquez - screening programs  **Study design**: Mixed methods study, Retrospective EMR reviews of patients & structured interviews  **Study population**: 515 women patients aged between 16 & 75 years and 54 women | | **Type of analysis**: Quantitative Analysis – Descriptive Analysis, Qualitative Analysis – Exploratory Analysis  **Validity (Internal)**: The data set was checked for accuracy by comparing abstracted information with that of the EMR. The Qualitative data was synthesized by 2 authors and reviewed by the 3rd author. Underreporting and misclassification likely due to self-reporting of prior cervical screening  **Validity (External)**: Not discussed but medium representativeness - Data collected from 2 provinces. Findings not generalizable due to multiple overlapping services  **Reliability (repetitiveness)**: Mixed methods. | **Approachability:** Two (3.7%) of the 54 women expressed distrust in results  **Acceptability:** Eight (14.8%) of the 54 mentioned community-level gossip about ill effects of screening, but  only one (1.9%) of the 54 interviewees had been specifically discouraged by a family member (her mother).  Twenty-one (39%) of the 54 women reported restricted agency (asking permission from their spouses to be screened).  **Availability:** Travel to reach screening was prohibitive. (9%; five of 54 women),  Regarding free-of-charge screening, 33.3% (18 of 54 women) reported that free public clinics only intermittently offered screening and that attending specific days was difficult.  **Affordability:** Out-of-pocket costs were a common reason (20%; 11 of 54 women) for not seeking screening or for not repeating screening with the recommended frequency,  cited difficulties with missing work. (59%; 32 of 54 women), difficulties with arranging childcare.  **Appropriateness:** 9%; five of 54 women reported never receiving their screening results or  not being offered follow-up for abnormal findings.  **Awareness:** (6.1%; three of 49 women) linked cervical cancer to use of family planning methods or to multiparity (4.1%; two of 49 women).  16.3% (eight of 49 women) felt that cervical cancer was caused by a vaginal infection and described symptoms (discharge, bleeding, or pain), but none mentioned asymptomatic HPV infection. | | | | | | | **Conclusion:**  Care cascade analysis paradigm to foster cross-site comparisons focused on improvement in the quality of cancer care in low-resource settings  **Policy Recommendations**  Guatemala needs to adopt and implement evidence-based education strategies, such as the public marketing campaigns shown to improve screening in Honduras | |
| **2** | Kung et al, 2019, “My husband says this: If you are alive, you can be someone…”: Facilitators and barriers to cervical cancer screening among women living with HIV in India, Journal article, India | **Objective:** To evaluate individual and interpersonal factors influencing cervical cancer screening among women living with HIV  **Study Setting:** New Civil Hospital (NCH) ART Centre in Surat, India, an outpatient clinic that provides HIV care to approximately 50–60 women per day  **Study design:** In-depth semi-structured interviews  **Study population:** 25 WLWH (women living with HIV) and 15 stakeholders (healthcare workers) | | **Type of analysis:** Qualitative Analysis - Directed content analysis  **Validity (Internal):** Small number of stakeholders and patients from one public hospital in Surat. Triangulation of data.  **Validity (External):** Is not representative of those who are lost to follow-up and not receiving HIV care, women who have HIV but have not yet been diagnosed, or women without HIV. Will not be representative of patients in other regions of India  **Reliability (repetitiveness):** Not discussed. Due to triangulation of data, reliability high. | **Approachability:**  (n = 14) patients reported that detailed information was not provided to patients regarding cervical cancer screening.  (n = 7) workers expressed inability to provide full instruction to patients due to high patient volume.  **Acceptability:** (n = 15) patients indicated family support was a key facilitator to get Pap test.  (n = 7) workers stated that there are patients that do not have family members who provide support.  (n = 3) workers indicated that a patient’s partner may actively discourage her from getting the Pap test.  (n = 3) noted that some patients did not have instrumental support from family with work or childcare.  The majority of patients (n = 17) interviewed were motivated to learn more information about cervical cancer and cervical cancer screening  **Appropriateness:** Lack of communication between a patient and her healthcare provider led to her receiving unnecessary medical care; specifically, a Pap test in the absence of a cervix  (n = 9) workers and patients reported that some patients were not treated well by the health system.  **Awareness:** Most patients lacked complete knowledge about Pap tests and cervical cancer (n = 23).  **Angst (Fear):** (n = 19) patients had fear of the Pap test and of the subsequent results. | | | | | | | **Conclusion**  Women in the study lacked knowledge regarding cervical cancer screening are consistent with previous studies conducted in India  **Policy Recommendations:**  1. Enhanced public education required regarding cervical cancer, in OBG clinics one-on-one with women and also using lower literacy communication interventions to educate the families and society.  2. Increase the number of healthcare providers trained to perform cervical cancer screening.  3. Tailored education to communicate the increased risk of cervical cancer among women with HIV | |
| **3** | Koneru et al, 2017, Acceptance of peer navigators to reduce barriers to cervical cancer screening and treatment among women with HIV infection in Tanzania, Journal article, Tanzania | **Objective:** To identify barriers to cervical cancer screening and treatment, and determine acceptance toward peer navigators (PNs) to reduce barriers  **Study Setting**: 12 public-sector HIV CTCs (Care & Treatment Clinics) in Dar es Salaam run by Management & Development for Health  **Study design:** Cross-sectional study using PNM (Patient Navigation Model)  **Study population**: 399 Women with HIV infection aged 19 years or older attending HIV clinics in Dar es Salaam, Tanzania | | **Type of analysis:** Quantitative Analysis - Descriptive analyses and Fisher exact test  **Validity (Internal):** Small sample size for screened women and social desirability bias since data is self-reported  **Validity (External):** Participants recruited from clinics. So, will not be generalizable to HIV women who do not access CTCs  **Reliability (repetitiveness):** Because of the small sample size, and lack of representative sample, inference will not be reliable | **Approachability:** Two-thirds of unscreened women reported that no one at the CTC had told them about cervical cancer.  Two-thirds said that the queue to receive follow-up services for a positive screening result is too long  **Acceptability:** 19% of participants thought that it was embarrassing to be screened for cervical cancer.  Almost all women said that they would prefer to be examined by a female doctor and that they would feel more comfortable with a female nurse in the room if they were being examined by a male doctor  **Availability:** When the distance to the clinic was 31-60 minutes and 61-120 minutes, higher proportion of unscreened vs screened people. Beyond 120 minutes, the reverse was true.  **Awareness:**  52.1 % did not know that cervical screening is free and 57.3% did not know that Cervical cancer treatment is free  Approximately half the women did not know that HIV infection increased the risk of HPV infection,  and one-fifth did not know that a woman can have cervical cancer and not know it.  90% knew that early detection and treatment of cervical lesions can prevent development of cervical cancer but only 9% had been screened - indicating a lack of connection between this general knowledge of cervical cancer and the need or urgency to be screened. 95.7% did not know what Patient Navigators (PNs) do.  **Angst (Fear):** A large proportion of both unscreened and screened women thought that the cervical examination was painful | | | | | | | **Conclusion:** 97.5% of the participants on knowing about the role of PNs said they would like the help of PNs to give them awareness and accompany them for cervical screening.  **Policy Recommendation**  PNs are more likely to be accepted because PNs are often from the same communities as the individuals in need of health services, and share similar socioeconomic characteristics and health conditions  The use of PNs would represent a novel approach and a systematic mechanism for addressing barriers to screening | |
| **4** | Feyisa et al, 2018, Perceived Benefits and Barriers toward Cervical Cancer Screening among Women ≥15 Years in Arsi Zone, South-eastern Ethiopia: Application of the Health Belief Model in a Community‑Based Cross‑Sectional Study, Journal article, Ethiopia | **Objective:** To describe women’s perceived benefits and barriers to cervical cancer screening and their associated factors  **Study Setting:** Arsi zone, South-eastern Ethiopia  **Study design:** community‑based cross‑sectional study, multi‑stage sampling technique based on HBM (Health belief Model)  **Study population:** 906 women aged >15 year | | **Type of analysis:** Quantitative Analysis -binary logistic regression model  **Validity (Internal):** Social desirability bias due to self-reports and interviewer-based questionnaires  **Validity (External):** The number of participants was proportionally distributed into the four districts to increase the representativeness of the sample and to reduce sampling bias. Large sample size, representativeness not compromised.  **Reliability (repetitiveness):** Proportional allocation by multi-stage sampling technique increases reliability | **Approachability:** 501 (55.3%) felt lack of recommendations from health professionals is a barrier.  **Acceptability:**  315 (34.8%) felt embarrassed to undergo a CC screening.  312 (34.5%) felt lack of recommendation from husband is a barrier.  **Availability:** 566 (62.7%) felt lack of availability of services were a barrier.  **Affordability:** 287 (31.7%) felt that cost of cervical screening is a barrier  **Awareness:** 549 (60.6%) participants felt that a lack of information on where to get the services is a barrier.  **Angst (Fear):** 285 (31.4%) feared screening. | | | | | | | **Conclusion:**  A small proportion of the population did not want to undergo screening due to fear, embarrassment, the perception of not being at risk, and the absence of a recommendation from health professionals. The majority of the study population had high perceived benefits and perceived barriers toward cervical cancer screening  **Policy Recommendations:**  Maternal and child health officers should be stationed at each district, to improve awareness of cervical cancer and the benefits of screening and reduce barriers to screening among women | |
| **5** | Hasahya et al, 2016, Beliefs, perceptions and health-seeking behaviors in relation to cervical cancer: a qualitative study among women in Uganda following completion of an HPV vaccination campaign, Journal article, Uganda | **Objective:** To explore beliefs, attitudes, perceptions, and health-seeking behaviours in relation to cervical cancer among women in Uganda after an HPV vaccination project was rolled out.  **Study Setting:** Two rural districts, Nakasongola and Ibanda, found in central and western Uganda  **Study design:** Qualitative study focus group discussions (FGDs)  **Study population:** 36 women, aged 25-49 years, belonging to either the Ankole or Buganda ethnic groups and with no previous history of cervical cancer symptoms or diagnosis | | **Type of analysis:** Qualitative Analysis - Content analysis  **Validity (Internal):** Triangulation and peer review of the collected data were performed and cross-checked with both the participants and the local researcher after every FGD session. For triangulation, trustworthiness validity, and ensuring dependability, a stepwise replication procedure was used  **Validity (External):** Women aged below 25 years and those aged above 50 years were excluded from the study. Women with HIV/AIDS were also excluded from the study because they have their own unique barriers related to disclosure of their HIV status. The findings may not be transferrable to other settings due to the cultural and ethnic diversity in Uganda  **Reliability (repetitiveness):** Not discussed. Triangulation of data, highly reliable. | **Availability:** Accessibility of screening centres,  **Affordability:** Costly journeys  few diagnostic and treatment facilities in the community – needed long and costly journeys to screening centres,  **Appropriateness:** Belief that health workers quite often did not share their findings with clients after diagnosis  but instead preferred to keep the findings a secret,  Services inadequate for all the women who needed to be screened,  Centres understaffed  **Awareness:** Lack of knowledge about cervical cancer explained their poor health-seeking behavior in relation to cervical cancer screening,  belief and perception that screening could diagnose both cervical cancer and HIV/AIDS,  Wrong belief that HPV vaccinations were to prevent their daughters having more than 2 children in future  **Angst (Fear):**  Fear of becoming infected in the health service settings,  fear of contracting cervical cancer infection from their husbands,  fear of reaction of husbands if diagnosed with HIV during cervical cancer screening,  fear that cervical screening is painful and done with unsterilized equipment,  fear of male health practitioners | | | | | | | **Conclusion:**  If women considered barriers to outweigh the benefits of screening, then they had difficulties in complying with screening attendance  Knowledge about cervical cancer and its interrelation with higher cervical cancer screening attendance. Women from rural areas had a limited understanding of female reproductive organs and associated diseases. Even after information was disseminated, the women in Ibanda and Nakasongola districts still maintained misguided beliefs, attitudes, and poor health-seeking behaviours in relation to cervical cancer.  **Policy Recommendations:**  Health promotion efforts should focus on improving women’s knowledge of risk factors.  In order to ensure strong and effective cervical cancer preventive programmes, strategies that focus on cost-effective services and availability and use of the services by the women who need them most are vital.  Health education materials should be appropriate for and ideally matched to the educational levels of particular targeted audiences and need to be tailored towards improving knowledge levels about the risk factors of cervical cancer and the importance of screening, as well as the impact on mortality due to cervical cancer | |
| **6** | Ampofo et al, 2020, A cross-sectional study of barriers to cervical cancer screening uptake in Ghana: An application of the health belief model, Journal article, Ghana | **Objective:** To determine the barriers influencing CC screening among women  **Study Setting:** Ashanti Region of Ghana  **Study design**: Analytical cross-sectional study design  **Study population**: 200 women | | **Type of analysis:** Quantitative Analysis - Health Belief Model using Descriptive statistics and Multi-variate logistic regression  **Validity (Internal):** The likelihood of selecting  more women who were interested in screening may be biased the findings. Pretest was conducted. The researchers explained the questionnaire to the illiterate in the Twi dialect.  **Validity (External):** The major limitation is the less rigorous and biased sampling technique, convenience  sampling, which makes generalizations impossible  **Reliability (repetitiveness):** The Cronbach alpha of most of the questions was 0.934. | Approachability: 172 (86%) alluded that long waiting time is a barrier  Acceptability: While about two thirds 149 (74.5%) of the respondents mentioned they were not susceptible to CC and thus will not screen.  157 (78.5%) mentioned they did not like male health personnel offering screening services.  Affordability: 139 (69.5%) indicated screening was expensive, and 46(23%) could only afford between USD 2–10.  Appropriateness: 133 (66.5%) said lack of communication prevents them from seeking CC screening services  Awareness: Only 36 (18%) of the respondents had adequate knowledge of cervical cancer.  Almost all 190 (95%) of the women surveyed responded that CC was not a sexually transmitted disease  and a little more than half 112 (56%) of the women surveyed indicated that CC could not be prevented.  Also, majority 189 (94.5%) of the respondents mentioned it could not be prevented through vaccination of young girls  110 (55%) did not know of any health facility offering CC screening  Angst (Fear): 160 (80%) thought the screening was scary | | | | | | | **Conclusions:**  1. Married women, unemployed and those with no formal education are less likely to participate in CC screening.  2. Perceived lack of susceptibility, feeling of embarrassment, fear of wrong diagnosis and pains, feeling afraid, high cost, busy work schedule, lack of knowledge on screening facilities, the gender of screener and communication barriers affects women’s interest in participating in screening  **Policy Recommendations:**  1. Ghana health services should develop appropriate, culturally tailored educational materials on CC screening to inform women particularly those with no formal education through social media, television, radio and community information systems to enhance uptake  2. In the short term, the government of Ghana should make policies to incorporate cervical cancer screening to maternal health services such as family planning.  3. In the long run, a national screening program should be instituted to enhance cervical screening uptake.  4. Future studies should use larger sample sizes to make generalizations to improve policy decisions. | |
| 7 | Okunowo et al, 2020, Cervical Cancer Screening among Urban Women in Lagos, Nigeria: Focus on Barriers and Motivators for Screening, Journal article, Nigeria | **Objective:** To assess the factors that serve as barriers and motivators for cervical cancer screening among urban women in Lagos, Nigeria  **Study Setting:** Antenatal and gynecological clinics of Randle General Hospital, Lagos, Nigeria  **Study design:** Descriptive cross-sectional study using structured questionnaires  **Study population:** Women, n = 500 | | **Type of analysis:** Quantitative Analysis  **Validity (Internal):** Not discussed  **Validity (External):** Not discussed, Limited generalizability as data was collected from a small cross-section of participants visiting a general hospital clinic  **Reliability (repetitiveness):** Not discussed | **Approachability:** Non-recommendation by doctors/nurses (41.4%)  **Acceptability:** A belief that they cannot develop cervical cancer (20.1%) was a major barrier  advice from friends and relatives (21.7%)  discouragement by a spouse  test procedure is uncomfortable  and embarrassing.  indecision about doing screening test  **Affordability:** Inability to afford the cost of screening was the obstacle in 12.3% of women  **Awareness:** Identifiable barriers alluded to awareness were:  Lack of awareness of screening methods (64.2%),  Lack of adequate information on screening methods (43.4%),  not knowing when to do the test (27.7%),  58.6%) lack knowledge of methods of cervical screening  **Angst (Fear):** fear of being diagnosed with cervical cancer  Fear of the procedure | | | | | | | **Conclusions**  The uptake of cervical cancer screening is low among urban women in Lagos, Nigeria despite the availability and provision of the screening service as a result of the interplay of several factors that act as either barriers or motivators to its uptake  **Policy Recommendations:**  Health practitioners especially doctors and nurses regularly educate their female clients on the need for regular cervical cancer screening irrespective of the reason for their consultation or presentation at the health facility  The cost of cervical cancer screening be subsidized by the government, so as to encourage as many women as possible to undergo cervical cancer screening  Education of women on the risk factors for cervical cancer will improve the understanding of their susceptibility to cervical cancer  Raise the level of awareness of cervical cancer screening among women using the mass media | |
| 8 | Amos et al, 2019, Perceived Barriers to Uptake of Cervical Cancer Screening Among Women of Childbearing Age in a Gynecological Clinic, Journal article, Nigeria | **Objective:** To assess the perceived barriers to uptake of cervical cancer screening services among women of childbearing age  **Study Setting:** Gynecological outpatient clinic of the University College Hospital, Ibadan, Oyo State. Nigeria.  **Study design:** Descriptive cross-sectional approach and Health Belief Model  **Study population:** Women aged 15-49 years attending the gynecological outpatient clinic Hospital who consented to be part of the study, n=100 | | **Type of analysis:** Quantitative Analysis - Univariate analysis, t-test, Chi-square  **Validity (Internal):** A test-retest method was used  **Validity (External):** The first respondent at each clinic day was selected randomly from the list of eligible respondents  among the patients presenting to the clinic for the day while the remaining respondents were every subsequent  third patient from the list to reduce bias and ensure representativeness.  **Reliability (repetitiveness):** High reliability. | **Approachability:** Lack of information/counselling (77.4%),  **Acceptability:** Cervical cancer screening is embarrassing (35.5%),  Lack of support from partners and significant others (56.5%)  Lack of female health screeners in health facilities (46.8%)  **Availability:** Cervical cancer screening is not available in all health facilities (35.5%),  Lack of convenient clinic time (62.9%)  **Affordability:** Cervical cancer screening is not affordable (35.5%)  **Appropriateness:** Attitude of health workers discourage women from assessing cervical cancer screening (51.6%)  **Awareness:** Belief that only women with STIs should go for cervical cancer screening (22.6%)  **Angst (Fear):** Fear of having a positive result (61.3%),  Cervical cancer screening is painful (33.9%), | | | | | | | **Conclusions:**  The high level of awareness of cervical cancer did not translate to proper utilization of the screening.  Not only barrier but also the other components of HBM theoretical construct of perceptions of seriousness of cervical cancer, susceptibility to the disease and benefits of screening need to be tested in a larger study along with its modifying variables such as culture, past experiences, motivations, etc.  **Policy Recommendations:**  Information on cervical screening should be given regularly to women at every available opportunity supported with the friendly environment for service uptake in term of infrastructure, attitude and removal of economic bottlenecks  Need for nationally appropriate screening programme at all levels of health care for universal coverage  Need for development of policy and framework on health screening  Reminder/Recall services, which can be mobile telecommunication based, should be in place to ensure compliance with appointments to increase utilization | |
| 9 | Jayaraman et al, 2016, Self-perceived risk and barriers to cervical cancer screening among patients seeking care at a tertiary care teaching hospital in Tamil Nadu, Journal article, India | **Objective:** To ascertain the perceptions of risk and barriers to cervical cancer screening by women seeking care at a tertiary care teaching hospital in Tamil Nadu.  **Study Setting:** Department of Obstetrics and Gynecology of a tertiary care teaching institution of Tamil Nadu  **Study design:** Pre-designed structured questionnaire  **Study population:** Women seeking family planning services, n = 177 | | **Type of analysis:** Quantitative Analysis – Chi Square test, Two-tailed P test  **Validity (Internal):** Self-reported data is susceptible to social desirability bias and recall bias.  **Validity (External):** The results obtained may not be generalized findings are only referable to women who accepted to be interviewed and eventually to undergo screening using the visual inspection approaches.  **Reliability (repetitiveness):** Study population consisted of women seeking family planning services and therefore not the true representative of women in the reproductive age group. | **Acceptability:** 32% of the study subjects felt that they were at no risk of developing cervical cancer  and 34.5% felt no need for screening for the condition.  **Affordability:** Lack of finances to buy the services (%age not mentioned)  **Awareness:** Lack of awareness about the screening services (%age not mentioned)  **Angst (Fear):** Fear of positive screening (or abnormal Pap smear) results (%age not mentioned) | | | | | | | **Conclusion:** Self-perception of not being at risk is documented to be associated with low uptake of screening.  **Policy Recommendations:** Health education programs need to bring clearly to the end user the difference between precancerous lesions and invasive cervical cancer and the treatment options available for each of these clinical entities. This would reduce the frequency of the specious fear of abnormal cervical screening findings which was mentioned as a significant barrier to cervical cancer screening | |
| 10 | Onyenwenyi et al, 2018, Barriers to cervical cancer screening uptake among rural women in South West Nigeria: A qualitative study, Journal article, Nigeria | **Objective:** To inform the development of a cervical cancer screening model for use by rural women  **Study Setting**: 14 catchment communities in Sango Primary health center (PHC) of Ado-Odo Ota, Ogun State, Nigeria  **Study design**: Qualitative exploratory research design focus group discussions and in-depth interviews  **Study population:** 28 individuals (13 rural men and 15 rural women) | | **Type of analysis:** Qualitative Analysis - Thematic mapping  **Validity (Internal):** Purposive sampling was used to identify subjects to get a deeper understanding of the issue of enquiry  **Validity (External):** The findings of this study may not be generalizable to other regions due to diverse cultures and religions  **Reliability (repetitiveness):** Observational field notes and responses from rural men and women (different sources) were triangulated to provide multiple perspectives of the information, as well as validate the data. Reliability is high. | **Approachability:** Not explained properly by the community health workers  Doctors not on time  Long hospital waiting time results in Time loss for market and business  **Acceptability:** no consent from husband as husbands do not want wives to be seen by other males,  do not believe in modern medicine  **Availability:** Location of the screening site in urban areas which is too far  **Affordability:** Cost of screening expensive  **Awareness:** No awareness of the screening,  Illiteracy of husbands  **Angst (Fear):** Fear of the test results, | | | | | | | **Conclusion**  Hospital-related barriers, poverty, ignorance and psycho-social barriers (cultural and religious) negatively influenced screening uptake.  **Policy Recommendations:**  (i) male involvement, including spouses, and religious and cultural leaders;  (ii) cervical cancer screening services should be integrated into primary healthcare centres as a strategy to improve screening access; and  (iii) the government should consider providing free or subsided cervical cancer screening for rural women. | |
| 11 | Lori Brand Bateman et al, 2019, Barriers and Facilitators to Cervical Cancer Screening, Diagnosis, Follow-Up Care and Treatment: Perspectives of Human Immunodeficiency Virus-Positive Women and Health Care Practitioners in Tanzania, Journal article, Tanzania | **Objective**: To identify barriers and facilitators to cervical cancer screening, diagnosis, follow-up care, and treatment among human immunodeficiency virus (HIV) infected women and clinicians and to explore the acceptability of patient navigators in Tanzania  **Study Setting:** Management and Development for Health (MDH) public HIV CTCs (Care & Treatment Clinics) in Dar es Salaam, Tanzania.  **Study design:** Four focus groups, two with HIV-positive women and two with clinicians  **Study population:** Four focus groups, two (n=10 and n=9) with HIV-positive women, 19 years or older, who had been screened for cervical cancer and two with male (n=7) and female (n=14) clinicians (physicians and nurses; n=12 and n=9) | | **Type of analysis:** Qualitative Analysis – Thematic Analysis  **Validity (Internal):**  Only HIV-positive women were chosen for the study under the presumption that HIV-positive women are at greater risk for cervical cancer than HIV-negative women. The small sample size and narrow nature of the sample are limitations  of this study  **Validity (External):** The small sample of women who participated in the focus group discussion sessions precludes  generalization of the results to larger population groups of  women in Tanzania  **Reliability (repetitiveness):**  Only HIV-positive women who had been screened for cervical cancer were included in the focus group discussion sessions, so the findings could be different for  women who had not been screened. | **Approachability:** Lack of information given by the healthcare providers to women about cervical cancer  **Acceptability** Despite availability, women avoided screening either to  lack of knowledge  of its importance  **Affordability:** Despite screening being free of cost,  **Awareness:**  Erroneous beliefs that cervical cancer is a disease that attacks the womb either through the blood or sexual transmission, or through genes.  Viewed by women as a ‘death sentence’  A wrong understanding that screening should be performed after symptoms occurred  **Angst (Fear):** a fatalistic belief that one dies early from cancer made women avoid screening  Fear and stigma of isolation by husband and society. | | | | | | | **Conclusion:**  Patient navigation could help in assisting women with cervical cancer education, screening, diagnosis, follow-up care, and treatment. This could increase the number of women accessing these services and ultimately lead to a reduction in cervical cancer diagnoses among the women  **Policy Recommendations**  Ministry of Health needs to address provider concerns such as training (need more training on screening and treatment of precancerous lesions, not just theory but practice also) and adequate space and infrastructure. | |
| 12 | Rasul et al, 2016, Barriers to cervical cancer screening among Iraqi Kurdish women: A qualitative study, Journal article, Iraq | **Objective:** To explore perceived barriers to cervical cancer screening from the viewpoint of Kurdish women from Kurdistan Region, Iraq  **Study Setting:** Private room in the pap smear center  **Study design:** Semi-Structured in-depth interviews  **Study population:** 19 women from Kurdistan region in Iraq | | **Type of analysis:** Qualitative Analysis - Content analysis approach according to Krippendorff’s method  **Validity (Internal):**  The participants were selected  based on maximum variation sampling  **Validity (External):**  For achieving conformability, the results were delivered to several non-participating women who were asked to compare the results with their experiments. This confirmed the rich description of the data and ensured the fitness and transferability of the research  **Reliability (repetitiveness):** Four operational techniques, namely credibility, transferability, dependability, and confirmability, were used to ensure trustworthiness in the present study | **Approachability:** Health workers did not explain why it was needed, what benefits it had, and when it had to be performed,  **Acceptability:** embarrassment, No symptoms  **Availability:** Only 4 centres, 1 each in the capital of each Kurd state.  **Appropriateness:** Healthcare providers had no time to explain what a Pap test was,  mistrust in the hospital facilities – especially of dirty and unsterile equipment  and the smear taker.  **Awareness:**  Inappropriate awareness & lack of awareness – pap test needed only when women are old, married or symptomatic,  Incorrect belief related to cervical cancer screening purpose and importance – purpose of pap test is to detect the existence of cervical cancer,  superstitions – if you trust in God, one won’t get cervical cancer.  **Angst (Fear)**:  Fear of pain during the test,  fear of death  from a positive or abnormal result,  discomfort from the speculum during the gynecological exam, | | | | | | | **Conclusion**  The findings of this study suggested a combination of factors to prevent women from having a Pap test - low awareness, psychological factors such as fear, discomfort and embarrassment, and unavailability of screening services  **Policy Recommendations**  Health promotion policies are necessary to increase Kurdish women's knowledge and awareness and correct their beliefs  Effective information provision and communication between women and healthcare providers are necessary.  The Ministry of Health should establish adequate centres and oblige healthcare providers to engage in women’s education about a Pap test | |
| **13** | Nancy Innocentia Ebu, 2018, Facilitators and barriers to cervical cancer screening among HIV-positive women in Ghana, Journal article, Ghana | **Objective:** To describe the barriers to and facilitators of cervical cancer screening among HIV-positive women attending a Ghanaian primary health care facility that offered antiretroviral therapy for HIV  **Study Setting:** An antiretroviral therapy (ART) clinic in a primary health care facility in Elmina, part of the Central Region of Ghana.  **Study design:** Qualitative multiple case study  **Study population:** 325 HIV-positive women | | **Type of analysis:** Qualitative analysis - directed approach to content analysis  **Validity (Internal):** The use of health belief model and the theory of planned behaviour poses some  limitations to the naturalistic paradigm, such as a higher  possibility of the research findings supporting the model than disproving it There is also the.  possibility of interviewer bias, because questions were probing and may have influenced participants to answer the questions in a certain way.  **Validity (External):** This study does not aim to transfer its findings to  other settings  **Reliability (repetitiveness):** Dependability of the findings was maintained by ensuring that all field notes, tape recordings, data analysis, themes,  interpretations, personal reflections and descriptions of how  the data were obtained and analysed were compiled. | **Acceptability:** did not believe they were susceptible to cervical cancer,  believed that they would never get it  **Availability:** Test not available in the primary health care facility  **Affordability:** Financial challenges, as the test is not free, complaints of lack of finances to pay for the test even if it was available  **Awareness:**  responses indicated a low level of knowledge of cervical cancer,  had no idea about the mode of transmission,  signs and symptoms  and the fact that cervical cancer screening could identify symptoms of cervical cancer. | | | | | | | **Conclusion**  Although HIV-positive women may have the intention to seek cervical cancer screening, however, low knowledge of cervical cancer, HIV-positive women perceiving themselves as not being susceptible, the unavailability of cervical cancer screening test in primary health care facilities, and financial implications can potentially deter high-risk women.  **Policy Recommendations**  comprehensive education on cervical cancer and screening integrating cervical cancer screening into HIV programmes | |
| **14** | Lunsford et al, 2017, Environmental and Psychosocial Barriers to and Benefits of Cervical Cancer Screening in Kenya, Journal article, Kenya | **Objective:** To explore perceptions about barriers to and benefits of cervical cancer screening.  **Study Setting:** Urban Nairobi and rural Nyanza, South-western Kenya  **Study design:** Focus groups  **Study population:** 60 women aged 25-49 years and 40 male partners | | **Type of analysis:** Qualitative Analysis - Thematic  **Validity (Internal):** As research was conducted by KEMRI/CDC, a respected institution in Kenya, responses may have been influenced by  social desirability. Pilot conducted. Reviewers trained in qualitative analysis.  **Validity (External):** Participants were all from Nairobi and Nyanza;  therefore, findings may not be generalizable to other geographic  regions of Kenya.  **Reliability (repetitiveness**): Not discussed. Triangulation of data, so higher reliability, | **Acceptability:**  Spousal approval, embarrassment during screening,  men are not always accepting of women going for a screening,  men reported that a woman who goes for screening might be suspected of being promiscuous,  concerns with having a male doctor perform screening tests, young age of a doctor as a barrier,  **Availability:**  Poor screening availability,  transportation difficulties,  long travel times to get to a screening site,  **Affordability:**  Unscreened women and male partners reported that screening was “too expensive,”  and some would not get screened without incentives,  lack of cost-effective transportation  lack of time (screening takes too much time)  **Awareness:** Lack of knowledge, religious or cultural beliefs.  Neither women nor men mentioned screening as a beneficial method of detecting precancerous lesions before they develop into cancer.  Women lack information about (a) cancer and cervical cancer awareness, (b) who gets cancer, (c) signs and symptoms of cancer, and (d) the benefits of screening and what occurs during different screening procedures  Misconceptions about speculum use causing infertility  **Angst (Fear):** stigma, fear of residual effects of test results, stigma as a barrier, specifically being “talked about,” “judged,” discriminated against, fear of getting positive results | | | | | | | **Conclusion**  The study examines potential differences in perceptions between those dwelling in urban versus rural areas in Kenya  **Policy Recommendations**  There is a need for increased informational, educational, and communication (IEC) outreach to residents throughout Kenya before implementing additional screening efforts. | |
| **15** | Al-Amro et al, 2020, Factors Associated with Cervical Cancer Screening Uptake: Implications for the Health of Women in Jordan, Journal article, Jordan | **Objective:** To determine the factors associated with cervical cancer screening uptake among Jordanian women  **Study Setting:** 8 nongovernmental organizations and community settings in Amman  **Study design:** cross-sectional/correlation study  **Study population:** 500 women aged between 21-65 years | | **Type of analysis:** Quantitative Analysis - Descriptive statistics, multivariate logistic regressions, and independent t-tests  **Validity (Internal):** Information bias connected with self-reporting might have influenced the findings because some women might have felt uneasy about reporting a negative response. English-Arabic-English back translation. Pilot study conducted.  **Validity (External):** Because the participants were residents of Amman, the findings may not be generalisable to other women living in  Jordan.  **Reliability (repetitiveness):** Cronbach’s  alphas for the pilot study and main study were  0.75 and 0.78, respectively, indicating a good level of  reliability. | **Approachability:** Health services encourage women to have Pap smear in (293/500)  **Acceptability:** reasons for refusing to see a male physician were embarrassment, shame over exposing body parts, religious beliefs, husband’s disapproval, and the ability to share feelings. Presence of male physician prevents having Pap smear (215/500), most participants (87.20%) stated that they preferred that a female physician perform the Pap smear  **Availability:** No access to health services where Pap smear is available (214/500), Transportation needed (382/500), inconvenience of clinic time (194/500)  **Affordability:** not willing to pay the cost of Pap smear (328/  **Awareness:** Misconceptions that only women who have had babies need to do cervical cancer screening, Cervical cancer only happens to women who are above the age of 50 years.  34% did not know if screening services were available in the health sector they utilised | | | | | | | **Conclusion**  The rate of cervical cancer screening among Jordanian women of all ages is low. It was found that predictors of cervical cancer screening are healthcare providers’ encouragement, years of marriage, and the use of private sector services. There were significant differences in perceived barriers to cervical cancer screening among women who had had a Pap smear and women who had never had one  **Policy Recommendations**  Community-based screening programmes involving men’s participation where Healthcare providers, physicians, nurses, and midwives should also play their part in increasing women’s awareness and knowledge about the benefits of cervical cancer screening because it has been shown that women need to be encouraged to undergo screening. | |
| **16** | Weng et al, 2020, Women’s knowledge of and attitudes toward cervical cancer and cervical cancer screening in Zanzibar, Tanzania: a cross-sectional study, Journal article, Tanzania | **Objective:** To describe women’s awareness of cervical cancer and to explore the attitudes toward, acceptability of and barriers to cervical cancer screening (CCS) in Zanzibar  **Study Setting:** 10 districts in Zanzibar  **Study design:** cross-sectional study  **Study population:** 1483 women | | **Type of analysis:** Quantitative Analysis - Chi-square tests, analysis of variance (ANOVA) and stepwise multiple Regression  **Validity (Internal):** Most information was self-reported, which might have caused over or underestimation of certain variables. Analyses were cross-sectional and implied only correlation; further research is needed to untangle the causal associations. Data collected via face-to-face interviews.  **Validity (External):** The study included all districts in Zanzibar, as some districts are more rural than others; however, due to the limited samples, the district effect on women’s willingness to participate in free or non-free screening was not  checked.  **Reliability (repetitiveness):** To increase the reliability of the information, the interviewers were trained to administer the questionnaires in a uniform way to prevent their own interpretation of the questions. | **Acceptability:**  Women with a history of schistosomiasis were willing to participate in free or non-free screening.  Women who were married had a higher acceptance of screening than those who were divorced or unmarried.  Other barriers were: women feeling healthy,  women saying screening was unnecessary,  **Affordability**: 34.89% (455/1304) would not pursue screening if they needed to pay for it.  Screening willingness was largely influenced by family income.  Women who had obtained tertiary education were less willing to pay for screening,  **Awareness:** Women’s knowledge of cervical cancer was generally inadequate and was persistently associated with education, family income and family cancer history.  Oral contraceptives, condom usage and swimming in public pools were considered risk factors for cervical cancer  **Angst (Fear):** Fear of screening and inconvenience, perceiving fear of positive results and the fear of pain of the screening | | | | | | | **Conclusion**  Study is the first to indicate that schistosomiasis infection was a significant positive predictor of cervical cancer screening uptake.  **Policy Recommendations**  Women in the younger age and older age groups should receive strengthened education about screening in places where they are likely to gather according to their traditions | |
| **17** | Collins et al, 2019, A descriptive analysis of health practices, barriers to healthcare and the unmet need for cervical cancer screening in the Lower Napo River region of the Peruvian Amazon, Journal article, Peru | **Objective:** To undertake a descriptive analysis of the health needs, healthcare practices and barriers to accessing healthcare faced by women in Lower Napo River Region, Peru, and to understand health literacy regarding cervical cancer and the need for more effective cervical cancer screening services  **Study Setting:** six communities along the Lower Napo River, Loreto, Peru,  **Study design:** Demographic & Health Survey  **Study population**: 121 women | | **Type of analysis:** Quantitative Analysis – Comparison with ENDES 2014 survey  **Validity (Internal):** Recall bias when reporting medical history. Selection bias in the results  **Validity (External):** Data were collected  from a relatively small number of respondents  within the LNR region, with participation limited to 55%– 83% of eligible community members at the time of the survey.  Limited generalisabilty of the results.  **Reliability (repetitiveness):** Not discussed | **Availability:** lack of access to services (53.6%, N = 60/112).  **Affordability:** costly - lacking money for treatment remained the leading barrier to accessing care in this population, time-intensive  **Appropriateness:** ineffective processing of results  **Awareness:** Over two-thirds of women surveyed had ever heard of breast cancer (75.7%, N = 87/115) and 42.2% (N = 49/116) of women knew someone who has or has had breast cancer, but only 32% (N = 37/116) could identify one of the main symptoms.  Less than a third of women had ever heard of cervical cancer (32.7%, N = 34/116) with across the respondents 20% reported knowing someone who had been diagnosed with cervical cancer (N = 23/115).  Only half of respondents felt comfortable explaining what breast cancer is (50%, N = 58/116).  just over a fifth who felt they could explain what cervical cancer is (22%, N = 25/116).  **Angst (Fear):** Fear surrounding the screening process (70.8%, N = 80/113), | | | | | | | **Conclusion**  Research demonstrates prominent within-country disparities in both women’s health and socio-economic indicators in Peru and highlights the importance of the social and structural determinants of health in geographically remote and impoverished river communities of the Amazon.  Gains have not been experienced equitably across the population, marked inequities between the women living in communities across the LNR and national ENDES data.  **Policy Recommendations**  Suggesting a need for programmes appropriately tailored to the needs of communities.  National screening programmes that account for cultural and geographic disparities between indigenous and non-indigenous populations | |
| **18** | Nyblade et al, 2017, A qualitative exploration of cervical and breast cancer stigma in Karnataka, India, Journal article, India | **Objective:** To explore breast and cervical cancer stigma in Karnataka  **Study Setting:** St. John’s’ Medical College and Hospital in Bengaluru for the Breast Cancer Study and 2 rural districts close to Bengaluru where Cancer Care India (CCI) had conducted Cancer Screening Camps for Cervical Cancer  **Study design:** 2 Qualitative Exploratory Studies  **Study population:** 206 participants including patients, primary caregivers and healthcare providers, 59 for Breast cancer and 147 for Cervical cancer | | **Type of analysis:** Thematic analysis & Coding  **Validity (Internal):** Training and supervision aimed to minimize potential interviewer biases. Interviewers had masters’ degrees and 5 years of experience and on-the-job training on survey and qualitative research data collection. To ensure consistency, two members of each respective team double coded a small number of  transcripts, compared their results.  **Validity (External):** Both urban and rural settings - generalisable  **Reliability (repetitiveness):** Not discussed. Triangulation of data – high reliability. | **Approachability:** Gossip and distrust in confidentiality of medical providers  **Awareness:** Negative beliefs – and the resulting blame – that cancer is a punishment for misdeeds either in the current or past life  personal responsibility for having caused cancer,  **Angst (Fear):** fear of cancer transmission;  fear of the inevitability of disability and death with a cancer diagnosis.  belief in and Concern that a diagnosis would lead a husband to stray | | | | | | | **Conclusion**  The causes of cancer stigma described by respondents are similar to what drives stigma around other diseases such as HIV  **Policy Recommendations**  One key recommendation for programmatic action to address more clearly and consistently the misconceptions around transmission and, in particular, the specific fears and imagined pathways. Leverage support of family and community members. | |
| **19** | Olasehinde et al, 2018, Barriers to Mammography Screening in Nigeria: A Survey of Two Communities with Different Access to Screening Facilities, Journal article, Nigeria | **Objective:** To evaluate breast cancer screening practices and barriers in two Nigerian communities with different geographic access to screening facilities  **Study Setting**: Ife Central Local Government, where mammography services are offered & Iwo Local Government, where there are no mammography units  **Study design:** 35 item study specific interviewer administered questionnaire  **Study population**: 1,169 participants in Ife & 1053 participants in Iwo | | **Type of analysis:** Quantitative Analysis – Stratified Analysis, chi-square test. simple and multivariate Poisson regression  **Validity (Internal):**  Pre-testing of 20 market women in Ife was done in both languages English and Yoruba with subsequent survey revision before final adoption.  **Validity (External):** Participants were selected from all the 11 wards in Ife central local district and 14 wards in Iwo Local district. The number of women sampled from each ward was proportional to the fraction of the total population the ward represented  **Reliability (repetitiveness):** Not discussed | **Approachability:** difficulty in obtaining an appointment  **Acceptability:** embarrassment.  **Availability:** challenges locating a mammography facility,  **Affordability:** only 20.4% could afford annual mammography  **Awareness:** Only 138 women (11.4%) from Ife Central and 120 women (11.6%) from Iwo were however aware of mammography.  Despite mammography service availability in Ife Central, 88.2% of the respondents were unaware of its existence  **Angst (Fear):** fear of test results | | | | | | | **Conclusion**  Geographic access without awareness creation and community mobilization does not guarantee utilization of breast cancer screening services.  **Policy Recommendations**  Community education programmes must address the lack of perceived breast cancer risk. | |
| **20** | Getachew et al, 2019, Cervical cancer screening knowledge and barriers among women in Addis Ababa, Ethiopia, Journal article, Ethiopia | **Objective:** To assess cervical cancer screening knowledge and barriers for screening uptake among women in Addis Ababa Ethiopia  **Study Setting:** 13 PHCs of Addis Ababa  **Study design:** A Mixed methods facility-based cross-sectional study  **Study population:** 520 women for Quantitative study, 37 women for the FGDs | | **Type of analysis:**  Quantitative Analysis – Descriptive Statistics, Multivariate logistic regression using adjusted odds ratio  Qualitative Analysis – Thematic Analysis  **Validity (Internal):** Questionnaire translated from English to Amharic back to English. FGDs conducted by experts.  **Validity (External):** A multi-stage sampling technique was used to select women visiting a primary health centre. Proportional allocation of the sample to the respective health centres were done followed by a  systematic sampling procedure to include women in the respective health centres in 13 out of 37 PHCs in 4 sub-cities of Addis Ababa  **Reliability (repetitiveness):** Study design, sampling technique used, High reliability. | **Approachability:** Health providers do not push women to undergo screening,  **Acceptability:** will not undergo screening because of absence of symptoms (57%),  **Availability:** Lack of a screening service in their living area (42.2%), service is not available at their nearest health Centre and it is not even available at all hospitals  **Affordability:** the high cost of screening  **Appropriateness:** not all professionals offer the service and lack of personnel at the screening centres  **Awareness:** Only 42.7% had heard of cervical cancer screening. 56.3% said that they do not know about screening  **Angst (Fear):** fear of the outcome of screening | | | | | | | **Conclusion** Cervical screening knowledge was low among women and less than half had heard of screening. Women also had low experience of screening.  **Policy Recommendations**  Awareness campaigns and education should be undertaken by health professionals. Access and availability of screening service is also essential to improve screening uptake. | |
| **21** | Modibbo et al, 2016, Qualitative study of barriers to cervical cancer screening among Nigerian women, Journal article, Nigeria | **Objective:** To explore the barriers to cervical cancer screening, focusing on religious and cultural factors, in order to inform group-specific interventions that may improve uptake of cervical cancer screening programmes.  **Study Setting:** Two hospitals, one in the South West and the other in the North Central region of Nigeria  **Study design:** FGDs  **Study population**: 49 women | | **Type of analysis:** Qualitative Analysis – Content Analysis  **Validity (Internal):** FGD environment may have caused some participants  to give answers they perceive to be more ‘socially acceptable’, while more reticent participants may have  shied away from participation. There may also have been some selection bias in recruiting participants for the FGDs. Detailed thematic and reflective memos were kept by the authors, COREQ-32 checklist followed.  **Validity (External):** Participants in the study were relatively well educated and they may have provided responses that are not generalisable to the general population.  **Reliability (repetitiveness):** Not discussed. Sample size small. Perceptions of Muslim women in Ondo not covered. | **Approachability:** Muslim ladies felt discrimination at healthcare facilities because their mode of dressing readily identifies their faith.  lack of confidence in the healthcare system, confidentiality of results,  **Acceptability:** need for spousal approval, denial of disease condition  All FGD participants identified cultural norms of modesty as a barrier to seeking cervical cancer screening; Muslim FGDs were more emphatic about this barrier  Gender of healthcare providers was a major barrier  **Affordability:** financial limitations - will participate in a screening Programme if the screening test provided was free  **Appropriateness:**  self-sampling - Participants expressed concerns about being able to take adequate samples and would rather have the samples taken by a trained provider in a healthcare facility  **Awareness:** Beliefs of wizardry among Christians that cervical cancer may be caused by charms deployed by men who are unhappy with their female sexual partners  Belief that inserting herbs into the vagina cause cervical cancer,  None of the Muslim participants in the FGD conducted in South Western Nigeria had ever heard about cervical cancer, most did not understand the part of the body affected by it or where the cervix is located.  Lack of awareness of screening programmes  **Angst (Fear):** Fear of disclosure of results, fear of contracting other illnesses (fear of nosocomial infections) in the hospitals, | | | | | | | **Conclusion**  Barriers to cervical cancer screening vary by religious affiliations. When compared with the Christian FGD participants, the Muslim women were less likely to be aware of cervical cancer  A higher prevalence of reluctance to engage with the healthcare system among Muslim participants.  **Policy Recommendations**  Interventions to increase cervical cancer awareness and screening uptake in multicultural and multireligious communities need to take into consideration the varying cultural and religious beliefs in order to design and implement effective cervical cancer screening intervention programmes.  Methods of cervical cancer prevention in this study, additional modalities for dissemination of health information including use of text messages and campaigns in public spaces including mass transport vehicles should be actively explored | |
| **22** | Mahalakshmi et al, 2020, Barriers to Cancer Screening Uptake in Women: A Qualitative Study from Tamil Nadu, India, Journal article, India | **Objective:** To understand the barriers and enablers among community women would aid in increasing the proportion of cancer screening uptake  **Study Setting:** Not specified  **Study design:** Descriptive qualitative study using in-depth semi-structured interviews  **Study population:** Nineteen key informants including community women, service providers and a cancer survivor | | **Type of analysis:** Manual descriptive thematic analysis using deductive approach  **Validity (Internal):** The study population was small and there was not enough representation from each category. Triangulation of data.  **Validity (External):**  As the study was conducted in a specific Indian region, generalizability is difficult.  **Reliability (repetitiveness):** Not discussed but Triangulation of data. | **Acceptability:**  cultural beliefs, it was also associated with fear of discrimination by family and society, stigma related to cancer  Many held the belief that being asymptomatic, feeling fit or healthy - does not necessitate screening  Embarrassment - of revealing their body parts  Lack of family support, spousal support  Procrastination and Carelessness – barriers influenced by social convictions  Screening conducted by male physician  **Availability:** Screening was inaccessible to those in villages due to inadequate facilities.  **Affordability:**  Financial difficulties – most found screening cost to be high,  opportunity cost of daily wages  **Appropriateness:**  Pervasive distrust on the health care facilities,  doctors from government hospitals were unavailable or unreceptive,  **Awareness:** Lack of awareness  Lack of understanding about cancer (that cancer is contagious).  **Angst (Fear):** Fear of screening procedure,  Many participants reported that fear of being diagnosed with cancer (that cancer is equivalent to death) was a major barrier to screening uptake.  Cancer screening was frequently described as a painful and uncomfortable process | | | | | | | **Conclusion**  This study reiterates the psychosocial barriers and enablers that have been prevalent in the community women over the period of time  **Policy Recommendations**  Incentivization to women attending cancer screening could improve the rate of screening uptake  Usage of decision aids will help in increasing the rate of acceptance in contemplating stage, thus improving the screening uptake | |
| **23** | Ndejjo et al, 2017, Knowledge, facilitators and barriers to cervical cancer screening among women in Uganda: a qualitative study, Journal article, Uganda | **Objective:** To explore community knowledge, facilitators and barriers to cervical cancer screening among women in rural Uganda so as to generate data to inform interventions.  **Study Setting:** Eastern Uganda in two majorly rural districts of Bugiri and Mayuge  **Study design:** A qualitative study using focus group discussions and key informant interviews  **Study population:** 119 screening-eligible women aged between 25 and 49 years and 11 healthcare providers and administrators. | | **Type of analysis:** Qualitative Analysis – Directed content analysis  **Validity (Internal):** FGD environment could have  influenced participants to give answers that they perceive to be more socially acceptable.  Conducted by experienced qualitative researchers. Transcripts verified by all researchers.  **Validity (External):** Although the study was carried out in two majorly rural districts and results not be generalisable to  the whole country  **Reliability (repetitiveness):** Women and key informants interviewed using FGDs  and KIIs respectively that allowed for varied responses and enabled triangulation of findings. High reliability. | **Acceptability:** uncomfortable undressing themselves before health workers  **Availability:** Lack of health facilities offering screening, services being far away from the community  A**ffordability:** financial constraints, lack of finances to cater for transport to visit screening centres,  screening costs especially when accessing service from private providers and treatment costs if found to have the disease  **Appropriateness:** inadequate human resources, lack of proper training to carry out screening and lack of screening materials at health facilities  the mistreatment of women by health workers at health facilities.  Not comfortable with the way they are handled by health workers,  **Awareness:** Study participants’ knowledge about cervical cancer causes, signs and symptoms, testing methods and prevention was poor.  Many participants attributed the cause of cervical cancer to use of contraception while key informants said that some believed it was due to witchcraft. Several misconceptions existed among participants  Knowledge about methods used for cervical cancer screening was poor with many participants stating that they had never heard of any method  Lack of awareness of services availability,  **Angst (Fear):** fear of test outcome and consequences, | | | | | | | **Conclusion** Whereas perceptions towards cervical cancer and screening were positive, knowledge of study participants on cervical cancer was poor.  **Policy Recommendations**  To publicize screening services to increase their uptake including through use of mass media such as radio and television.  Building the capacity of available female health workers some of whom may be of lower cadres such as nurses and midwives through task shifting to carry out cervical cancer screening of women with support from other staff. | |
| **24** | Pryor et al, 2017, Cervical cancer screening in rural mountainous Honduras: knowledge, attitudes and barriers, Journal article, Honduras | **Objective:** To assess knowledge, attitudes and barriers to cervical cancer screening services by Papanicolaou smear (pap smear) for women in rural, remote Honduras  **Study Setting:** La Hicaca, Yoro and the 17 surrounding villages.  **Study design:** 20 question survey  **Study population:** 146 | | **Type of analysis:** Quantitative analysis – Descriptive analysis, Chi-square, Fisher’s exact tests & student’s t-test.  **Validity (Internal):** The screening rate among  this sample may have been artificially high due to selection bias, as only women who came to the clinic were surveyed. These women may be more likely to be screened for cervical  cancer. Another limitation may be recall bias, because women may not remember exactly when they were last screened, especially if the last screen was many years prior to the survey.  **Validity (External):** While women were invited to complete the  questionnaire after clinic, it is possible that they felt coerced into completing the questionnaire. The results may thus not be generalized.  **Reliability (repetitiveness):** Not discussed | **Acceptability:** Belief that getting screened wouldn’t make a difference (five respondents).  **Availability:** Inconvenience of having to travel a great distance (six respondents)  **Appropriateness:** More than one-third of the women who had undergone cervical cancer screening in the survey population did not know the results of their pap smears  **Awareness:** Of the respondents with no prior cervical cancer screening, nearly three-quarters (40/54 respondents, 74%) had never heard of a pap smear.  **Angst (Fear):** The most common barrier was fear of being screened (15 respondents) | | | | | | | **Conclusion**  These survey findings raise new questions about the optimal strategy for consistently performing cervical cancer screening in resource poor settings.  **Policy Recommendations**  Strategies to educate Honduran women on understanding the benefits of pap screening and minimization of structural barriers to screening access are needed.  Mobile clinics where screen-and-treat methods could be performed as a possible solution to poor access | |
| **25** | Yang et al, 2019, Barriers to cervical cancer screening among rural women in eastern China: a qualitative study, Journal article, China | **Objective:** To explore barriers to free cervical cancer screening among rural women in China from the perspective of women, healthcare providers and women’s husbands to inform intervention planning  **Study Setting**: Two counties in Jining Prefecture of eastern China  **Study design:** Semi-structured in-depth interviews  **Study population**: 21 women, 14 healthcare providers & 4 husbands of women eligible for screening | | **Type of analysis:** Qualitative Analysis – Thematic Analysis  **Validity (Internal)**: Purposeful sample over-represent women with lower educational levels  Four trained interviewers conducted interview. Triangulation of data. Data validity in translation.  Peer effect in focus group discussions may have influenced providers to  give answers that they perceive to be more socially acceptable.  **Validity (External**): Findings relate primarily  to small study setting and not generalisable  **Reliability (repetitiveness):** Data collected over a short period of time. Triangulation of data. High reliability. | **Acceptability:** cultural barriers including reticence for intimate Examinations - reluctance to remove clothing or allow genital exam- especially being exposed in front of non-family members.  Influence of close contacts on screening decisions - if close contacts were not willing to participate, this reduced women’s acceptance of screening.  Nearly half indicated that they would not undertake screening if performed by a male doctor  **Affordability:** cost of treatment - women with fewer economic resources reported avoiding screening because they were worried they would not have the money for treatment if they were diagnosed with the cancer, noting that at present, while screening is free of charge, treatment is not.  Time needed for screening - Difficulty in scheduling or otherwise allocating time for screening  **Awareness**: Gaps in knowledge of cervical cancer and health awareness - none knew HPV infection is the cause, misconception that cervical cancer will never happen after menopause, limited knowledge of the process, cycle, purpose and importance of screening  **Angst (Fear):** fear of cancer and screening outcomes – Majority of women in the interviews expressed a deep fear of cancer. did not want to know if they had cervical cancer, wanted to avoid the anxiety and distress of a cancer diagnosis | | | | | | | **Conclusion**  Study details important barriers to cervical cancer screening in terms of gaps in knowledge, fear of cancer and screening outcomes, cultural embarrassment, the influence of close contacts and inconvenience.  **Policy Recommendations**  Education initiatives will need to address whole communities and possibly include peer-education approaches that encourage positive health discussions among friends  New methods for screening using automated nucleic acid amplification tests may help overcome timing and location constraints by allowing more rapid screening, and may help overcome cultural distaste for vaginal examinations by allowing women to self-collect vaginal swab samples. | |
| **26** | Matenge et al, 2018, Barriers to accessing cervical cancer screening among HIV positive women in Kgatleng district, Botswana: A qualitative study, Journal article, Botswana | **Objective:** To explore the barriers to women with HIV accessing cervical cancer screening in Kgatleng district, Botswana  **Study Setting**: Oodi village in the Kgatleng district of Botswana  **Study design:** Phenomenological qualitative study utilizing semi-structured interviews  **Study population**: 14 HIV positive women | | **Type of analysis:** Qualitative Analysis – Framework method  **Validity (Internal):** Since it had a single researcher, interviews were conducted in a consistent manner, in the women's home language and translation was accurate. Analytical process was weak  As the second researcher was merely a supervisor and no separate and independent  Analysis done. Being interviewed by a medical doctor could have introduced an obsequiousness bias. Systematic analysis using Atlas-ti software.  **Validity (External):** The sampling included more people that had undergone cervical screening than had not  and uneven numbers between age categories, which could potentially influence the range of  themes identified. Findings were only derived from people in Kgatleng district  **Reliability (repetitiveness):** Not discussed. Medium reliability | **Approachability**: Long queues and waiting times,  Clinic procedures were inconsistent with some operating without appointments and others requiring them  Healthcare workers might instruct women to have a cervical smear without explaining why this was necessary.  **Acceptability:** Patients did not give the same level of importance to attending for a cervical smear as they gave to attend HIV clinic,  Embarrassed undressing in front of the nurses,  **Availability:** distance from the clinic and the unpredictability of public transport - limited access  **Affordability:** Women that were employed complained that it was difficult to take time off work as they would have deductions from their pay if they were absent to attend the clinic.  **Appropriateness:** Many participants never received their result or only obtained it months or years later.  Were asked to repeat the test, with no explanation offered as to why  Not having all the equipment to perform the cervical smear.  Others were told their results were abnormal, but could not repeat the test due to equipment shortages,  Shortage of nurses  Healthcare workers might not fully explain the significance of an abnormal result.  **Awareness:**  General lack of knowledge about screening and cervical cancer,  Lack of knowledge on the risk factors for cervical cancer,  Lack of knowledge on the recommended frequency of testing was widespread,  Not aware that cervical cancer was preventable,  Not being aware that there is treatment for cervical cancer precursor lesions  **Angst (Fear):** Fear of pain of screening procedure,  Fear that results might reveal a life threatening or incurable disease | | | | | | | **Conclusion**  Although the participants heard that screening was important, they did not understand why and could not link it to prevention of cervical cancer. Participants had low levels of confidence with regard to the quality of the service due to poor patient-centeredness, inadequate appointment systems, unavailability of equipment and results. Moreover, there were many fears and misunderstandings regarding the screening process and interpretation of results  **Policy Recommendations**  Integrating cervical screening with visits for other problems, such as HIV management, would improve utilization of cervical cancer screening.  Telephonic communication should enable the scheduling and re-scheduling of appointments and text messaging to inform women about the availability of results or to recall them.  Health education and promotion should be intensified to raise awareness and understanding of cervical cancer screening using the general media, social media, community talks, clinic pamphlets or posters. | |
| **27** | Darj et al, 2019, Barriers and facilitators to cervical cancer screening in Nepal: A qualitative study, Journal article, Nepal | **Objective**: To investigate and better understand Nepali women’s perceptions on barriers to participation in cervical cancer screening and what might facilitate their participation  **Study Setting:** Dhulikhel Hospital and in three communities in Dhulikhel Municipality, 30 km east of the Kathmandu  **Study design:** Qualitative study with FGDs  **Study population**:72 women aged between 25-60 years | | **Type of analysis:** Qualitative Analysis – Manifest Content analysis  **Validity (Internal):** Some of the women might have agreed with the most influential participant’s view and provided expected socially desirable comments. First author Nepali oncological nurse – better understanding of participants’ situation. Other 2 authors gynecologists. Systematic analysis done by all 3 authors. Transcriptions were repeatedly read, discussed, and supported by quotes.  **Validity (External):** Women had similar ethnicities, spoke the same language and therefore no information from other ethnic groups. Health care providers’ and men’s perspectives were not included in the study  **Reliability (repetitiveness):** Data collected over a short period of time, all seven FGDs were conducted by  the same FGD moderator and research assistant, High reliability. | **Approachability:** seemed to lack trust in health care providers due to experiences of inappropriate behavior  opinion that a service provider’s negligence or incompetence would cause their health to deteriorate and cause complications  **Acceptability:** Women sought health care only when physical symptoms appeared  Will undergo screening only after Approval from spouse  No time due to family priorities  Felt ashamed to show their genitals to others, especially male doctors  **Availability:** Village health posts lacked screening facilities.  **Affordability:** concerned about incurring unexpected costs if additional investigations were necessary, and they discussed having to face impossibly high costs if surgery was needed  **Awareness:** Participants had never heard of cervical cancer.  No awareness of HPV screening, as a means of early detection of precancerous stages and possible preventive treatment to avoid the development into cancer:  Wrong assumptions - cervical screening can detect uterine complications, negative screening result means one can never catch cervical cancer  **Angst (Fear):** Anxiety and fear about the results  Fear of gossip by community members | | | | | | | **Conclusion**  The barriers to participation were due to misunderstanding and lack of knowledge, sociocultural norms, distrust of health providers, financial limitations, and challenging physical geography.  **Policy Recommendations**  Recommend the provision of affordable, accessible, and women-friendly cervical cancer screening programmes for women in Nepal according to their geographical location.  A creatively tailored ‘mass awareness’ Programme is required to address the revealed misconceptions about cervical cancer screening. | |
| **28** | Ilaboya et al, 2018, Perceived barriers to early detection of breast cancer in Wakiso District, Uganda, using a socioecological approach, Journal article, Uganda | **Objective:** To investigate the perceived barriers to early detection of breast cancer in Wakiso district, Uganda  **Study Setting:** Ssisa sub-county, Wakiso district in the central region of Uganda  **Study design:** Multilevel approach (FGDs + Key informant interviews) focused through a socioecological framework  **Study population:** 24 | | **Type of analysis:** Qualitative Analysis – Transcription, Thematic analysis & familiarization  **Validity (Internal):** Pilot study conducted, triangulation of data, audit trail of the research process  **Validity (External):** This study involved only one sub-county, therefore the  results may not necessarily be generalizable.  **Reliability (repetitiveness):** triangulation and transferability of data. High reliability. | **Acceptability:** Apathy was highlighted as a major attitude most women exhibit  Only seek medical help when they feel pain.  Breast cancer detection activities would add no ‘financial’ value to them.  Competing health burden is also reflected in the low prioritization of NCDs by the patients  **Availability**: lack of cancer services at the PHC level  **Appropriateness:** Health facilities in Wakiso district were also reported as not equipped to address diseases such as breast cancer.  PHC level health workers had no training whatsoever on breast cancer or other non-communicable diseases  **Awareness:** Little or no knowledge was identified as the most significant factor that may prevent a woman from detecting breast cancer early  Lack of mass awareness  **Angst (Fear):** Fear of death, fear of big hospitals, fear of mastectomy and fear of the unknown. | | | | | | | **Conclusion**  Findings are consistent with existing literature that barriers to breast cancer detection in SSA are attributed to a number of complex but interacting factors including low knowledge, beliefs and inaccessibility to health facilities, and weak healthcare systems  **Policy Recommendations**  Need for a cancer policy for Uganda that prioritizes early detection  Particular focus on strengthening PHC capacity to promote and ensure universal coverage in terms of breast awareness | |
| **29** | Moucheraud et al, 2020, “It is big because it’s ruining the lives of many people in Malawi”: Women’s attitudes and beliefs about cervical cancer, Journal article, Malawi | **Objective:** To perform a multi-level assessment of Malawian women’s knowledge and perceptions of cervical cancer risk and screening  **Study Setting:** 3 health facilities in Central Malawi  **Study design:** Qualitative Interviews using Multi-Level Health Outcomes Framework  **Study population**: 60 adult Malawian women aged 18-62 | | **Type of analysis:** Qualitative Analysis – Transcription, translation & iterative coding  **Validity (Internal):** Responses subjected to reporting bias including recall bias and social desirability bias.  **Validity (External):** Participants recruited from ‘screen and treat’ facilities. Not generalisable to places without the services. Sampling strategy resulted in a respondent pool of above-average HIV prevalence. Did not collect sociodemographic  information on respondents  **Reliability (repetitiveness):**  Not discussed. | **Approachability**: long waiting times  **Acceptability:** Issues of modesty  Husbands not supportive  **Availability:** distance to screening Centre  **Affordability**: cost of transportation  **Appropriateness:** supply shortages  ill treatment by health care providers - rude doctors  **Awareness:** rumors that lesion removal leads to infertility  **Angst (Fear):** Fear of pain of screening a deterrence | | | | | | | **Conclusion**  Reflecting the high prevalence of invasive cervical cancer in Malawi, women who were interviewed for this study saw cervical cancer as dangerous and common – and many had lost friends or family members to cervical cancer. Knowledge of the disease was universal, and women were well-informed about its source as a sexually transmitted virus.  **Policy Recommendations**  Interventions to improve screening coverage have largely focused on increasing knowledge and expanding service availability. Cancer control researchers, practitioners, and policymakers should augment these efforts with interventions targeting other factors that may play a crucial role for women, such as engaging social networks and educating and including spouses/partners in the screening process. | |
| **30** | Mahajan et al, 2019, Study of Knowledge, Attitudes, and Practices Toward Risk Factors and Early Detection of Noncommunicable Diseases Among Rural Women in India, Journal article, India | **Objective:** To assess the knowledge, attitudes, and practices of rural women in low-resourced countries toward common NCDs and the barriers they face in receiving NCD early detection services  **Study Setting**: 50 villages of Gogunda rural block, Udaipur district, Rajasthan, India  **Study design:** Rapid Assessment and Response Evaluation ethnographic assessment including in-depth interviews, FGDs & Survey  **Study population:**  1192 women for survey 16 women, 15 men, 2 community health workers, a school teacher, 2 civil volunteers for the FGs and An official from the Ministry of Health, local elected people’s representative, local GP, a local specialist from the secondary health facility and a representative from the local civil society organization for the KII | | **Type of analysis:** Qualitative Analysis – Thematic Analysis  **Validity (Internal):** Multiple methods of data collection  **Validity (External):** The population was  highly selective; thus, the results are not  generalizable.  **Reliability (repetitiveness):** Multiple methods of data collection. High reliability. | **Approachability:** No sustained effort to create awareness about the NCD clinic.  **Acceptability:** Too ashamed to discuss any female health problems with men.  Prefer to consult the village faith healer rather than seek help from their husbands or other male family members  Women needed permission from their husband or father-in-law to visit the physician. They had to be accompanied by husbands or other males.  **Availability:** The only hospital with comprehensive cancer treatment facilities was situated in the capital city, and it took the villagers almost a full day to travel.  **Appropriateness:** lack of facilities at the PHC. Poor infrastructure, low quality of services, inadequate manpower  **Awareness:** Cancer was associated with death and seen as a divine punishment for sins committed in a past life.  Misconception that cancer is a contagious disease | | | | | | | **Conclusion**  Study of rural indigent women who reside in a hard-to-reach area of India highlights the major barriers to accessing NCD control services, which are low empowerment of women, poor availability of services, a low level of awareness about NCDs, and a lack of a preventive health orientation.  **Policy Recommendations**  Rural CHWs need to be trained and empowered to raise awareness in the community  A culturally appropriate and broad-based communication strategy to educate the community will help to overcome some of the identified barriers. The low social empowerment of women needs to be addressed. | |
| **31** | Tillyard et al, 2019, A mixed method, community‐based investigation on women’s cancer awareness in Haiti, Journal article, Haiti | **Objective**: To understand perceptions and attitudes towards breast and cervical cancer in Haiti through community‐engaged research  **Study Setting:** 4 of Haiti’s 10 geographical departments - north west, central plateau, west & south  **Study design:** mixed quantitative and qualitative surveys and focus groups  **Study population:** 414 participants aged 13-65, 75% of which were women for the survey on women’s knowledge of cancer, a separate smaller survey of 50 breast cancer patients and survivors | | **Type of analysis:** Quantitative Analysis – ANOVA, Qualitative Analysis using NVivo  **Validity (Internal):** Small sample size, lack of random selection of participants  **Validity (External):** Representative data of all regions in Haiti  **Reliability (repetitiveness):** Mixed method study design, medium reliability | **Awareness:** Low levels of knowledge about cancer across all geographic regions of the country,  About contracting breast cancer, only 13/91 or 14% of responses could be considered accurate or consistent with current medical knowledge.  About diagnosing cervical cancer, only 12% reported to know what a test for cervical cancer would be.  **Angst (Fear):** Fear of violence and discrimination | | | | | | | **Conclusion**  Results demonstrate low cancer knowledge of women's cancer across all segments of Haitian society.  **Policy Recommendations**  To design awareness materials that specifically target the largest gaps in knowledge | |
| **32** | Kilic et al, 2019, Breast and cervical cancer screening for women with physical disabilities: A qualitative study of experiences and barriers, Journal article, Turkey | **Objective:** To determine the experiences of women with physical disabilities regarding the barriers to their participation in breast and cervical cancer screening  **Study Setting:**  **Study design:**  Qualitative descriptive study.  **Study population:** Sixteen women who use wheelchairs | | **Type of analysis:** Qualitative Analysis - Thematic Analysis  **Validity (Internal):** Two researchers individually examined the data, analysed the results, and established the topics to guarantee credibility and dependability. COREQ checklist used. Face-to-face interviews, conducted by trained professionals  **Validity (External):** Participants had similar income levels and they perceived their income status to be at a low or medium level, findings  may not be representative of the experiences of most women with disabilities at a high-income level.  **Reliability (repetitiveness):** Criteria of credibility, dependability, confirmability,  and transferability. High reliability. | **Approachability:** participation of women who use wheelchairs in mammography and Pap smear screening was influenced by personal factors and the information provided by healthcare providers.  Not able to move inside the examination rooms with their wheelchairs.  Healthcare personnel often did not know exactly how to assist a disabled person  Disabled patients felt healthcare personnel had more power  Hospital bathrooms were not disability friendly  **Acceptability:** Seven of the women said that they did not get a Pap smear test because they were embarrassed to get it done  “Embarrassed” during the examination  Seven of the women stated that the gynecological examination table and the mammography device were not suitable for disabled individuals  14 people stated that they required assistance in both travelling to the hospital and in undergoing hospital procedures.  **Availability:** difficulties in accessing public transportation,  absence of paths suitable for a wheelchair/vehicle  **Appropriateness**: difficulty reaching the exam table:  **Awareness:** Lack of knowledge/neglect, not knowing how/where the screening would take place  **Angst (Fear):** Five of the women in the study said that they had not had a mammogram or Pap smear screening because they feared a bad outcome. | | | | | | | **Conclusion**  The participation rate of women with physical disabilities in screening is low. The physical barriers that women with disabilities face are also barriers to the protection and maintenance of their health and are often disregarded when providing patients with information and education about cancer screening and access to services.  **Policy Recommendations**  This study suggests that the participation of women who use wheelchairs in screening would increase if the following conditions were to be met: easier access to hospitals and healthcare centers, fewer physical obstacles inside hospitals and examination rooms, less time consuming examinations and the assistance of health‐care personnel trained to work with those with disabilities. | |
| **33** | Chaka et al, 2018, A survey of knowledge and attitudes relating to cervical and breast cancer among women in Ethiopia, Journal article, Ethiopia | **Objective:** To investigate knowledge and attitudes related to breast & cervical cancers among women in 4 zones of Ethiopia  **Study Setting:** North Shewa zone (Amhara region), Gamo Gofa zone (Southern Nations, Nationalities and Peoples’ region) and zones 1 and 3 (Afar region) of Ethiopia  **Study design:** Community based cross-sectional study  **Study population:** 799 women aged 18 & older | | **Type of analysis:**  Quantitative Analysis - Multiple logistic regression analysis  **Validity (Internal):** Pilot study conducted, questionnaire in both English and Amharic. Trained interviewers administered the questionnaire.  **Validity (External):** Multistage cluster sampling technique. Proportional stratified sample was drawn from each respective regional zone  **Reliability (repetitiveness):** The  Cronbach’s alpha showed acceptable reliability for the group of questions pertaining to attitude of cervical cancer  (∝= 0.75) and breast cancer (∝=0.77). | **Acceptability:** The majority of the women have negative attitudes towards cervical cancer (70.6%).  The majority of the interviewers also preferred female health worker to conduct cervical examination (70%).  A belief that they were not at risk for developing cervical cancer, 40% felt breast exams are embarrassing  **Availability:** 44% said they did not have a screening center close to where they live  **Affordability:** A third did not want to undergo breast exams if they had to pay for it.  53% felt breast exams take too much time  **Appropriateness:** 27% felt health workers were rude to women.  **Awareness:** Fewer than half (42.2%) of the study participants had ever heard of cervical cancer.  Less than half gave the appropriate response to questions related to risk factors for cervical cancer.  Only 21.4% knew that human papilloma virus (HPV) is a risk factor for cervical cancer  Almost two-thirds (63%) of the women interviewed had ever heard of breast cancer.  Among those who had heard of breast cancer (503/799), only 21.3% had heard of breast cancer screening  **Angst (Fear):** 70.3% among those who had heard of cervical cancer categorized it as “scary”.  Over a third (37.8%) responded that “breast cancer would threaten a relationship with her husband, boyfriend or partner”.  fear of pain during the procedure  as well as the fear of being diagnosed with cancer and dying shortly thereafter.  50% felt breast exams are painful | | | | | | | **Conclusion**  Study found that the overall knowledge of risk factors for breast cancer and cervical cancer among women was low  **Policy Recommendations**  There is an urgent necessity to inform Ethiopian women about cervical and breast cancer screenings. Efforts to promote knowledge on risk factors of female cancers should reach all women, as well as men, and provide health education and community-based interventions. | |
| **34** | Olasehinde et al, 2017, Developing a Breast Cancer Screening Program in Nigeria: Evaluating Current Practices, Perceptions, and Possible Barriers, Journal article, Nigeria | **Objective:** To evaluate the perceptions, practices, and barriers regarding clinical breast examination (CBE) screening in a low- income community in Nigeria.  **Study Setting:** Ife Central local government area of Osun State, southwestern Nigeria  **Study design:** cross-sectional survey  **Study population:** 1169 women aged 40-86 years | | **Type of analysis:** Simple and multivariate Poisson regression  **Validity (Internal):** Questionnaire in both languages – English and Yoruba. Pretesting done.  **Validity (External):** Homogeneity of the sampled population in terms of culture, not generalizable. Only one state – Osun.  **Reliability (repetitiveness):** Not discussed. Medium reliability. | **Approachability:** Only 37.7% of the women had been recommended breast screening by health practitioners. Only 37.7% of the respondents had ever had breast cancer screening recommended to them.  **Acceptability:** Only 230 women (19.7%) had ever had their breasts examined by a health practitioner.  Mammography screening had only been done in 33 women (2.8%).  Seven hundred sixty-five respondents (65.4%) were willing to participate in a regular breast examination screening program, whereas 404 respondents (34.6%) were not.  Lack of perceived need was the reason given for non-willingness by those who declined.  **Awareness:** Only 6% had not heard about Breast cancer | | | | | | | **Conclusion:**  CBE practice, although poor, is acceptable to the majority of women in the studied population with few social concerns.  **Policy Recommendations** Creating awareness with educational programs is needed to correct erroneous perceptions about breast cancer and the need for screening. Patient navigation may be of benefit in LMICs. Opportunity for trained health personnel to provide breast cancer advocacy and screening to women who visit hospitals for various health challenges | |
| 35 | Narayana G et al, 2017, Knowledge, attitude, and practice toward cervical cancer among women attending Obstetrics and Gynecology Department: A cross‑sectional, hospital‑based survey in South India, Journal Article, India | **Objective**: To assess the level of knowledge awareness and practices of women towards cervical cancer screening and measure its association with sociodemographic factors  **Study Setting**: Hospital‑based cross‑sectional study which was conducted in Outpatient Department of Obstetrics and Gynecology of secondary care referral Hospital in South India,  **Study design**: Cross sectional, quantitative survey interview  **Study population**: 403 women aged over 18 years | **Type of analysis**: Quantitative, chi squared test  **Validity (Internal)**: The survey questionnaire used was prevalidated. The interview methods validation has not been discussed  **Validity (External)**: Findings not generalizable as the study population were limited to those visiting the outpatient clinics in the hospital  **Reliability (repetitiveness)**: Underreporting and misclassification likely due to self-reporting of prior cervical screening | | **Affordability:** 44 (10.9%) Strongly agreed, 75 (18.6%) Agreed, 75 (18.6%) Neither agreed nor disagreed 122 (30.3%) Disagreed, 87 (21.6%) Strongly disagreed that Screening for premalignant cervical lesions is not expensive  **Awareness:** 71 respondents (17.6%) strongly agreed, 65 (16.1%) agreed, and 57 (14.1%) neither agreed nor disagreed that cervical cancer is highly prevalent and leading cause of death among all cancers in India.  76.9%, 301 women were aware of cervical cancer screening methods.  47 (11.6%) Strongly agreed, 65 (16.1%) Agreed, 76 (18.8%) Neither agreed nor disagreed 103 (25.5%) Disagreed, 112 (27.8%) Strongly disagreed that screening helps in prevention of cervical cancer  However, (349; 86.6%) were never screened for cervical cancer  300 (46.3) were aware about the location of screening | | | | | | | **Conclusion:**  Women have good knowledge, positive attitude toward cervical cancer screening, and prevention; still there is a gap to transform it into practice. High knowledge and positive attitudes themselves are not enough to ensure uptake of practice of screening.  **Policy Recommendations**  Planned communication aiming eligible women, complete availability of screening services in public health facilities may increase the acceptance of screening. There is a need for more educational programs to channel identified knowledge slits and scale up of regular practice of cervical cancer screening in women.  The study found an association between sociodemographic factors and the knowledge, attitude and practice of women towards cervical cancer screening. In order to improve uptake of cervical cancer screening, the sociodemographic factors will have to be addressed | |
| 36 | Shashi Prateek et al, 2018, Knowledge and attitude of women attending Subharti Medical College towards Pap smear, Journal Article, India | **Objective**:  To assess the knowledge and attitude of women towards Pap smear  **Study Setting:** Department of Obstetrics and Gynaecology Subharti Medical College  **Study design:** Pre-formed questionnaire  **Study population:**  N= 150 women of age group 21-75 years attending the outpatient clinic and admitted in the Department of Obstetrics and Gynaecology | **Type of analysis**: Graphs & charts  **Validity (Internal)**: No pre-validation of questionnaire, single participant group.  **Validity (External)**: Findings not generalizable as the study population were limited to those visiting the outpatient clinics in the hospital  **Reliability (repetitiveness)**: Not discussed. | | **Approachability:** Thirty-seven percent of the women believed that if the Pap test is required, then their doctors will prescribe it to them.  None of the women got information from an ASHA worker, dai or any other paramedical worker in their area  **Acceptability:** 35% of the women never got it done for various reasons, like embarrassment and shame in explaining the test to their family members.  Fifty-nine percent of the women firmly believed that they could never have cervical cancer.  **Awareness:** Sixty-seven percent (100 women) women had no idea about the Pap smear and only 33% (50 women) were aware about it.  Of these, 50 women who were aware about the Pap smear, 40% (20 out of 50 women) received their information from a doctor.  Of these, 50 women who knew about the Pap smear, 45 knew that pap smear can detect cancer but none of them were aware that a Pap smear can diagnose pre-cancerous lesions  Only nine percent of the women knew that a Pap smear needs to be performed at regular intervals of three years.  10 women believed the Pap smear was a useful test for the treatment of cancer  Fifty-nine women (39.3%) believed that only women with a family history of cancer could develop cervical cancer  Twenty-six percent of the women believed that it is a disease of extreme old age.  13% were totally unaware about cervical cancer  **Angst (Fear):** fear of having a painful test, | | | | | | | **Conclusion:** In India, where cervical cancer is the second most common cause of cancer deaths, even our educated, urbanized women are unaware about the Pap smear and cervical cancer. Unless we improve the awareness among women about the importance and need for a Pap smear, we will lose this battle against cervical cancer.  **Policy Recommendations**  None | |
| 37 | JR Gordon et al, 2019, “HIV is not an easily acceptable disease”: the role of HIV related stigma in obtaining cervical cancer screening in India, Journal Article, India | **Objective**: To examine the relation of HIV-related stigma to obtaining CC screening among women  **Study Setting**:  New Civil Hospital (NCH) ART Centre  in Surat, India  **Study design**: Qualitative, Semi-structured individual in-depth interviews  **Study population**: Women aged 18 years’ or older  25 women receiving care at the New Civil Hospital (NCH) ART Centre (patients) and 15 health-care workers providing care to at NCH (stakeholders) | **Type of analysis**: Qualitative - thematic analysis  **Validity (Internal)**: Interviewers were provided guides and prompts to maintain uniformity and were trained to perform the interviews.  It was done by trained interviewers  The thematic coding underwent rigorous validation process.  **Validity (External)**: Findings not generalizable as the study population were limited to those visiting the outpatient clinics and cultural variances may precipitate stigma in other geographic locations  **Reliability (repetitiveness)**:  Good reliability - The thematic coding underwent rigorous testing and verification process. | | **Approachability:** Stigma and discrimination by healthcare workers in healthcare settings against patients living with HIV hamper their access to cervical cancer screening  Involuntary disclosure of HIV status by the healthcare team discouraged women living with HIV from seeking cervical cancer screening services. | | | | | | | **Conclusion:**  Stigma and discrimination can hinder HIV positive women in India from obtaining recommended cervical cancer screening**.**  **Policy Recommendations**  Future research should use quantitative methods to evaluate the role of HIV disclosure and HIV-related stigma in obtaining recommended Cervical cancer screening in India and should develop interventions to increase access to Cervical cancer screening in consideration of the experience of HIV stigma.  The study highlights is existence of stigma in the society and amongst healthcare workers against patients living with HIV. This inherent bias and discrimination prevents women from accessing or utilizing cervical cancer screening services. There is a need for structural reform to eliminate the taboo surrounding HIV. | |
| 38 | Abdikarim IK, Carole Atieno WM, Habtu M, 2017, Prevalence and associated factors of cervical cancer screening among Somali women in an urban settlement in Kenya, Journal Article, Kenya | **Objective**: To determine prevalence and associated factors of cervical cancer screening among Somali women in Eastleigh, Nairobi, Kenya.  **Study Setting:** Eastleigh, a suburb of Nairobi  **Study design:** A cross-sectional study  **Study population:** 104 Somali women aged between 25-45 | **Type of analysis:** Quantitative analysis - Descriptive analysis, chi-square test, multivariate logistic regression  **Validity (Internal):** The use of self-report which may be affected by the tendency of interviewees to give socially desirable response. Questionnaire translated to Somali and Kiswahili and back-translated to English. Face-to-face interviews. Information collected by trained research assistants.  **Validity (External):** Multi-stage random sampling utilized. The study was conducted using cross sectional study in the one community dominated by Somali population, which may not be generalizable to other areas or represent the whole nation  **Reliability (repetitiveness):** Not discussed. Data collected over short time period. Strong analytical technique. | | **Acceptability:** More than half (55.8%) claimed that doing pap smear test is unpleasant and embarrassing.  Majority (86.5%) preferred to be examined by female health providers.  (62.5%) were not aware of their perceived susceptibility to cervical cancer.  **Awareness:** 53.8% indicated that they didn’t know the use of Pap smear test.  (53.8%) of the respondents indicated that the test can interfere with virginity.  **Angst (Fear):** Fifty-nine (56.7%) of the participants also indicated that they could not undergo the test due to fear of results. | | | | | | | **Conclusion:** Even though the women in the study were willing to seek medical assistance if experienced signs of cervical cancer, their lack of awareness about Pap smear test and lack of perceived susceptibility to cervical cancer were major hindrance to screening. These probably contributed to a low prevalence cervical screening of 32.7% compared to the national target of 75% by the year 2009.  **Policy Recommendations**  Specific awareness and perception target programs should be conducted to scale up cervical cancer screening in this community.  Targeted studies among other communities to compare the prevalence and factors associated with screening independently from the Somali community.  Community specific barriers to cervical screening could be identified for appropriate interventions to scale up cervical screening. | |
| 39 | RM. Kei et al, 2016, Challenges of Cervical Cancer Screening Among Women of Reproductive Age in Kisii Town, Kisii County, Kenya, Journal Article, Kenya | **Objective**: to assess the knowledge, practice and barriers towards screening for premalignant cervical lesions among women aged 15 years and above years in Kisii Town  **Study Setting**:  Kisii Town  **Study design**: Quantitative, interview based survey, Descriptive and cross-sectional  **Study population**: Women aged 18 years or older living in Kisii Town (Random sampling) | **Type of analysis**: Quantitative analysis – Chi-square test  **Validity (Internal)**: Interview pretested. Questionnaire administered by volunteer women. Systematic random sampling.  **Validity (External)**: Findings not generalizable as the study population were native to a particular location  **Reliability (repetitiveness)**: Not discussed. Medium reliability | | **Affordability:** (6.6%) reported that expensive tests were a barrier to screening  **Awareness:** (30.5%) were not aware of the importance of screening due to ignorance  55% of the respondents did not know the symptoms of cervical cancer  (63%) of the respondents did not know the risk factors for cervical cancer  (68.2%) of the respondents believed that cancer can be cured while (17.9%) said it could not be cured and (13.9%) did not know  (35.8%) had not heard about cervical cancer screening  (63.6%) did not know the screening methods  **Angst (Fear):** (13.4%) were afraid of the screening results  (7.5%) were scared of pain | | | | | | | **Conclusion:**  Limited knowledge about cervical cancer and low rate of screening for premalignant cervical lesion in Kenyan Women  Women’s reluctance to undergo cervical cancer screening appears to be based on lack of knowledge about the cervical cancer, the risk factors of cervical cancer and also lack of knowledge on the eligibility and availability of screening services  **Policy Recommendations**  More cancer screening machines should be purchased by County government and distributed to all town health facilities at a subsidized charge for screening  Women should be counselled before being screening and awareness should be intensified on the importance of screening in order to promote compliance. | |
| 40 | Morris RM, 2016, Factors associated with the uptake of cervical cancer screening among women of reproductive age in Homabay county, Kenya: A case of Kanyadhiang sub location, Journal article, Kenya. | **Objective:** To identify factors associated with cervical cancer screening among women of reproductive age in Kanyadhiang Sub location  **Study Setting:** Kanyadhiang sub location, Homabay county, Kenya  **Study design:** Descriptive cross-sectional study  **Study population:** Women of reproductive age (15-49 years), n=74 | **Type of analysis:**  Quantitative Analysis - Exploratory data analysis  **Validity (Internal):** Pre-tested questionnaire tool. descriptive cross-sectional.  **Validity (External):** Not discussed. Multi-stage sampling technique used.  **Reliability (repetitiveness):** Not discussed. | | **Approachability:** Only 12.16% received information about cervical cancer from community health providers and only 6.76% from community health workers  **Acceptability:**  4% said the religion doesn’t allow. 7% showed that they were uncertain on whether their religion allow or does not allow cervical cancer services.  Having no time since they are always committed at work, business place or household duties;  **Availability:**  Distance to the facilities - 68% of the respondents accessed the health facility from a radius of more than 5 km while only 32% accessed the same services within a radius of less than 5 km  **Affordability:** Lack of funds to pay for services, especially those that depend on their husbands  **Appropriateness:** 47% reported not to have received screening services yet they required them.  Some of the reasons given by those who missed services included;  Shortage of screening reagents;  shortage of trained staff on cervical cancer screening;  lack of clear screening programmes and policies within health facilities  **Awareness:** 74% of the women among those interviewed said they were aware of the cervical cancer screening services while 26% have never heard of the existence of screening services  Respondents never knew existence of such services in the local health facilities  Most of the respondents interviewed (59.46%) were not aware about the various methods used for cervical cancer screening. 8.11% of the women knew about VIA/VILI test, 16.22% knew about pap smear  **Angst (Fear):** Fear of the diagnostic outcome and fear of the medical procedures. 54 out of 74 women were worried about the outcome of the procedure | | | | | | | **Conclusion:**  Quite a good number only know that the services exist but only very few utilize the services  Multi-agency coordinated approach in promoting uptake of cervical cancer services.  **Policy Recommendations**  That the ministry of health and other relevant authorities to help sensitize and create awareness on the importance of cervical cancer screening  Religion leaders should help in encouraging the church members to participate in cervical cancer screening services.  Health care provider to be trained on cervical cancer screening skills and customer relations principles.  Ministry of health of Homabay County should employ more health staffs breach the shortage of health care providers.  Ministry of health should initiate community based outreach services in Kanyadhiang Sub location.  County government of Homabay should introduce community based educational program through local media to promote uptake of cervical cancer screening services. | |
| 41 | Gebru Z et al, 2016, Barriers to Cervical Cancer Screening in Arba Minch Town, Southern Ethiopia: A Qualitative Study, Journal Article, Ethiopia | **Objective**: to explore barriers to utilization of cervical cancer screening in Arba Minch town, Southern Ethiopia  **Study Setting**:  Arba Minch town, Southern Ethiopia  **Study design**: Quantitative, interview based survey  **Study population**: Women in Arba Minch town, Southern Ethiopia | **Type of analysis**:  Qualitative, IDI, thematic analyses  **Validity (Internal)**:  The interviewers were trained. Questionnaire was pretested in neighboring population. Details of training and validation not mentioned. In-depth interview conducted by trained facilitator.  **Validity (External)**:  Findings not generalizable as the study population were native to a particular location  **Reliability (repetitiveness)**:  Not discussed in detail but single source of data collection. | | **Approachability:**  **Acceptability:**  Some participants had the desire to visit health facilities as soon as the symptoms and signs appeared  Most of them felt that they did not believe that they were susceptible to cervical cancer, and that this might affect whether a woman felt it necessary to be screened  lack of understanding of the need to attend screening, hearing others’ negative stories about the test  perceived as a painful instrument and embarrassment  A few women talked about potential shame if diagnosed with cervical cancer as a barrier to attending screening among others.  cultural, socio-economic, and beliefs about the disease and the health care system were found to affect the treatment seeking behavior for cervical cancer  **Availability:** The barriers included; long distance to the facility and means of transportation to reach the health facility  **Affordability:** Some participants felt that the cost of treatment may be unaffordable  **Awareness:**  When asked about cervical cancer screening, women often had no concept of what the screening was for, what happened to a woman while she was being screened, or why it was important to be screened. Majority of them have not heard about any screening method  most of the participants stated that they have never heard about cervical cancer  Respondents were unsure who should be screened. Many believed that the Pap smear is only for married women  The limited knowledge was also associated with traditional beliefs among women. The fact that screening involves looking for disease in the absence of symptoms was seen as a reason not to be screened by one of the woman  **Angst (Fear):** Fear of the test was cited as an obstacle to some women, even if they appreciated the need for screening  Cancer was not only described as being rare, but also as stigmatized.  Few women remarked that cancer evoked secrecy; in some cases, being associated with other stigmatized illnesses.  Fear of certain death from cancer lead women to avoid the service. | | | | | | | **Conclusion:**  Awareness of cervical cancer screening was very low among women. The finding revealed that there are complex factors that prevented women from utilizing the cervical cancer screening.  **Policy Recommendations**  there is a need for community-based innovative and culturally sensitive interventions to improve awareness and understanding of cervical cancer which will enable women to utilize the service**.** | |
| 42 | H Bayu et al, 2016, Cervical Cancer Screening Service Uptake and Associated Factors among Age Eligible Women in Mekelle Zone, Northern Ethiopia, 2015: A Community Based Study Using Health Belief Model , Journal Article, Ethiopia | **Objective**:  To determine cervical cancer screening service uptake and its associated factors  **Study Setting**:  Kuwiha and  Mekelle towns in Mekelle Zone, Ethiopia  **Study design**:  Quantitative, cross-sectional survey  **Study population**:  1286 women over the age of 21 years living in Kuwiha or Mekelle for at least six months | **Type of analysis**:  Quantitative multiple logistic regression  **Validity (Internal)**:  The interviewers were trained and interviews were conducted using structured, pretested questionnaires. Details of training and validation not mentioned.  **Validity (External)**:  Findings not generalizable as the study population were native to a particular location  **Reliability (repetitiveness)**:  Not discussed. | | **Acceptability:**  822 (69.3%) participants felt that they themselves are susceptible to develop the condition  (90.6%) responded that they did not take the screening services because of absence symptoms  **Awareness:** (85.8%) of respondents have at least heard of cervical cancer and its screening. One thousand one hundred nine (93.5%) participants knew that cervical cancer is a killer if not detected early, but only 292(24.6%) knew that they can develop a premalignant condition of the cervix without any symptoms  1145(96.5%) of the participants agreed that cervical cancer can be severe and may be hazardous to their health  1151(97.0%) agreed precancerous cervical screening is beneficial for their wellbeing  Women who were knowledgeable on cervical cancer and its screening were about 2.355 times more likely to avail of screening services than women who were not knowledgeable (AOR = 2.355, 95%CI = 1.155–4.802)  **Angst (Fear):** (74.9%) responded that they did not take the screening services because of fear of painful test procedure | | | | | | | **Conclusion:**  Common reasons given by women for not undergoing screening were feeling of healthiness because of absent symptoms followed by emotional barriers like fear of test procedure is painful and embarrassment Age of the women, history of multiple sexual partners and sexually transmitted disease, HIV Sero-positive, Knowledge, perceived susceptibility and Perceived Barrier were important predictors of cervical cancer screening service uptake  **Policy Recommendations** | |
| 43 | Kokuro M, 2017, Factors affecting the utilization of cervical cancer screening among women attending health services in the Kumasi metropolis of Ghana, Thesis, Ghana | **Objective:** to determine factors affecting the utilisation of cervical cancer screening among women attending health services in the Kumasi metropolis of Ghana  **Study Setting:**  Kumasi South hospital and North Suntreso hospital, Kumasi metropolis of Ghana    **Study design:**  Correlational cross-sectional quantitative study  **Study population:**  369 women aged 18 years and above, attending the health clinics in the region | **Type of analysis:**  Quantitative, Pearson chi-square test    **Validity (Internal):**  Content and construct of the questionnaire were validated and a pilot study was conducted. Participants of pilot study were asked to provide feedback and experts in the field were consulted for validity testing  **Validity (External):** Questionnaires administered in hospitals. Not representative. Sample size small.  **Reliability (repetitiveness):**  Cronbach alpha test was carried out and a score of 0.8 was attained | | **Acceptability:**  Majority of participants were not sure whether cervical screening was painful (46.6%) or expensive (32%)  41.2% strongly agreed that their partners would not want them to have cervical cancer screening  **Awareness:**  75.1% had adequate knowledge on cervical cancer  88% had a good perception of cervical cancer and cervical cancer screening | | | | | | | **Conclusion:**  Most of the participants were young, single and had some form of education. The majority of them were employed and had only one child. Participants had adequate knowledge on both cervical cancer and cervical cancer screening. Also their perception level on cervical cancer screening was high. However, there was low utilisation of cervical cancer screening. Socio-demographic characteristics such as age, marital status, education level and parity of participants were found to be significantly related to the knowledge level of participants. Educational level and parity of participants were also found to be significantly related to perception levels. Lastly, marital status, work status and parity of participants were also found to be significantly related to cervical cancer screening.  **Policy Recommendations**   - Conduct further studies in other districts - Increase Public Education - Develop Programs to Target Vulnerable Group - Organisation of regular National Cervical Cancer Day - Increase cervical cancer screening centres | |
| 44 | Teame, Gebremariam, Kahsay, et al, Factors affecting utilization of cervical cancer screening services among women attending public hospitals in Tigray region, Ethiopia, 2018; Case control study, Journal article, Ethiopia | **Objective**: To determine factors affecting utilization of cervical cancer screening services among women attending public hospitals in Tigray region in 2018  **Study Setting**: 4 hospitals in the Tigray region  **Study design**: Hospital based unmatched case control study  **Study population**: Women who are sexually active, who are with in age of 21–49 years who have lived in Tigray region for the last 6 months, 312 cases and 312 controls | **Type of analysis**: Quantitative analysis – Descriptive and summary statistics, multivariate logistic regression  **Validity (Internal)**: Pretest conducted.  **Validity (External)**: Purposive sampling Data collected from hospitals in one region. Not representative.  **Reliability (repetitiveness)**: Not discussed. | | **Acceptability:** 65(20.8%) of cases and 44 (23.0%) of controls were not screened before due to low risk perception  56(17.9%) of cases and 40(20.9%) of controls were not screened before due to perceived non-necessity  **Availability:** 210(67.3%) of cases and 121(63.0%) of controls are in nearby accessible to the screening center  Seventy-three (23.4%) of case and 50(26.0%) of controls are their means of transport to cervical cancer screening center was by walk  **Awareness:** 121(38.8%) of the cases and 139(70.9%) of the controls had poor knowledge on cervical cancer  119(38.1%) of the cases and 151(77.0%) of the controls had poor knowledge on cervical cancer screening  69(22.1%) of cases and 56 (29.3%) of controls were not screened before due to ignorance | | | | | | | **Conclusion:**  Women with the age group of 30–39 and 40–49 years, current occupation with governmental and private employee, having lifetime multiple sexual partners and ever give birth were more likely to utilized cervical cancer screening.  Good knowledge about cervical cancer, good knowledge and positive attitude about cervical cancer screening were significantly associated with utilization of cervical cancer screening.  **Policy Recommendations**  There is a need to strength policy and guidelines on cervical cancer screening among women particularly with regarding occupation, number of sexual partners and parity  Health professional should give advice to women with in age group of 21–29 years, current occupation as housewife and women with single sexual partner to be screened for cervical cancer  All stakeholders, particularly the health sector, should give priority on the promotion and initiation of women to acquired good knowledge and positive attitude on cervical cancer screening. | |
| 45 | Oluwole EO, Mohammed AS, Akinyinka MR, Salako O, 2017, Cervical cancer awareness and screening uptake among rural women in Lagos, Nigeria. Journal article, Nigeria. | **Objective:**  To determine the awareness, knowledge and use of cervical cancer screening services among rural women in Lagos State, Nigeria.  **Study Setting:**  Two rural communities in Lagos State, Epe and Ikorodu  **Study design**: Cross sectional study  **Study population**: Women between 25 and 65 years of age, n=400 | **Type of analysis**:  Quantitative analysis – Univariate and bivariate analyses (Chi square test and Fisher's exact)  **Validity (Internal)**: Pre-tested structured interviewer-administered  questionnaire  **Validity (External)**: Community based but only women attending a hospital, multi-stage sampling technique. Not representative.  **Reliability (repetitiveness)**:Contamination may have occurred from exposure to media campaigns, mobile phones promoting healthy living and the on-going free cervical cancer education and screening in Lagos state by the state ministry of health | | **Approachability:** Only about a third (35.0%) of the respondents who were aware of cervical cancer got to know about it from health workers  **Awareness:** 60 (15.0%) had heard about cancer of the Cervix.  Only 17(28.3%) and 1(1.7%) of the respondents respectively were aware of Visual inspection with Acetic Acid (VIA) and Visual inspection with Lugols Iodine (VILI) as methods of screening for cervical cancer.  Overall only 5(8.3%) of the respondents had good knowledge, 23(38.4%) had fair and 32(53.3%) had poor knowledge of cervical cancer. | | | | | | | **Conclusion:**  The awareness, knowledge of cervical cancer and screening uptake are very low among women living in rural communities in Nigeria with high prevalence of the risk factors.  The practice towards prevention is also low due to the poor knowledge/ awareness of the disease.  Successful implementation of screening and primary care depend on awareness and willingness on the part of women at risk. The fact that most of the women in our study were willing to undergo screening is considered important.  **Policy Recommendations**  Community health education should be instituted  Additional equipment for screening should be made available at the primary health care centers with trained health personnel with proper referral system in place for positive cases | |
| 46 | Okunowo AA, Daramola ES, Soibi‑Harry AP, Ezenwankwo FC, Kuku JO, Okunade KS, et al, 2018, Women’s knowledge of cervical cancer and uptake of Pap smear testing and the factors inﬂuencing it in a Nigerian tertiary hospital, Journal article, Nigeria | **Objective:** To examine the knowledge of cervical cancer and uptake of Pap smear screening among women attending obstetric and gynaecological outpatient clinics at the Lagos University Teaching Hospital in Lagos, Nigeria, and the factors that affect it  **Study Setting:** Lagos University Teaching Hospital, Nigeria  **Study design:** Observational descriptive cross-sectional study  **Study population:** Women attending antenatal and gynaecological  out-patient clinics, 225 pregnant and  non-pregnant women | **Type of analysis**:  Quantitative analysis - Pearson Chi-square test, Fisher's exact test, Student's t - test  **Validity (Internal)**: Structured self-administered questionnaire, Illiterate administered by a trained interviewer.  **Validity (External)**: Study conducted in outpatient department of a hospital. Not representative data.  **Reliability (repetitiveness)**: Not discussed | | **Approachability:** Non - recommendation of the test by doctors/nurses (31.0%)  **Acceptability:** 41.5% (85) of the respondents believed that cervical cancer is a common cancer in women, but only 16.6% (34) believed that they are at risk of having the disease  The method of doing the test is embarrassing (4.4%)  Husband discouraged them from doing it (0.6%)  Believe they can't have cervical cancer (18.4%)  **Affordability:** Couldn't afford it (5.1%), Lack of time due to busy schedule (1.3%)  **Awareness:** Only 22.4% (46) of the respondents were aware that human papilloma virus (HPV) is the implicating agent in the etiology of cervical cancer  Only 40.0% (82) and 15.6% (32) of the respondents had good knowledge of the symptoms and risk factors of cervical cancer  83.9% (172) of the respondents were not aware of the vaccine used for preventing cervical cancer  55.1% (113) were aware of Pap smear, less than a third of them, 31.7% (65) had knowledge of the purpose of Pap smear.  Lack of awareness of Pap smear (53.8%)  Don't know where to do the test (15.8%)  Don't know much about the test (23.4%)  **Angst (Fear):** Fear of being diagnosed with cervical cancer (4.4%), Test is painful (0.6%)  . | | | | | | | **Conclusion:**  The knowledge of cervical cancer and the uptake of Pap smear screening are abysmally low among the women in the study.  Though a high level of formal education and literacy level are associated with increased awareness and knowledge of the disease, it had no influence on the uptake of Pap smear.  **Policy Recommendations**  Regular health education of women and recommendation of Pap smear screening by clinicians and other health care providers will go a long way to improve the uptake of Pap smear, and ultimately reduce the incidence and burden of the disease. | |
| 47 | Idowu A, Olowookere SA, Fagbemi AT, Ogunlaja OA, 2016, Determinants of cervical cancer screening uptake among women in Ilorin, North central Nigeria: A communitybased study, Journal article, Nigeria | **Objective:** To assess the determinants of cervical cancer screening uptake  among Nigerian women.  **Study Setting:** Ilorin West Local Government Area (LGA), Kwara state of Nigeria  **Study design:** Community-based, cross-sectional descriptive design  **Study population:**  All adult women who were at least 21 years of age, n=338 | **Type of analysis:** Quantitative Analysis - Chi-square test was used for bivariate analysis while binary logistic regression was used for multivariate  Analysis  **Validity (Internal):** Semi-structured interview guided questionnaire. Questionnaire translated to Yoruba and back-translated to English. Questionnaire pretested. 5 research assistants trained for data collection.  **Validity (External):** This study was conducted in urban communities; the result may not be generalizable to rural dwellers  **Reliability (repetitiveness):**Not discussed. | | **Acceptability:** Low risk perception regarding cancer of the cervix was the commonest reason for not participating in screening activities among respondents who had never been screened before; this was reported by 36.3% of such women  Husband refusal and fear of being tagged promiscuous were other reasons that could also explain the low screening uptake among our respondents  **Affordability:** Not less than 13.5% of respondents said that they could not access screening due to cost considerations  **Awareness:** 92.0% of the respondents demonstrated poor knowledge on cancer of the cervix  20% of the women interviewed had never gone for screening because they did not know where to get the services  **Angst (Fear):** 20.0% of the women who were interviewed could not go for screening for fear of positive result after screening. | | | | | | | **Conclusion:**  Most women in North Central Nigeria demonstrated poor knowledge about cervical cancer and low uptake of cervical cancer screening.  **Policy Recommendations**  Since early case detection through screening is the most cost effective activity for reducing the morbidity and mortality from cancer of the cervix, reproductive health experts and policy makers need to demonstrate more commitment in creating awareness about cervical cancer.  Need to make screening tests available at affordable costs through the establishment of more screening centres in the North Central geopolitical zone in particular and in Nigeria as a whole  There is need to integrate cervical cancer screening exercise into the mainstream healthcare services in the hospitals.  Women who are at least 21 years of age particularly those with family history of cervical cancer must be encouraged to opt for cervical cancer screening at every available opportunity.  There is need to increase the number of healthcare workers with requisite skills to conduct cervical cancer screening in Nigeria. | |
| 48 | Compaore S, Ouedraogo CMR, Koanda S, Haynatzki G, Chamberlain RM, Soliman AS, 2016, Barriers to cervical cancer screening in Burkina Faso: Needs for patient and professional education, Journal article, Burkina Faso. | **Objective:** To understand the barriers to cervical cancer early detection in Ouagadougou, the capital city of Burkina Faso  **Study Setting:**  Obstetrics and Gynaecology service of the “Centre Hospitalier Universitaire Yalgado Ouedraogo de Ouagadougou (CHUYO)”, the University Teaching hospital in Ouagadougou, the capital city of Burkina Faso  **Study design:** Cross-sectional study  **Study population:**  Women seeking screening and treatment for cervical cancer at CHUYO (n=351) | **Type of analysis:**  Quantitative analysis – Univariate and Multivariate logistic regression  **Validity (Internal)**: Pilot testing done.  **Validity (External)**: Study conducted in a gynecological clinic. Sample may not be fully representative of the female population which could limit the generalizability of results.  **Reliability (repetitiveness)**:Not discussed | | **Approachability:** Women encouraged to have screening by medical reasons (advice from healthcare professional or symptoms) were twice more likely to get screening than those with non-medical encouragement (relatives or friends’ advice).  **Acceptability:** Negligence = 5.25%  **Availability:** Distance from the screening center = 4.7%  Women living in urban areas had higher odds of screening compared to those leaving in semi-urban areas; and women in semi-urban areas had greater odds of being screened compared to rural women.  **Awareness:** The lack of awareness about cervical cancer and its screening was the main barrier (51%) to cervical cancer screening in the study participants  Less than one quarter (17.5%) of participants knew about HPV vaccine  9% were not aware of where screening is done  **Angst (Fear):** Fear of being diagnosed with cancer = 5.7% | | | | | | | **Conclusion:**  Low awareness and socioeconomic barriers lead to underutilization of screening services of women.  **Policy Recommendations**  Motivation and education by healthcare workers are important factors for increasing screening rates.  Organized patient and professional education programs in gynecologic services are warranted for improving screening in Burkina Faso and other low-resources countries in Africa. | |
| 49 | Shiferaw S, Addissie A, Gizaw M, Hirpa S, Ayele W, Getachew S, Kantelhardt EJ, Assefa M, Jemal A, 2018, Knowledge about cervical cancer and barriers toward cervical cancer screening among HIV-positive women attending public health centers in Addis Ababa city, Ethiopia, Journal article, Ethiopia | **Objective:** To assess knowledge about cervical cancer symptoms, prevention, early detection, and treatment and barriers to screening among HIV-positive women attending community health centres for HIV-infection management in Addis Ababa.  **Study Setting:** 14 randomly selected  community health centres without cervical cancer screening service in Addis Ababa.  **Study design:** Facility-based cross-sectional study design with a mixed approach (both quantitative and qualitative).  **Study population:** 581 HIV-positive  women aged 21–64 years’ old | **Type of analysis:**  Quantitative analysis - univariate analysis, binary logistic regression & multiple regressions  Qualitative analysis - thematic analysis  **Validity (Internal):** Data were collected via face-to-  Face interviews, minimizing the likelihood of misunderstanding the questions. Interviewer bias, social desirability bias  **Validity (External):** Study excludes HIV-positive  women who receive HIV care from private  healthcare institutions  **Reliability (repetitiveness):** Use of mixed methods  for triangulating the quantitative findings by the qualitative  findings. | | **Approachability:** 481 (82.8%) responded that no one recommended them to undergo cervical cancer screening  Health care provider did not have good knowledge (1.4%)  **Acceptability:**  Felt embarrassed (5.5%)  Felt they were healthy and did not need cervical cancer screening (36.5%)  Partner not supportive (1.4%)  **Availability:**  No health facility in the catchment area (4.3%)  **Affordability:**  498 (85.7%) reported being willing to undergo cervical cancer screening if the screening service was free  Felt screening was expensive (5.7%)  **Appropriateness:**  No appropriate care at health care facility (6.2%)  Hostile attitude of health care provider (0.7%)  **Awareness:**  Only 23.4% of the study participants were knowledgeable about cervical cancer symptoms, prevention, screening and treatment  Said that they don’t know where screening is done (20.1%)  **Angst (Fear):** Fear of positive results (5.3%), Fear of pain (1.2%) | | | | | | | **Conclusion:**  Knowledge about cervical cancer is poor among HIV-positive women attending public health centers in Addis Ababa, Ethiopia, with less than a quarter of them being knowledgeable about the disease and a third of them could not mention any one of common cervical cancer symptoms.  Lack of provider recommendations is a major barrier to cervical cancer screening in the city  **Policy Recommendations**  Findings underscore the need to scale up health education about cervical cancer prevention and early detection among HIV-positive women as well as among primary healthcare providers in Addis Ababa for successful expansion of cervical cancer screening in the city and perhaps in other parts of the country | |
| 50 | Gatumo M, Gacheri S, Sayed AR, Scheibe A, 2018, Women’s knowledge and attitudes related to cervical cancer and cervical cancer screening in Isiolo and Tharaka Nithi counties, Kenya: a cross-sectional study, Journal article, Kenya | **Objective**: To assess women’s knowledge and attitudes towards cervical cancer and cervical cancer screening and prevention among women aged 18 years and above in the Isiolo and Tharaka Nithi counties of eastern Kenya  **Study Setting:** Isiolo and Tharaka Nithi counties in Kenya  **Study design**: Cross-sectional survey  **Study population**: 451 women 18 years of age and older participated in the study. | **Type of analysis**: Quantitative analysis - Bivariate and multivariate analyses  **Validity (Internal)**: The self-report nature, although facilitated, might have caused bias and over- or under-estimation of certain variables  **Validity (External)**: Sample was predominantly rural and may not be generalisable to other populations. The inclusion of more women who were educated, working and married may limit the generalisability of findings to women in these areas who are less educated, unemployed and/ or single.  **Reliability (repetitiveness)**: The Cronbach’s alpha for the attitude scale was acceptable. Measurements of attitudes and knowledge unvalidated. | | **Approachability:** **Acceptability:** Cervical cancer would threaten relationship with boyfriend, husband or partner (204/360)  Have other problems more important than having cervical exams (66/360)  Prefer a female health worker to conduct cervical exams (201/360)  Ashamed to lie on a gynecologic examination table and show private parts during a cervical exam (61/360)  **Availability:** No health center close to the house to have cervical exams (100/360)  **Affordability:** Will never have cervical exams if have to pay for it (145/360), Cervical exams take too much time (43/360)  **Appropriateness:** Health care workers doing cervical exams are rude to women (34/360)  **Awareness:** One in six participants (16.9%, 61/360) knew that HPV is a risk factor for cervical cancer  15.1% (68/451) had heard of HPV  **Angst (Fear):** Having cervical exams is too painful (71/360) | | | | | | | **Conclusion**  Overall knowledge of risk factors for cervical cancer among women in Isiolo and Tharaka Nithi counties was low.  Lack of awareness of cervical cancer and knowledge of risk factors are likely barriers to accessing cervical cancer screening services and related care.  **Policy Recommendations**  Barriers should be addressed through novel multi-faceted strategies that could include the use of peer-education, mass media and interventions delivered at healthcare facilities and by community health workers.  Approaches should be tailored to each county to account for the different contexts and evaluated for effectiveness. | |
| 51 | Mabelele MM, Materu J, Ng'ida FD, Mahande MJ, 2018, Knowledge towards cervical cancer prevention and screening practices among women who attended reproductive and child health clinic at Magu district hospital, Lake zone Tanzania: a cross-sectional study, Journal article, Tanzania | **Objective:** To determine the knowledge of cervical cancer prevention services and screening practices among women who attended Reproductive Child Health clinic at a district hospital  **Study Setting:** Magu district hospital in Lake Zone, Tanzania  **Study design:** Cross-sectional study  **Study population:** 307 women of reproductive age (15 – 49 years) attending reproductive and child health clinic | **Type of analysis**:  Quantitative analysis – Multivariate logistic regressions model  **Validity (Internal)**: Study relied on self-reported screening practices which  may be subject to reporting bias or self-desirability bias  **Validity (External)**: This study was hospital-based,  recruiting only women attending RCH clinic and may not be representative of the whole community.  **Reliability (repetitiveness)**: High quality analysis technique | | **Awareness:** Only 17.6% of women were able to mention at least one cervical cancer risk factor  Only 53 (17.3%) women had adequate knowledge on cervical cancer. | | | | | | | **Conclusion:**  Majority of women lack comprehensive knowledge of cervical cancer and only few utilize screening services  **Policy Recommendations**  Strategies for awareness creation about cervical cancer may help to improve knowledge and utilization of cancer screening practices. | |
| 52 | Bai Y, Liu Q, Chen X, Gao Y, Gong H, Tan X, Zhang M, Tuo J, Zhang Y, Xiang Q, et al, 2018, Protection motivation theory in predicting intention to receive cervical cancer screening in rural Chinese women, Journal article, China | **Objective**: To test the protection motivation theory (PMT) in predicting screening intentions  **Study Setting**: 10 randomly selected villages from Wufeng county, Hubei province, China  **Study design**: Standardized questionnaire and Structural equational modelling  **Study population**: Rural (n=3000) women aged 35-65 with no cervical cancer history and all of them sexually active | **Type of analysis**: Quantitative analysis – Chi-square test, Pearson correlation, MLE for structural equation model, Mardia’s kurtosis test  **Validity (Internal)**: Data used for this study is cross-sectional in nature. A binary variable (yes/no) was used to measure intention to participate in screening and awareness, In-person interview, pilot-tested questionnaire.  **Validity (External)**: Participants were selected through multi-stage stratified cluster sampling  **Reliability (repetitiveness)**: High quality analysis technique | | **Awareness:** 26.6% (798) had not heard of cervical cancer screening  29.1% (873) felt that postmenopausal women do not need cervical cancer screening  Only 45.2% (1356) felt that women should receive cervical cancer screening regularly  Only 8.5% (255) were of the opinion that not using a condom during sex is a risk factor for cervical cancer  30% (900) had no knowledge about the risk factors of cervical cancer  Only 26.77% (803) women knew that having more than one sex partner is a risk factor for cervical cancer | | | | | | | **Conclusion:** Awareness of and experience with screening services can be translated into screening intentions by altering rural women’s perceptions of risks not screening and costs associated with screening  **Policy Recommendations**  Effective educational program must emphasize both current knowledge of cervical cancer risk and the awareness of and prior experience with screening services.  A number of measures can be taken to reduce perceived cost for screening. Typical examples include more efficient communication and explanation to women to reduce negative feelings associated with screening, promoting best practice in conducting screening, and use of female physicians to provide screening. | |
| 53 | Dey S, Sharma S, Mishra A, Krishnan S, Govil J, Dhillon PK, 2016, Breast cancer awareness and prevention behavior among women of Delhi, India: identifying barriers to early detection, Journal article, India | **Objective:** To explore the awareness and perceived barriers concerning the early detection of BC.  **Study Setting**: Delhi, India  **Study design:** Qualitative study - FGDs  **Study population:** 194 women ages ranging from 18-70 years | **Type of analysis:** Qualitative analysis – Thematic analysis  **Validity (Internal):** FGD guide followed. Conducted by an experienced researcher. Hindi verbatim has been translated into English language and reported alongside the Hindi verbatim. Echo questions were made by the researcher to confirm meaning of the par­ticipants’ response to ensure internal validity.  **Validity (External):** Participants for the FGDs were selected using the convenience sampling method. Diverse sections of the society included.  **Reliability (repetitiveness):**  After comple­tion of each audio-taped FGD’s transcription, recordings were played for the second time to make them reliable | | **Approachability:** In private hospitals they are snagging money out of you like hell  **Acceptability:** Shyness, hesitation, and not prioritizing self-health  Presence of a male doctor  A few women mentioned that they feel shyness/hesitation even in the presence of a lady doctor  Hesitation to inform male member present in the family was also found to be one of the reasons to avoid approaching doctors  Compared to women, girls have more hesitation to discuss breast issues  Women do not give priority to their health issues -Ignorance, negligence, carelessness, and bearing pain  Lack of family support  Majority of women wait and watch for automatic cure and tend to approach the health system only in critical health situations.  **Affordability:** Many people don’t have money for the treatment so keep on avoiding the symptom,  Going to hospital results in absenteeism from work and consequently no payment. Then kids have to sleep hungry.  **Appropriateness:** Condition of government hospitals is very bad  **Awareness:** General misconceptions such as: Stressed out women may get BC, Aged people can be affected, married women are prone to get BC, BC occurs in adolescent period, Pain is the initial symptom, if women feel pain, only then they should get checked-up.  Negligible women reported that they know about the detection methods, including BSE, mammography, and CBE  Women from slum areas believed that BC is contagious  Majority of women linked BC with complete removal of breast and loss of femininity  Women had a strong perception that it cannot be cured  **Angst (Fear):** Fear - women perceived cancer as a death  Fear of detecting any big health problems  Fear from getting spoiled relationship with husband and other family members  Stigma - people consider BC sexual disease and result of the wrong they did  Fear of pressure and blame from husband | | | | | | | **Conclusion:** The study enumerated the barriers that prevent women from visiting the health system so that BC is detected early. Some of the issues related to lack of awareness, fear, familial and social stigma, myths and misconceptions, neglect and lack of priority given to health by women, etc. were captured.  **Policy Recommendations**  Using media and including men while creating awareness about women’s issues are quite important for effective BC control. | |
| 54 | Mitiku I, Tefera F, 2016,  Knowledge about Cervical Cancer and Associated Factors among 15-49-Year-Old Women in Dessie Town, Northeast Ethiopia, Journal article, Ethiopia | **Objective:** To assess women's knowledge about cervical cancer and associated factors  **Study Setting**: Dessie town, Northeast Ethiopia    **Study design**: Community based cross-sectional survey  **Study population**: 620 women aged 15±49 years | **Type of analysis:**  Quantitative Analysis - Binary and multiple logistic regressions  **Validity (Internal):** pre-tested structured questionnaire developed in English and translated to Amharic. Backward translation done. Administered by trained health workers via face-to-face interviews. Supervisors supervised the workers.  **Validity (External):** Respondents were selected  using a multistage sampling technique. The study was conducted in an urban setting, which may limit generalizability of the findings to all women in Ethiopia, particularly to those in rural areas  **Reliability (repetitiveness):** The reliability of items of the scale was evaluated  using Cronbach’s alpha.  a high internal consistency was found in the present study (Cronbach’s α  = 0.95). High quality analysis technique used. | | **Awareness:** 262 (42.3%) of the women had not heard about Cervical cancer  About 58.1% of the respondents did not know whether there are risk factors for cervical cancer or not.  333 (53.7%) of the respondents did not know any symptom of cervical cancer  365 (58.9%) of the respondents did not know any cervical cancer prevention measure  45% of respondents had not heard of cervical cancer screening | | | | | | | **Conclusion:** This study shows a suboptimal knowledge about cervical cancer regarding its risk factors, signs and symptoms, prevention and treatment among women in the study site.  The level of education and economic status were found to be important determinants for knowledge about cervical cancer.  **Policy Recommendations**  Prevention programs should focus cervical cancer educational resources on women with less education and women with lower economic status groups | |
| 55 | Bora K, Rajbongshi N, Mahanta LB, Sharma P, Dutta D, 2016, Assessing the awareness level of breast and cervical cancer: a cross-sectional study in northeast India, Journal article, India | **Objective:** To study the awareness of breast and cervical cancers among common women based on different factors such as age, residence, and occupation  **Study Setting:**  Not mentioned – assumed to be community based  **Study design:** Cross-sectional study  **Study population:** 1000 women | **Type of analysis:**  Quantitative Analysis - Chi-Square test, MANOVA test, post hoc Duncan test  **Validity (Internal):**  Pretested in pilot but details not described  **Validity (External):** Selective sampling  **Reliability (repetitiveness):**  Pretested questionnaire | | **Awareness:**  Rural women showed less knowledge compared with urban women and housewives were less aware  In breast cancer survey, women who were found less aware belonged to the age subgroup 18–25 years; but, in the case of cervical cancer, women in the age subgroup above 45 years showed less knowledge | | | | | | | **Conclusion:** Knowledge of breast cancer and cervical cancer among common women was very poor  **Policy Recommendations**  The awareness level in the society has to be improved through different initiatives such as awareness camps and by educating the common women. | |
| 56 | Ndejjo R, Mukama T, Musabyimana A, et al, 2016, Uptake of cervical Cancer screening and associated factors among women in rural Uganda: A Cross Sectional Study, Journal article, Uganda | **Objective:** To assess uptake of cervical cancer screening and associated factors among women in rural Uganda  **Study Setting:** Bugiri and Mayuge districts in eastern  Uganda  **Study design:** Descriptive cross sectional study using semi-structured questionnaire  **Study population:** Females aged between  25 and 49 years who had spent six or more months in the area, n= 900 | **Type of analysis**:  Quantitative analysis - Univariate, bivariate and multivariate analyses  **Validity (Internal)**: Being a cross sectional study, it is not possible to assess causality. Cervical cancer screening status was self-reported and could have been affected by social desirability. Questionnaire translated from English to Lusoga and back-translated to English. Questionnaire pre-tested. Administered by trained research assistants.  **Validity (External):** This study was carried out in two majorly rural districts and therefore the findings may not be generalizable to other contextually different areas.  **Reliability (repetitiveness)**: Potential bias was minimised by asking respondents to provide dates when they  accessed the service and duration since they last accessed it, which ensured reliability | | **Approachability:** Long waiting times at facilities  **Acceptability:** 553 (64.5%) stated personal perception related reasons having no signs and symptoms of the disease, not being at risk  **Availability:** 142 (16.6%) stated health facility related challenges like distance  **Affordability:** Costs, lack of time  **Awareness:** Not aware of cervical cancer screening services = 416 (48.5%)  **Angst (Fear):** fear of test outcomes | | | | | | | **Conclusion:** The study found a very low level of cervical cancer screening among women in rural Uganda  The barriers to screening identified included not being aware of cervical cancer screening services, health facility related challenges such as distance to health facilities and costs of the service, and individual perceptions related to; having no signs and symptoms of the disease, not being at risk, lack of time and fear of test outcomes  The independent predictors for cervical cancer screening were being recommended for screening by a health worker, knowing where cervical cancer screening services were offered and knowing someone who had ever been screened for the disease.  **Policy Recommendations**  Cervical cancer screening can be increased by utilising health workers to discuss the disease with women when they go to seek health care. | |
| 57 | Mukama T, Ndejjo R, Musabyimana A, et al, 2017, Women’s knowledge and attitudes towards cervical cancer prevention: a cross sectional study in Eastern Uganda, Journal article, Uganda | **Objective:** To assess the knowledge and attitudes of Ugandan women about cervical cancer prevention with the aim of informing prevention and control interventions  **Study Setting:** Bugiri and Mayuge districts in eastern Uganda.  **Study design:** Cross-sectional community based survey and collected data by means of a questionnaire  **Study population:** 900 women aged 25–49 years | **Type of analysis**:  Quantitative analysis - Bivariate and multivariate analyses  **Validity (Internal)**: Lack of a standardised knowledge assessment Questionnaire, administered by research assistants. Questionnaire translated to Lusoga and back-translated to English  **Validity (External)**: Community based cross-sectional study, multi-stage sampling technique was used  **Reliability (repetitiveness)**:Lack of standardised questionnaire. | | **Awareness:** Only 7 (0.01%) respondents knew the recommended frequency for cervical cancer screening  More than half of respondents (489; 54.3%) did not know any methods used for screening for cervical cancer.  154 (17.1%) did not know any method of preventing the disease  Most respondents (556; 61.8%) believed that nothing could be done once someone is diagnosed with cervical cancer | | | | | | | **Conclusion:** This study found relatively high knowledge about general cervical cancer prevention but specific knowledge about screening was very low.  **Policy Recommendations**  Findings suggest that integrating HIV counselling and testing services with cervical cancer services would enhance awareness about the disease among women.  Need for more awareness campaigns to provide comprehensive information about cervical cancer screening to women in all areas and dispel any negative beliefs and perceptions | |
| 58 | Ndejjo R, Mukama T, Musinguzi G, et al, 2017, Women's intention to screen and willingness to vaccinate their daughters against cervical cancer - a cross sectional study in eastern Uganda, Journal article, Uganda | **Objective:** To assess the factors associated with intention to screen for cervical cancer among women in eastern Uganda, and willingness to have their daughters vaccinated against the disease  Study Setting: Bugiri and Mayuge districts in eastern  Uganda  **Study design**: Cross sectional study using a pretested semi-structured questionnaire  **Study population**: 900 females aged 25 to 49 years | **Type of analysis:** Quantitative analysis – Linear regression, Poisson distribution  **Validity (Internal):** Majorly rural population, and had a large sample size, possible social desirability bias  the tendency for study participants to give socially desirable responses which could have introduced bias. Data were collected using a pretested semi-structured  questionnaire that was developed in English, and then translated to Lusoga, the questionnaire was administered by a team of trained Research Assistants  **Validity (External):** Multi-stage sampling used to select counties in each district, systematic random sampling to select households in each county  **Reliability (repetitiveness):** Not discussed | | **Acceptability:** 28 stated that screening was not necessary or important  lack of signs and symptoms  61 reported unwillingness to vaccinate daughters  **Availability:** 9 cited health facility being far away  **Affordability:** 6 participants felt high costs involved in screening  **Angst (Fear):** fear of pain | | | | | | | **Conclusion:** This study assessed the socio-demographic, knowledge, attitudinal and health facility factors associated with women’s intention to screen, and willingness to vaccinate their daughters against cervical cancer in eastern Uganda.  **Policy Recommendations**  Therefore, increasing access to cervical cancer screening services and further education of women is expected to increase uptake rates of screening services which are currently low in developing countries including Uganda  As there is high willingness to vaccinate daughters, the focus of various stakeholders such as the Ministry of Health and Non-governmental Organisations should be turning this into actual practice through increasing service availability and accessibility by using effective strategies such as schools, health centres, and community based programmes | |
| 59 | Acharya Pandey R, Karmacharya E, 2017, Cervical cancer screening behavior and associated factors among women of Ugrachandi Nala, Kavre, Nepal, Journal article, Nepal | **Objective:** To assess the cervical cancer screening behaviour and its associated factors among women of Nala Village Development Committee (VDC), Kavre  **Study Setting:** Nala Village Development Committee (VDC), Kavre  **Study design:** Descriptive cross-sectional study  **Study population:**  180 women between the ages of 30 & 60 years | **Type of analysis**:  Quantitative analysis - Descriptive and inferential statistics (Chi-square test)  **Validity (Internal)**: As the respondents had to remember when they had performed cervical cancer screening, there might have been recall bias. association between perceived  susceptibility and cervical cancer screening behavior  could not be assessed because of error caused by small sample size for Chi-square test.  **Validity (External)**: Quantitative descriptive study design. Not generalisable.  **Reliability (repetitiveness)**:  Not discussed | | **Acceptability:** Cervical cancer screening will be embarrassing to me = 65% (117/180)  Undergoing cervical cancer screening will make me worry about cervical cancer = 45% (81/180)  Cervical Cancer Screening will interfere with my family obligations = 14.4% (26/180)  I prefer females perform cervical cancer screening because it is uncomfortable to me if a man does it = 87.8% (168/180)  **Affordability**: Cervical cancer screening is too expensive = 30.6% (55/180)  Undergoing cervical cancer screening will take too much time = 23.3% (42/180)  **Awareness:** I don’t know where I could go if I wanted cervical cancer screening = 62.2% (112/180)  **Angst (Fear):** Cervical cancer would threaten a relationship with my husband or partner = 72.3% (130/180)  Cervical Cancer Screening procedure will be painful = 52.8% (95/180) | | | | | | | **Conclusion:** The major reason for cervical cancer screening was health personnel’s advice.  Minority of women perceived themselves at risk of cervical cancer.  Majority of women felt uncomfortable to be screened by male health personnel.  No significant association was found between cervical cancer screening behavior and age, parity, perceived severity, perceived benefits, and perceived barriers.  **Policy Recommendations**  Awareness campaigns targeting illiterate groups can be conducted in community so that they become motivated towards cervical cancer screening.  Health professionals can conduct health education programs to the women eligible for screening attending gynecological clinic regarding prevention of cervical cancer and importance of cervical cancer screening. | |
| 60 | Adewumi, K., Oketch, S.Y., Choi, Y., Huchko, M.J., 2019, Female perspectives on male involvement in a human-papillomavirus-based cervical cancer-screening program in western Kenya, Journal article, Kenya | **Objective**: To examine the role of male partners and community leaders in decision-making and accessing screening services  **Study Setting**: Western Kenya  **Study design**: Semi-structured, in-depth interviews (IDIs)  **Study population**: Women and community health volunteers, n=604 | **Type of analysis**:  Qualitative analysis – Thematic analysis  **Validity (Internal)**: Semi-structured, in-depth interviews, triangulation of data, IDIs conducted in English, Dholuo and Kiswahili. Interviews  were confidential, there remains the possibility  of a social response bias  **Validity (External)**: Community health volunteers and different categories of women but from the same town. While the data can be representative of the cross-section, it’s not representative of the whole region.  **Reliability (repetitiveness)**: Triangulation of data, high reliability | | **Acceptability:** Many women remained reluctant to seek screening without first obtaining their partner’s permission due to perceived male partner distrust and stigmatization  Their partners did not take actions supportive of their attempts to seek treatment, such as encouragement, childcare or transport money  Unable to disclose their screening attendance or test results to their partners, received negative responses when/ if they did choose to disclose  Unwilling to provide funds for transportation or women felt unable to disclose their need for transportation money  Women were worried that partners would not believe the instructions from health professionals such as abstinence or wear condoms during sex  Women who were considered lost-to follow-up tended to cite their partners as barriers to treatment more often than others.  **Awareness:** Women who expressed a desire for increased male involvement in cervical cancer prevention, also perceived male partners to have limited knowledge of prevention.  Angst (Fear): Stigmatizing attitudes toward HPV included an association with promiscuity, infidelity, and HIV. | | | | | | | **Conclusion:** Increased male knowledge will lead to increased uptake of cervical cancer screening and prevention services  The data presented in this paper as well as the developed framework may begin to lay the foundations for (1) the assessment of potential barriers and facilitators within a cervical cancer prevention cascade and (2) the identification of an important factors that may define male involvement in cervical cancer prevention.  **Policy Recommendations**  Educating male partners may equip men to better care for and interact with their partners; this included  (1) permission/ encouragement to get screened or treated, (2) tangible support such as financial support for transport, (3) a reduction in partner distrust, and (4) an increase in adherence to post-treatment care | |
| 61 | Huchko, M., Adewumi, K., Oketch, S., Saduma, I., Bukusi, E., 2019, ‘I’m here to save my life’: a qualitative study of experiences navigating a cryotherapy referral system for human papillomavirus-positive women in western Kenya, Journal article, Kenya | **Objective:** To understand the beliefs,  social norms and logistical factors that affect human  papillomavirus (HPV)-positive women’s uptake of cryotherapy treatment as part of a two-part cervical  cancer screening strategy in rural Kenya.  **Study Setting:** Government-run county hospital in western  Kenya.  **Study design:** In-depth interviews within a parent cluster randomised  Trial  **Study population:** 273 of 372 (73.4%) HPV-positive women  who underwent cryotherapy | **Type of analysis**:  Qualitative analysis – Thematic analysis  **Validity (Internal)**: Women were interviewed by trained study staff, so there may have been social desirability bias.  **Validity (External)**: Cluster-randomised trial. Data collected from 12 communities between 12 and 94 kilometres from each other. exploratory, and may  not be reflective of larger patterns or associations  **Reliability (repetitiveness)**: Women lost to follow up not covered in the study. | | **Acceptability:** Few women expressed concern about cultural and social beliefs regarding sexual activity that would prevent their husbands from supporting them through the recovery period, or use of a condom afterwards.  Lack of financial support for transportation from partner  Lack of moral support for treatment from partner  Concerns about the ability to be abstinent (because of lack of understanding from the husband) following treatment  **Availability:** Travelled a long distance, lack of treatment facilities closer to home in rural areas or lack of mobile screening units,  **Affordability:** Lack of transportation Funds reimbursement from the state for transportation  **Awareness:** Women reported believing that family planning methods caused their positive HPV result or would cause cervical cancer  Some women continued to believe that a positive HPV result was synonymous with having cancer | | | | | | | **Conclusion:** Despite multiple efforts to counsel women about the importance and availability of treatment, over half of the women who tested HPV-positive in the parent study did not access treatment. We have identified specific barriers and potential facilitators to treatment access that will inform new implementation strategies and ways to intensify efforts to reach the wider population of women who were lost to follow-up and work with healthcare teams to develop a linkage to treatment strategy that ensures greater follow-up with appropriate care.  **Policy Recommendations**  The role of partner support needs to be explored among women who were not able to obtain treatment.  The frequency at which the costs of  transportation to a distant treatment site and the reliance on partners were reported indicates a need to explore the decentralisation of treatment with or without a mobile treatment unit, the use of transportation vouchers or assistance of some type that emphasised transportation  The use of peer educators to help encourage and facilitate treatment access may also be a strategy to overcome the logistical hurdles using an empowerment framework | |
| 62 | Lieber, M., Afzal, O., Shaia, K., Mandelberger, A., Du Preez, C., Beddoe, A.M., 2019, Cervical cancer screening in HIV-positive farmers in South Africa: Mixed-method assessment, Journal article, South Africa | **Objective**:  To determine the quality and sustainability of an implemented See and Treat cervical cancer screening program  **Study Setting**:  Local HIV clinic in Limpopo, South Africa  **Study design**:  Log charts of patient cohort n=403, Eighteen in-depth interviews  **Study population**: Health providers, ancillary health workers, and patients. | **Type of analysis**:  Mixed-methods program  **Validity (Internal)**: Accuracy and consist­ency in record keeping was lacking, Document review content was unreliable and pos­sibly biased. 237 of the original 403 participants who were screened using VIA (59%) were lost to follow-up.  **Validity (External)**: Interviewees were recruited at the clinic using convenience sampling, Not generalizable.  **Reliability (repetitiveness)**:  Mixed methods study design | | **Approachability:** Waiting for many hours, Uninformed about practices and procedures  **Acceptability:** About 10–20% refuse Pap smear because they concerned with privacy.  **Availability:** Few See and Treat clinics which have the necessary facilities  **Appropriateness:** Hospital is understaffed  High rate of turnover among top administrators and lower level workers  Nurses need more education  **Angst (Fear):** too nervous | | | | | | | **Conclusion:** Increased awareness of cervical cancer among health workers and participants and successful integration of See and Treat into a clinic in rural South Africa.  Following guidelines for triaging of positive screens, abnormal results can be readily addressed and treated promptly  **Policy Recommendations**  The results of this assessment will inform ongoing cervical cancer screening programs in low-resource settings and with similar patient populations by highlighting the successes and barriers that must be addressed for successful and widespread program implementation  Using HPV screening with triage to VIA in the same setting will allow us not only to widen our screening coverage, but also to readily treat patients where the migrant nature of the population accounts for high rates of loss to follow-up. | |
| 63 | Manga, S., Kiyang, E., DeMarco, R.F., 2019, Barriers and facilitators of follow-up among women with precancerous lesions of the cervix in Cameroon: A qualitative pilot study, Journal article, Cameroon | **Objective**:  To explore and describe the barriers and facilitators to follow-up for cervical precancer among women infected and uninfected with HIV in Cameroon.  **Study Setting:**  2 Women’s health program (WHP) clinics in the French speaking region of Cameroon  **Study design:**  Qualitative research design  In-depth individual patient interviews (IDIs) and focus groups with nurses  **Study population:**  Eight HIV-infected and -uninfected women diagnosed with cervical precancer and 19 nurses | **Type of analysis**:  Qualitative analysis -  Thematic analysis, six-step approach of Maguire and Delahunt  **Validity (Internal)**: Triangulation of data, in-depth interviews,  **Validity (External)**: Conducted in 10 clinics in 6 geographical regions. only women from urban cities participated. Nurses from both urban and semi-urban regions participated. Purposive sampling. Small sample size.  **Reliability (repetitiveness)**:  Only French-speaking regions of the country covered. The English speaking regions not covered. | | **Approachability:** Inadequate counselling or Wrong advice - She went for confirmation to another clinic and the gynecologist she met there asked her to go and buy the HPV vaccine, Gardasil, and that when she takes it, it will heal her or cure her from that precancer and she took the first dose at a 150,000 frs ($300)  **Acceptability:** Male partner’s influence - I have had cases where clients will always want to get their spouses informed before they get treatment. … And there are situations where they need the husband’s approval because of finances, because most at times they are the ones to give the money  Alternative treatment - She actually came with her husband and told us that she went for prayers and the pastor told her that she is healed,  So many of them have been taking these herbs and supplement and later came back with cancer. Some even at the level of invasive cancer, still go back to the herbs  **Availability:** Distance - There are some of these women that you see in very remote areas who are like, they are ready to come for their treatment, but the transport to take them from the villages to the clinic especially those for LEEP is not there, even if you are ready to do the LEEP for free, they don’t even have the means to get to the health facility  **Affordability:** Poverty - some people are scared about having to come back, maybe they have been given treatment without payment or have paid just part of the treatment and they have to come back to complete the amount and they don’t have the full money, so they are scared of coming back, cost of services  **Appropriateness:** Staff attitude - This client had been a regular client in the clinic … and she had been well received … But for her to come this time to see somebody who is too slow, very sluggish, and really not explaining things the way they had explained to her before  Maybe if I was reminded. Honestly, I just removed everything in my mind like that since I didn’t have any pain or symptom that made me feel something was wrong with me. So, I didn’t think about coming for follow-up. … and I think what could be done is for the staff who are there to call, remind the patients to be able to come  **Awareness:** Lower levels of education - I have noticed that the educated women most at times, come more often for their follow-up  **Angst (Fear):** She didn’t think that all the side effects like the vagina discharge, she wouldn’t be able to bear. Other clients too have not come for their treatment because they are afraid that it may lead to infertility | | | | | | | **Conclusion:** Four themes emerged from the interviews: clinic, personal, and social barriers, and strategies to overcome them.  **Policy Recommendations**  The same study needs to be conducted among women in rural areas and especially among women who were initially screened in mobile clinics in remote villages to find out what challenges are peculiar to them  To improve follow-up which include fee reduction, reminder phone calls, use of souvenirs, and assisting poorer women | |
| 64 | Maree, J.E., Kampinda-Banda, M., 2018, Knowledge and practices of cervical cancer and its prevention among Malawian women, Journal article, Malawi. | **Objective:** To describe the knowledge and practices of cervical cancer and its screening as well as the educational preferences of women living in a rural community  **Study Setting:** Chiradzulu District, Malawi  **Study design**: Structured interviews  **Study population**: Women between the ages 30 and 45, (n = 282) | **Type of analysis**:  Quantitative analysis – Chi-square test  **Validity (Internal)**: Self-report data were collected, which could have led to social desirable and recall bias and guessing  **Validity (External)**: Study was conducted in one rural area, Generalisation cannot be done.  **Reliability (repetitiveness)**:  Used content analysis to verify the quantitative responses | | **Acceptability:** 17.8 % (36/178) were too lazy to go for the screening  9.4% (19/178) felt no need to undergo screening  8.9% (18/178) were unwilling to be screened  4.5% (9/178) were too busy to go for screening  **Appropriateness:** 4.5% (9/178) went for screening but not screened due to failure to provide the service  **Awareness:** 25.9% (64/247) did not know the causes and symptoms of cervical cancer  42.1% (75/178) did not know about the cervical screening programme  29.2% (59/178) lacked knowledge about cervical screening  **Angst (Fear):** 11.9% (24/178) had fear of screening  3.5% (7/178) lack courage to go for screening | | | | | | | **Conclusion:** Study provided evidence that most of the women had heard of cervical cancer and believed cervical cancer could be cured if detected at an early stage. However, their knowledge of the causes and symptoms of the disease was poor and many did not believe they were at risk for developing cervical cancer.  Most of the women had heard of the VIA screening programme, but less than a quarter had ever been screened. A lack of knowledge about this programme was presented as the most common reason for not being screened. Most were willing to be screened if they were reassured that VIA was a simple, painless procedure  **Policy Recommendations** | |
| 65 | Nyambe, A., Kampen, J.K., Baboo, S.K., Van Hal, G., 2019. Knowledge, attitudes and practices of cervical cancer prevention among Zambian women and men, Journal article, Zambia | **Objective:**  **Study Setting:** Chilenje and  Kanyama (two townships in the capital city Lusaka)  **Study design:** Cross-sectional mixed methods study  **Study population:** women (N = 300) and men (N = 300) | **Type of analysis:**  Mixed Methods  Quantitative analysis - Chi-square tests and Fisher’s exact tests  Qualitative analysis  **Validity (Internal):** The questionnaires were not self-administered; it may have led to biases in respondents because cervical cancer may be considered a sensitive topic  **Validity (External):** The location of the study Chilenje and Kanyama townships not representative of the whole of Lusaka, Zambia  **Reliability (repetitiveness):** Triangulation of data, high reliability, mixed method design. | | **Acceptability:** women’s perceived approval of partners, family and friends influenced their screening practices  religion showed no influence on screening  decisions and showed a positive influence on vaccination  acceptance  **Awareness:**  Less than half of the respondents (36.8%) had heard of cervical cancer, 20.7% of women had attended screening and 6.7% of the total sample had vaccinated their daughter. Knowledge of causes and prevention was very low. There was a strong association between having awareness of cervical cancer and practicing screening (odds ratio = 20.5, 95% confidence interval = [9.214, 45.516]) and vaccination (odds ratio = 5.1, 95% confidence interval = [2.473, 10.423]). Social interactions were also found to greatly influence screening and vaccination behaviors  men were found to have a slightly higher level of knowledge of cervical cancer than women | | | | | | | **Conclusion:**  The low level of knowledge of causes and prevention of cervical cancer suggests a need to increase knowledge and awareness among both women and men. Interpersonal interactions have great impact on practicing prevention behaviors, for instance, vaccination of daughters.  **Policy Recommendations**   - there is a need to ensure that cervical cancer screening services are available at all clinics - Due to low levels of knowledge, perhaps having these services at all clinics may lead to a better outreach between healthcare practitioners and women who may attend the clinic even for other health reasons. | |
| 66 | Olubodun, T., Odukoya, O.O., Balogun, M.R., 2019, Knowledge, attitude and practice of cervical cancer prevention, among women residing in an urban slum in Lagos, South West, Nigeria, Journal article, Nigeria | **Objective:** To assess the knowledge, attitude and preventive practices towards cervical cancer among women living in an urban slum in Lagos, Nigeria  **Study Setting:** Idi-Araba community, Lagos, Nigeria  **Study design:** Descriptive cross-sectional study using interviewer administered questionnaires  **Study population**: 305 women  of reproductive age (15 - 49 years) who had resided in the community for at least 2 years prior to the study | **Type of analysis**:  Quantitative analysis – Frequency tables  **Validity (Internal)**: Data was collected using pretested, interviewer administered questionnaires. Were administered by three trained female research assistants  **Validity (External)**: Descriptive cross-sectional study, a multistage sampling method was used, only covered women of one community  **Reliability (repetitiveness)**:  Not disucssed | | **Acceptability:** (64.3%) did not consider themselves at risk for cervical cancer  Believed spiritual protection would protect them from cervical cancer (60.7%)  **Awareness:** Only 39 (12.8%) had heard about cervical cancer  About 90% did not know any risk factors of cervical cancer  Majority of respondents did not know of the symptoms of cervical cancer (90.8%), cervical cancer screening (92.1%) and HPV immunization (98.4%).  Not aware of screening program (91.4%) | | | | | | | **Conclusion:**  Low awareness of cervical cancer was reported among the slum dwelling women in this study  Belief in personal susceptibility was low but most participants were willing to be screened or vaccinated. However, the majority required the consent of their spouses  **Policy Recommendations**  Need for health education campaigns on cervical cancer prevention with involvement of males, as well as increasing access to cervical cancer preventive services among low resource women. | |
| 67 | Mbachu C, Dim C, Ezeoke U, 2017, Effects of peer health education on perception and practice of screening for cervical cancer among urban residential women in south-east Nigeria: a before and after study, Journal article, Nigeria | **Objective:** To assess the effectiveness of peer health education on perception, willingness to screen and uptake of cervical cancer screening by women  **Study Setting:** 2 Urban cities in Enugu state, Nigeria  **Study design:** Before and after intervention study through self-administered questionnaires  **Study population:** 300 women of reproductive age who were 21 years or more; currently sexually active or have been in the past; and never been diagnosed with any cancer | **Type of analysis**:  Quantitative analysis – Logistic Regression  **Validity (Internal)**: The questionnaire was self-administered and completed individually by the respondents  **Validity (External)**: The scope for generalization of findings of this study is limited to women residing in urban and peri-urban  areas. The study population consisted of Anglican women who belonged to the women’s fellowship, implying that some social and cultural factors  that influence behavior have already been controlled and  their effects cannot be measured.  **Reliability (repetitiveness)**:  Not discussed | | **Acceptability:** Women who felt cervical screening was not beneficial - Pre-intervention = 98/285 (34.4%), Post-intervention = 33/285 (11.7%)  Women who felt they were not at risk of cervical cancer - Pre-intervention = 211/285 (74%), Post-intervention = 170/285 (60%)  Do not have symptoms - Pre-intervention = 85/285 (29.9%), Post-intervention = 104/185 (36.7%)  **Availability:** Distance of screening center, Pre-intervention = 37/285 (13%), Post-intervention = 46/185 (16.2%)  **Affordability:** Cost of screening - Pre-intervention = 12/285 (4.3%), Post-intervention = 24/185 (8.5%)  **Awareness:** Don’t know screening tests - Pre-intervention = 118/285 (41.4%), Post-intervention = 0/185 (0.00%)  Don’t know where to screen - only Post-intervention = 81/185 (28.6%)  **Angst (Fear):** Fear of pain - Pre-intervention = 9/285 (3.2%), Post-intervention = 0/185 (0.00%)  Fear of positive result, only Post-intervention = 40/285 (14%) | | | | | | | **Conclusion**  Willingness to screen for cervical cancer is considerably high amongst the study participants.  **Policy Recommendations**  Peer health education should be included in the health promotion component of cervical cancer control plans, because it could be useful for ensuring improved cervical cancer screening behavior | |
